# Design, Synthesis, and Biological Evaluation of Novel Urea-Containing Carnosic Acid Derivatives with Anticancer Activity

**DOI:** 10.3390/ijms252413332

**Published:** 2024-12-12

**Authors:** Sara P. S. P. Moura, Silvia Marín, Ismael Rufino, Rita C. Guedes, Marta Cascante, Jorge A. R. Salvador

**Affiliations:** 1 Laboratory of Pharmaceutical Chemistry, Faculty of Pharmacy, University of Coimbra, 3000-548 Coimbra, Portugal; spmoura17@gmail.com; 2 Center for Neuroscience and Cell Biology (CNC), University of Coimbra, 3004-504 Coimbra, Portugal; 3 Department of Biochemistry and Molecular Biomedicine, Faculty of Biology, University of Barcelona, 08028 Barcelona, Spain; silviamarin@ub.edu; 4 Centro de Investigación Biomédica en Red de Enfermedades Hepáticas y Digestivas (CIBEREHD), Instituto de Salud Carlos III (ISCIII), 28029 Madrid, Spain; 5 Research Institute for Medicines (iMed.ULisboa), Faculty of Pharmacy, University of Lisboa, 1649-003 Lisboa, Portugal; ismaelcarvalho@edu.ulisboa.pt (I.R.); rguedes@ff.ulisboa.pt (R.C.G.)

**Keywords:** diterpenoids, abietane, carnosic acid, urea derivatives, anticancer activity, cell cycle arrest, CDK4, CDK6, reactive oxygen species (ROS)

## Abstract

A series of novel carnosic acid **1** derivatives incorporating urea moieties at the C-20 position was synthesized and evaluated for their antiproliferative activity against the HCT116 colorectal cancer cell line. Most derivatives demonstrated enhanced antiproliferative activity compared to that of carnosic acid **1**. The most promising derivatives were tested in other colorectal cancer cell lines (SW480, SW620, and Caco-2), melanoma (A375), and pancreatic cancer (MiaPaca-2). Derivative **14** consistently demonstrated the highest activity across all tested cancer cell lines, showing selectivity for cancer cells over normal cells. Further investigation of the mechanism of action in SW480 cells revealed that compound **14** induced cell cycle arrest at the G0/G1 phase by downregulating CDK4 and CDK6. Molecular docking studies revealed that compound **14** established several interactions with key residues in the active site of CDK6. Additionally, compound **14** also reduced ROS production. In summary, our results strongly indicate that compound **14** has potential as a lead compound in the development of innovative anticancer drugs.

## 1. Introduction

Cancer is a complex and heterogeneous disease that affects various organs and tissues of the body [1,2,3]. Globally, cancer is the leading cause of morbidity and mortality [4]. In 2022, approximately 20 million new cancer cases were diagnosed, resulting in 9.7 million deaths. Despite remarkable advancements in therapeutics, the primary impediments in cancer treatment are the development of resistance and side effects associated with current chemotherapeutic agents [5,6,7]. Therefore, there is an urgent need for the development of new, safer, and more effective anticancer drugs.

Natural products offer a promising source of scaffolds with a broad spectrum of bioactivity and structural diversity [8,9,10,11]. These scaffolds can be directly employed in drug discovery or as building blocks for further optimization to produce novel drugs. Diterpenoids are a diverse group of natural compounds found in various plants, marine organisms, and microorganisms [12,13,14,15,16]. In recent years, these compounds have gained significant attention owing to their wide range of potential medicinal applications, including anticancer, anti-inflammatory, immunomodulatory, antimicrobial, neuroprotective, antioxidant, antihyperlipidemic, cardioprotective, and antidiabetic effects [17,18,19,20,21,22,23]. Actually, there are some taxane-type diterpenoids (paclitaxel, docetaxel, and cabazitaxel) that are used in clinical practice for cancer treatment [24,25].

**Figure 1:**
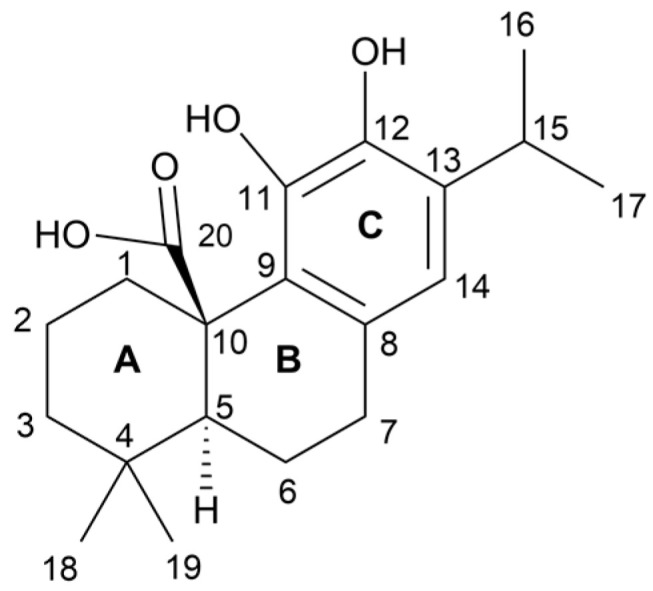
Chemical structure of carnosic acid **1**.

Accumulating evidence has shown that abietane-type diterpenoids hold significant promise as potential compounds for the discovery of anticancer drugs [26,27,28,29,30,31]. Carnosic acid (CA) **1** is an abietane-type diterpenoid present in plants belonging to the Lamiaceae family, namely, in sage (*Salvia officinalis*) and rosemary (*Rosmarinus officinalis*) plants [32,33,34]. This diterpenoid is characterized by the presence of three six-membered rings, one of which is a diphenol ring and features a carboxylic acid at the C-20 position (Figure 1). CA **1** has attracted significant scientific attention owing to its diverse range of potential biological effects, particularly its notable anticancer properties [30,32,34,35,36,37,38,39,40]. Several studies have demonstrated that CA **1** decreases cell proliferation, triggers cell cycle arrest, promotes apoptosis, and induces autophagy and endoplasmic reticulum stress in cancer cells [30,32,34,35,36]. Moreover, it exhibits the capacity to regulate inflammation, a factor closely linked to cancer development, and influences oxidative metabolism. Additionally, it plays a role in the suppression of angiogenesis and metastasis. Despite its multifaceted approach to combating cancer, there are some challenges in the application of CA **1** in the treatment of this disease. CA **1** has low aqueous solubility, limited bioavailability, and relatively modest potency [41,42,43]. To overcome these limitations, chemical modifications of the CA **1** backbone could improve its anticancer activity and pharmacokinetic properties. To the best of our knowledge, there have been limited investigations into the synthesis of CA **1** derivatives and the subsequent evaluation of their anticancer properties [44,45,46,47]. Hence, the exploration of new chemical modifications to the backbone of this diterpenoid is intriguing, with the goal of developing innovative, potent, and efficacious anticancer drugs.

**Figure 2:**
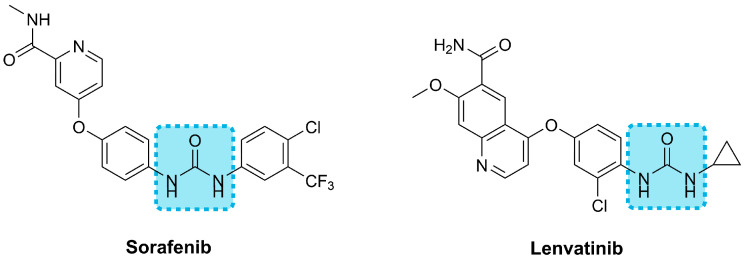
Chemical structure of urea-containing anticancer drugs used in clinical practice.

In recent years, there has been a significant surge of interest in urea-containing compounds for drug discovery [48,49,50]. Notably, some urea-based compounds such as sorafenib and lenvatinib (Figure 2) have been used in clinical practice for cancer treatment. The urea group can form hydrogen bonds with target proteins, enzymes, and receptors. The carbonyl group functions as a hydrogen bond acceptor, capable of forming two hydrogen bonds, and the two nitrogen atoms function as hydrogen bond donors, being able to donate up to four hydrogens, depending on the number of substituents [50]. Additionally, the urea group has three possible resonance structures that create a positive dipole on the nitrogen atom and a stronger negative dipole on the carbonyl group, enhancing the strength of the hydrogen bonds with biological targets. In general, this enhancement in drug–target interactions through the incorporation of the urea group improves the potency, selectivity, and pharmacokinetic properties of anticancer drugs [48,49,50]. Hence, our objective was to investigate the introduction of a urea moiety at the C-20 position of CA **1**, and subsequently assess the antiproliferative activity of the synthesized derivatives against several cancer cell lines. Among all the synthesized derivatives, compound **14** exhibited the best anticancer activity against SW480 cells, displaying selectivity for cancer cells over normal cells (BJ fibroblasts). Further investigations were conducted on this cancer cell line to gain a deeper understanding of the mechanisms underlying its anticancer properties.

## 2. Results and Discussion

### 2.1. Chemistry

**Scheme 1:**
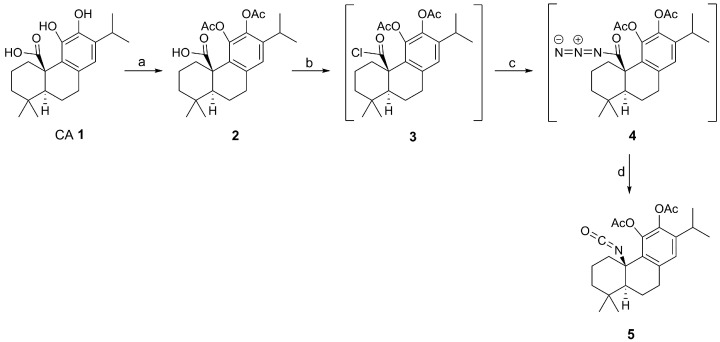
Reagents and conditions: (**a**) Acetic anhydride, DMAP, THF, R.T., anhydrous conditions, 24 h; (**b**) (COCl)_2_, CH_2_Cl_2_, DMF, 40 °C (reflux), N_2_, 5 h 30 min; (**c**) NaN_3_, H_2_O, Et_3_N, acetone, 0 °C to R.T., 22 h; (**d**) toluene, 115 °C (reflux), N_2_, 1 h.

**Scheme 2:**
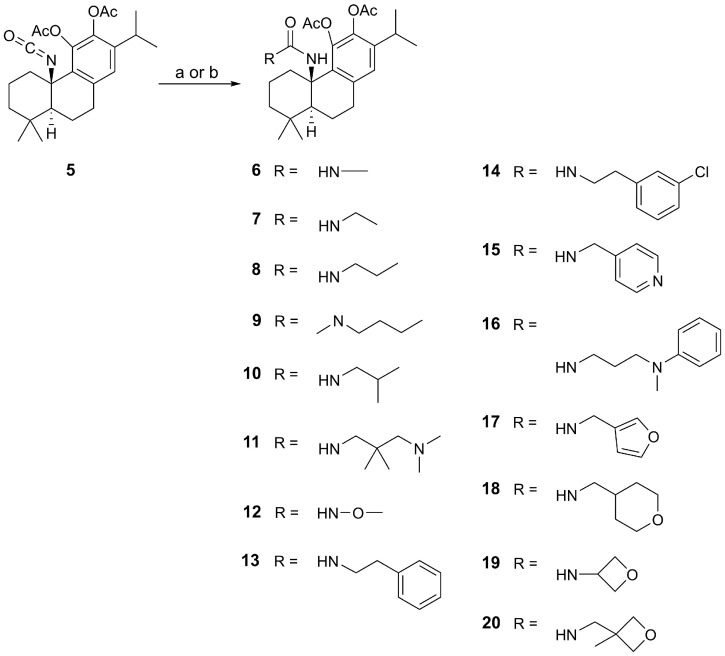
Reagents and conditions: (**a**) RNH_2_, THF, R.T., N_2_, 1 h or 3 h (derivatives **15**, **16**, and **19**); (**b**) RNH_2_, Et_3_N, THF, R.T., N_2_, 6 h (derivative **20**), or 24 h (derivative **12**).

A panel of novel CA **1** derivatives was synthesized, as outlined in Scheme 1 and Scheme 2. Structural elucidation and assessment of the purity of all compounds were performed using a combination of analytical techniques including nuclear magnetic resonance (NMR), infrared (IR) spectroscopy, mass spectrometry (MS), melting point (MP), and elemental analysis. The structural elucidation of the compounds was conducted using 1D NMR techniques, including ^1^H, and ^13^C, as well as 2D NMR methods, such as COSY, NOESY, HSQC, and HMBC, for complete characterization.

CA **1** contains two highly reactive phenolic hydroxyl groups [46,51]. Thus, these positions were modified by the introduction of acetate groups (derivative **2**) to minimize the formation of secondary products in subsequent reactions (Scheme 1). The derivative **2** was produced in almost quantitative yield using acetic anhydride and 4-dimethylaminopyridine (DMAP) in THF at room temperature, according to previous studies reported in the literature [44,46,51].

In the subsequent phase of our research, we focused on transforming the carboxylic acid at the C-20 position into an isocyanate moiety. This transformation was achieved through a series of chemical reactions (Scheme 1) [52,53]. Initially, derivative **2** was reacted with oxalyl chloride in the presence of a catalytic amount of DMF in dichloromethane under reflux (40 °C). This reaction resulted in the formation of a diacetate compound containing an acyl chloride functional group, referred to as intermediate **3**. Subsequently, intermediate **3** was dissolved in an aqueous sodium azide solution, and the reaction occurred in acetone and was catalyzed by Et_3_N, leading to the formation of acyl azide (intermediate **4**). The acyl azide of intermediate **4** underwent a Curtius rearrangement in toluene under reflux, affording a derivative containing an isocyanate functional group (derivative **5**). The successful incorporation of the isocyanate group was confirmed by the observation of a peak at 123.40 ppm in the ^13^C NMR spectrum, which corresponds to the quaternary carbon bonded to both nitrogen and oxygen. Additionally, the IR spectrum exhibited a broad and intense absorption band at 2247 cm^−1^, indicative of the stretching vibration of the isocyanate group.

Recently, it has been demonstrated that incorporating a urea moiety into natural compounds is crucial for enhancing their anticancer properties [48,49,50,53]. Thus, the isocyanate derivative (**5**) was reacted with a variety of amines in THF at room temperature to produce a series of urea-containing derivatives (**6**–**20**, Scheme 2) with yields ranging from 48 to 83%. In the derivatives resulting from the reaction with a primary amine (all except derivative **9**), the presence of a urea moiety was confirmed by the observation of two broad singlets in the ^1^H NMR spectra, corresponding to the protons of the -NH groups. Derivative **9**, synthesized from the secondary amine N-butylmethylamine, exhibited only one broad singlet corresponding to the proton of its single -NH group. In the ^13^C NMR spectra, a peak characteristic of the urea carbonyl group was observed within the range of 160.93–155.70 ppm. The IR spectra feature a strong band in the 1681–1600 cm^−1^ range, which is indicative of the stretching vibration of the carbonyl group of urea. Additionally, one or two bands appeared in the 3469–3248 cm^−1^ range, indicative of the stretching vibration of the N–H bond, while a strong band appeared in the 1580–1521 cm^−1^ range, reflecting a combination of a C–N stretching band and an N–H bending band. Collectively, these results supported the synthesis of CA **1** derivatives containing urea functional groups.

### 2.2. Biology

#### 2.2.1. In Vitro Antiproliferative Activity

The antiproliferative activity of CA **1** and its derivatives was evaluated against a colorectal cancer (CRC) cell line (HCT116) using the MTT assay. The anticancer drug cisplatin was used as the reference drug. The results are expressed as IC_50_ values measured after 72 h of treatment, representing the compound concentration required to inhibit 50% of the cell growth, and are presented in Table 1.

**Figure 3:**
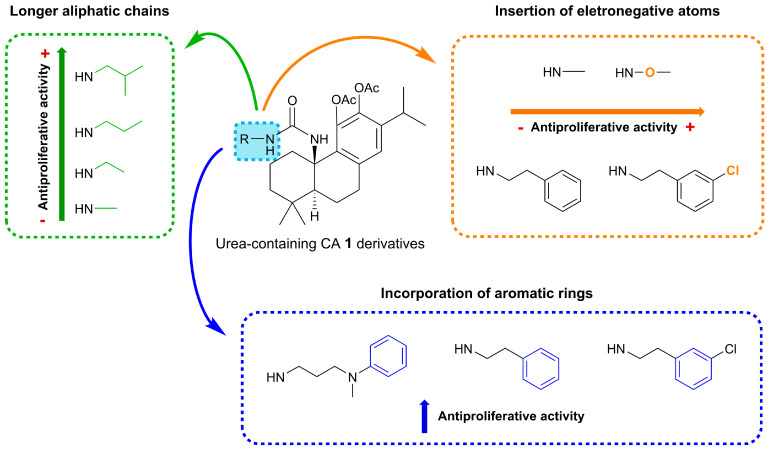
Schematic illustration of the SAR of urea-containing CA **1** derivatives based on their antiproliferative activity against HCT116 cells.

As shown in Table 1, the substitution of phenolic hydroxyl groups with acetate groups (compound **2**) did not interfere with the antiproliferative activity, maintaining potency nearly identical to that of CA **1**. This result suggests that the hydroxyl groups do not play a pivotal role in antiproliferative activity. However, the incorporation of an isocyanate group (**5**) enhanced the antiproliferative activity, with an IC_50_ value of 28 µM. In general, the incorporation of a urea group (**8**–**17**) resulted in enhanced antiproliferative activity compared with that of the hit compound CA **1**. Among the aliphatic urea-containing derivatives (compounds **6**–**12**), it was observed that longer aliphatic chains positively influenced antiproliferative activity, leading to lower IC_50_ values. Furthermore, the inclusion of electronegative atoms demonstrated a favorable effect on the antiproliferative activity. This was evident in the comparison between the IC_50_ values of compounds **6** (IC_50_ = 55 µM) and **12** (IC_50_ = 41 µM), which only differed in the presence of an oxygen atom on the urea moiety. Among the aromatic urea-based derivatives (compounds **13**–**17**), the compounds **13**, **14**, and **16** displayed the most potent antiproliferative activity, with IC_50_ values of 14, 9.8, and 12.0 µM, respectively. Notably, these compounds demonstrated better antiproliferative activity against HCT116 cells than cisplatin. The key distinction between derivatives **13** and **14** is the presence of a halogenated atom (-Cl) at the meta-position of the benzene ring. This chemical difference was responsible for the enhanced activity observed for compound **14**. Compounds containing a tetrahydropyran moiety (**18**) and oxetane moieties (**19** and **20**) exhibited weaker antiproliferative activities than CA **1**. The main structure-activity relationship (SAR) conclusions established from the IC_50_ of urea-containing CA **1** derivatives in HCT116 cells are summarized in Figure 3.

Cell lines of the same cancer type can have different genetic mutations that can significantly affect their response to treatment [54,55]. Some mutations may sensitize cancer cells to certain therapies, whereas others can confer resistance to treatment. Given these considerations, we chose other CRC cell lines (SW480, SW620, and Caco-2) to assess the antiproliferative activity of compounds **13**, **14**, and **16**, that were the ones with the highest antiproliferative activity against the HCT116 cell line, a wild-type p53 and microsatellite instable CRC cell line [54]. On the other hand, SW480, SW620, and Caco-2 cells have mutations in the p53 gene, resulting in altered p53 proteins, and are microsatellite stable CRC cell lines. As shown in Table 2, compounds **13**, **14**, and **16** exhibited higher potency than the parent compound (CA **1**) in SW480, SW620, and Caco-2 cells. Moreover, these derivatives exhibited lower IC_50_ values in these cell lines than in HCT116 cells, indicating that they are more potent in CRC cells with p53 mutations. Mutant p53 proteins acquire oncogenic properties that drive cancer progression [56]. Thus, reducing mutant p53 levels may be an effective approach for targeting cancers with p53 alterations. Our research group previously showed that a celastrol derivative with the same urea substituent as compound **14** reduced mutant p53 protein levels in SKOV-3 cells [53]. Compound **14** may act through a similar mechanism, potentially explaining its enhanced potency in CRC cell lines with p53 mutations. Given the structural similarity of the urea substituents in compounds **13** and **16** to those in compound **14**, a similar mechanism may be inferred for these compounds as well.

**Table 1:** Cell viability (IC_50_) of CA **1**, its derivatives, and cisplatin against HCT116 colorectal cancer cell line.

Compound	Cell Line/IC_50_ (µM) ^1^
	HCT116
CA **1**	42 ± 4
**2**	44 ± 4
**5**	28 ± 3
**6**	55 ± 5
**7**	44 ± 4
**8**	31 ± 1
**9**	22 ± 1
**10**	22 ± 2
**11**	30 ± 1
**12**	41 ± 3
**13**	14 ± 2
**14**	9.8 ± 0.5
**15**	33 ± 2
**16**	12.0 ± 0.5
**17**	21 ± 2
**18**	51 ± 5
**19**	>60
**20**	46 ± 2
**Cisplatin**	21 ± 1 [57] ^2^

IC_50_ values were determined using the MTT assay after 72 h of incubation with different concentrations of each compound. Values are presented as mean ± SD of at least three independent experiments. ^1^ IC_50_ is the concentration of the compound required to inhibit 50% of cell growth. ^2^ IC_50_ value obtained previously by our research group using the same methodology.

**Figure 4:**
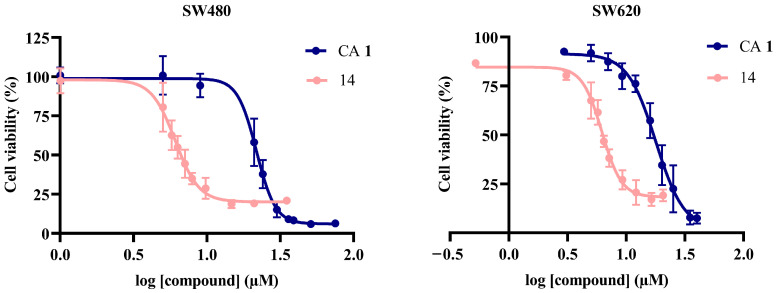
Dose-dependent effects of CA **1** and its derivative **14** on SW480 and SW620 cell viability after 72 h of treatment. Cell viability was assessed using the MTT assay. Data are presented as mean ± SD of at least three independent experiments.

Among the three p53-mutated cell lines, the most promising antiproliferative effects were observed in SW480 and SW620 cells. These two cell lines are related because the SW620 cell line is derived from a lymph node metastasis of the same tumor that originated in the SW480 cell line. Notably, compound **14** displayed the most significant antiproliferative activity with an IC_50_ of 6.1 µM in SW480 and SW620 cells (Table 2 and Figure 4). These results for compound **14** are noteworthy because they showed similar efficacies against both primary and metastatic cancer cell lines. This achievement is significant considering the well-established challenges associated with treating metastatic cancer, which often involves the development of treatment resistance [58].

**Table 2:** Cell viability (IC_50_) of CA **1**, derivatives **13**, **14**, and **16**, and cisplatin against cancer cell lines (colorectal, melanoma, and pancreas) and a normal fibroblast cell line (BJ).

Compound	Cell Line/IC_50_ (µM) ^1^					
	Colorectal			Melanoma	Pancreas	Non-Tumoral
	SW480	SW620	Caco-2	A375	MiaPaca-2	BJ
CA **1**	21.8 ± 0.7	18 ± 2	34 ± 2	27.6 ± 0.5	21 ± 2	N.D.
**13**	8.6 ± 0.3	9 ± 1	10.9 ± 0.6	N.D.	N.D.	N.D.
**14**	6.1 ± 0.3	6.1 ± 0.3	8.0 ± 0.5	7.0 ± 0.4	9.0 ± 0.8	>25
**16**	7.6 ± 0.6	6.4 ± 0.4	9.6 ± 0.6	7.6 ± 0.6	9.3 ± 0.4	N.D.
**Cisplatin**	15.2 ± 0.4 [59] ^2^	1.4 ± 0.5 [60] ^3^	12.5 ± 0.9 [61] ^2^	3 ± 1 [62] ^2^	5 ± 1 [63] ^2^	10 ± 2 [64] ^3^

IC_50_ values were determined using the MTT assay after 72 h of incubation with increasing concentrations of each compound. Values are presented as the mean ± SD of at least three independent experiments. N.D.: not determined. ^1^ IC_50_ is the concentration of the compound required to inhibit 50% of the cell growth. ^2^ IC_50_ value acquired from literature using the same methodology. ^3^ IC_50_ value obtained previously by our research group using the same methodology.

As compounds **14** and **16** were the most potent compounds, their antiproliferative potential was also assessed in melanoma (A375) and pancreatic (MiaPaca-2) cancer cell lines (Table 2). These two derivatives were more potent than the parent compound CA **1** and showed a higher antiproliferative effect in melanoma cells. Given that compound **14** displayed the highest potency across all evaluated cancer cell lines, the selectivity of this derivative for cancer cells was evaluated using the non-tumoral fibroblast cell line (BJ) as a reference. As shown in Table 2, compound **14** was less potent in inhibiting the growth of BJ fibroblasts than that of cancer cells, demonstrating its selectivity for cancer cells. Considering these favorable results, compound **14** was selected for further studies to elucidate its anticancer mechanism. Among the four assayed CRC cell lines, we chose SW480 because compound **14** had the lowest IC_50_, showing an even better IC_50_ than cisplatin, and because microsatellite stable cell lines are closer to the type of CRC tumor that will be treated with chemotherapy.

#### 2.2.2. Cell Growth and Doubling Time of SW480 Cells

**Figure 5:**
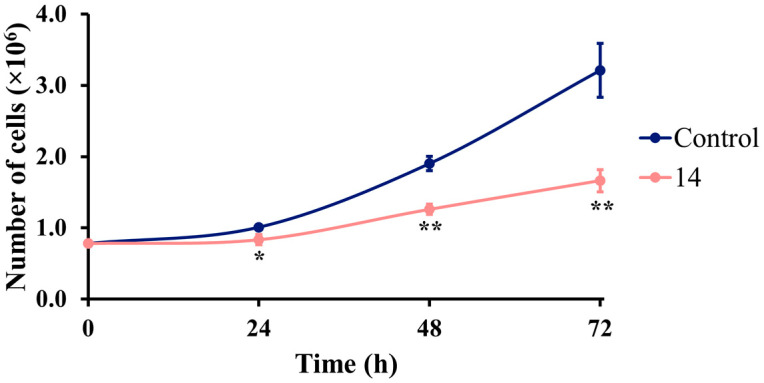
Growth curves of SW480 cells untreated (control) and cells treated with compound **14** (IC_50_ concentration) for 24, 48, and 72 h. After each time point, the cells were collected, and the number of viable cells was counted. Results are presented as the mean ± SD of three independent experiments. Differences between the control and treated groups were evaluated using multiple *t*-tests (one independent *t*-test per row), with statistical significance considered at *p* < 0.05 (*) and *p* < 0.01 (**).

The growth of SW480 cells treated with compound **14** for 24, 48, and 72 h was evaluated to determine the effect of **14** on both cell growth pattern and cell doubling time. As shown in Figure 5, SW480 cells exposed to this treatment exhibited a slower growth pattern over time. The cell doubling time was determined by dividing the natural logarithm of 2 by the growth rate [65]. As expected, the cell doubling time increased from 42 h in the control group to 70 h in SW480 cells treated with compound **14**. A longer doubling time indicates a slower rate of cell growth and division. These findings confirmed that compound **14** influences the growth of SW480 cells and decelerates their proliferation.

#### 2.2.3. Analysis of Cell Apoptosis

Apoptosis is a type of programmed cell death that is crucial for maintaining cellular homeostasis [66,67]. Various conditions can trigger activation of this biological pathway, such as DNA damage or uncontrolled cell proliferation. Dysregulation of apoptosis contributes to the development of various diseases such as cancer.

Apoptosis can be detected by staining the cells with annexin V and propidium iodide (PI). This assay is based on the principle that normal cells possess lipid phosphatidylserine (PS) on the inner side of the cell membrane [68]. However, during the early stages of apoptosis, the membrane flips and PS is exposed on the outer side of the cell membrane. Thus, annexin V fluorescently labeled with fluorescein isothiocyanate (FITC) binds with high affinity for PS. In late apoptosis/necrosis, the cells experience a ruptured membrane, allowing Annexin V to interact with the entire cell membrane. At this stage, the nuclear membrane loses its integrity, which facilitates the binding of PI to DNA. Thus, this dual-staining protocol allows for the detection and quantification of live cells (annexin V−, PI−), early apoptotic cells (annexin V+, PI−), and late apoptotic/necrotic cells (annexin V+, PI+) [68].

**Figure 6:**
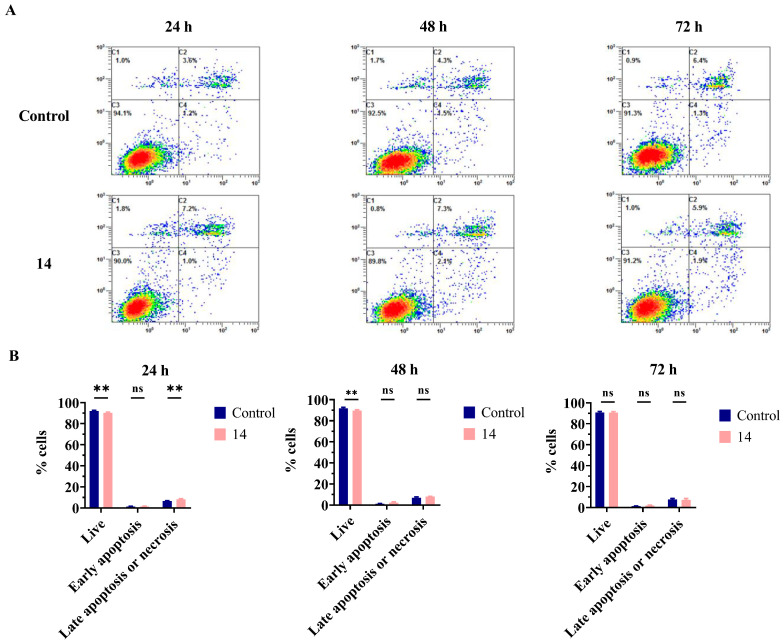
Apoptosis analysis at 24, 48, and 72 h in SW480 cells untreated (control) or treated with compound **14** at its IC_50_ concentration. After each time point, the cells were stained with annexin V-FITC/PI and subsequently examined by flow cytometry. (**A**) Representative histograms showing the variation in the percentage of live cells (lower left quadrant, C3), cells in early apoptosis (lower right quadrant, C4), and cells in late apoptosis/necrosis (upper left (C1) and right (C2) quadrants). (**B**) Graph bar summarizes the variation in the percentage of live cells, cells in early apoptosis, and cells in late apoptosis/necrosis. The results are expressed as the mean ± SD of three independent experiments. Differences between the control and treated groups were evaluated using multiple *t*-tests (one independent *t*-test per row), considering statistical significance at *p* < 0.01 (**). ns, statistically non-significant.

SW480 cells were treated with compound **14** at its IC_50_ concentration for 24, 48, and 72 h. Subsequently, the cells were stained with annexin V-FITC/PI before fluorescence-activated cell sorting (FACS) analysis. The percentage of cells in late apoptosis/necrosis increased slightly from 6.5% in control cells to 8.3% in cells treated with compound **14** at 24 h. However, this effect was not sustained after 48 and 72 h of exposure to **14** (Figure 6). These findings suggested that apoptosis does not play a pivotal role in the anticancer mechanism of compound **14** in SW480 cells.

#### 2.2.4. Analysis of Cell Cycle and Related Proteins

The cell cycle constitutes a series of ordered events that culminate in cell growth and division [69,70]. Dysregulation of the cell cycle can lead to uncontrolled cell division and accumulation of genetic mutations, resulting in the development of cancer. Thus, the development of anticancer drugs aimed at regulating the cell cycle represents a promising approach for chemotherapy.

**Figure 7:**
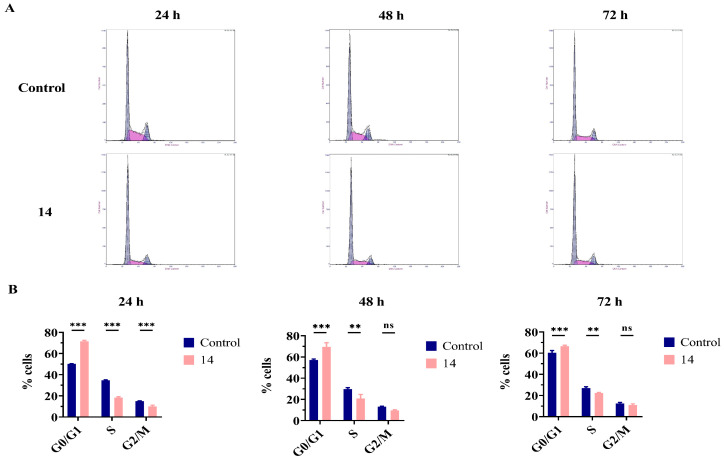
Cell cycle analysis of SW480 cells untreated (control) or cells treated with compound **14** at IC_50_ concentration for 24, 48, and 72 h. After, cells were fixed in ethanol and stained with PI, and the DNA content was analyzed by flow cytometry. (**A**) Representative histograms showing the variation in the number of cells in each phase of the cell cycle (G0/G1, S, and G2/M). (**B**) Graph bar summarizes the variation in the percentage of cells in the G0/G1, S, and G2/M phases of three independent experiments. The results are expressed as the mean ± SD. Differences between the control and treated groups were evaluated by two-way ANOVA (Sidak’s post hoc comparison), considering statistical significance at *p* < 0.01 (**) and *p* < 0.001 (***). ns, statistically non-significant.

As the induction of apoptosis by compound **14** did not appear to play a significant role in its anticancer mechanism in SW480 cells and this compound increased the duplication time of treated cells, we wondered whether cell cycle arrest was the primary mechanism behind the antiproliferative activity of compound **14**. To test this hypothesis, SW480 cells were treated with compound **14** at its IC_50_ concentration for 24, 48, and 72 h. At each time point, the cells were fixed and permeabilized with ethanol, stained with PI, and analyzed by FACS. As shown in Figure 7, compound **14** consistently arrested the cell cycle at the G0/G1 phase at all the tested time points. At the three time points tested, the percentage of cells in the G0/G1 phase was higher than 66.6%, with the highest difference between the treated and control cells at 24 h, where the percentage of cells in the G0/G1 phase significantly increased from 50.3% in the control group to 71% in the SW480 cells treated with compound **14**. These findings strongly suggest that the antiproliferative activity of compound **14** is closely associated with cell cycle arrest at the G0/G1 phase. Previous studies have reported that CA **1** induces cell cycle arrest at the G0/G1 phase in different cancer cell lines with mutations in the p53 gene, such as colon (HT-29), melanoma (B16F10), liver (MHCC97-H and Bel7402), and breast (MDA-MB-453) cancer cell lines [71,72,73,74]. Thus, compound **14** exhibited the same effect on the cell cycle in a p53-mutated cell line as its parent compound.

**Figure 8:**
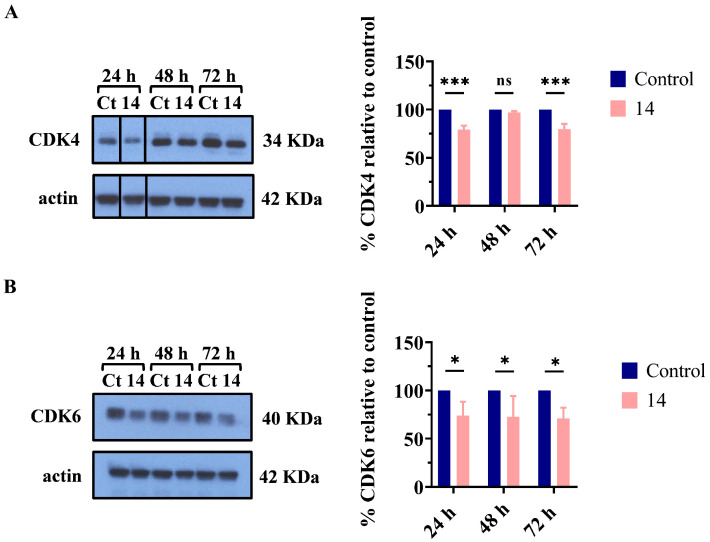
Expression levels of (**A**) CDK4 and (**B**) CDK6 in SW480 cells untreated (control) or cells treated with compound **14** at its IC_50_ concentration for 24, 48, and 72 h. The levels of the indicated proteins were analyzed by Western blot. Actin was used as loading control. The vertical black lines in the blot images indicate the locations where the images were spliced to rearrange the order of the lanes. Graph bars depict the variation in the percentage of protein expression levels. The results are presented as mean ± SD of three independent experiments, except for CDK4 at 24 and 48 h (*n* = 2). Differences between the control and treated groups were evaluated using multiple *t*-tests (one independent *t*-test per row), considering statistical significance at *p* < 0.05 (*) and *p* < 0.001 (***). ns, statistically non-significant.

The distinct phases of the cell cycle require specific cyclin-dependent kinase (CDK)–cyclin complexes to regulate the progression and transition between phases [69]. During the G1 phase, the CDK4/6–cyclin D complex plays a pivotal role in promoting cell cycle progression. Some studies have reported that a decrease in CDK4/CDK6 levels leads to cell cycle arrest at the G0/G1 phase [75,76,77,78]. Thus, we investigated the effect of compound **14** on CDK4 and CDK6 expression over time (24, 48, and 72 h). As depicted in Figure 8, treatment of SW480 cells with compound **14** resulted in the downregulation of CDK4 at 24 and 72 h, as well as CDK6 at all three time points (24, 48, and 72 h), in comparison with untreated cells.

In summary, our results indicate that compound **14** exerts its antiproliferative action by interfering with CDK4 and CDK6 expression, thereby arresting the cell cycle in the G0/G1 phase. Further research is necessary to determine the exact mechanism by which this compound exerts this effect.

#### 2.2.5. Molecular Docking Studies

The enzymatic activity of CDK4/CDK6 is crucial for cell cycle progression from the G1 to S phase [79]. Therefore, inhibition of CDK4/CDK6 represents a promising approach for cancer treatment by blocking the G1 to S phase transition and reducing cell proliferation. Currently, there are three CDK4/CDK6 inhibitors approved by the FDA (palbociclib, ribociclib, and abemaciclib) that are used for the treatment of breast cancer. In recent years, there has been an increasing interest in the development of CDK4/CDK6 inhibitors for various cancer types [79]. Therefore, we investigated whether compound **14** could interact directly with CDK6.

**Figure 9:**
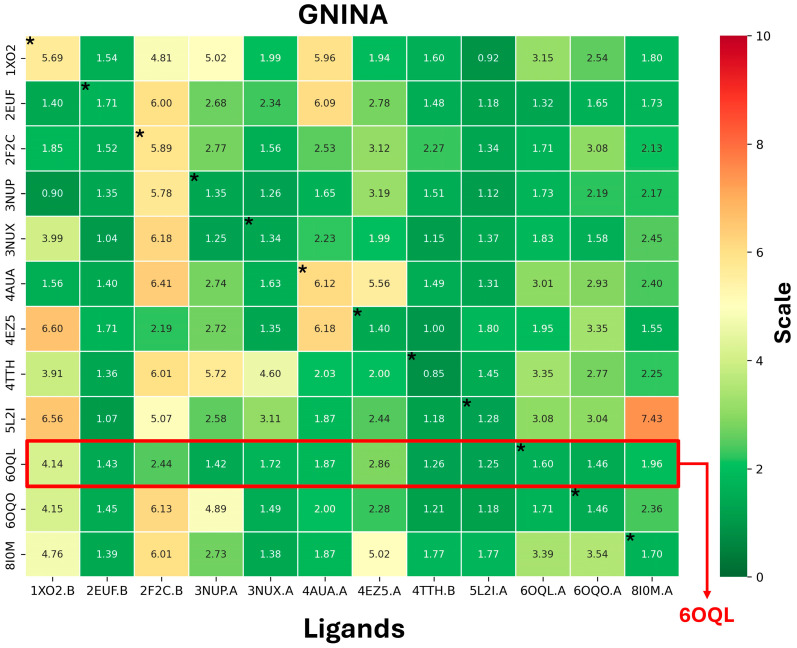
Validation of molecular docking protocol and identification of the best CDK6 structure. Results of self-docking (diagonal entries) and cross-docking for 12 PDB structures of CDK6, along with respective RMSDs using GNINA 1.0 docking software. Colors representation: RMSD < 2 Å: dark green; 2 Å ≤ RMSD < 4 Å: light green; 4 Å ≤ RMSD < 6 Å: yellow; 6 Å ≤ RMSD < 8 Å: yellow-to-orange gradient; RMSD > 8 Å: red. * denotes values derived from self-docking calculations.

Molecular docking studies were performed to predict the binding mode and interactions between compound **14** and the CDK6 binding site. The docking protocol was first validated by self-docking with reproduction of the binding pose of the co-crystallized ligand in the 6OQL active site with an RMSD value of 1.60 Å as well as by cross-docking of each crystallographic ligand across 12 crystal structures of CDK6. The validation results showed that the PDB ID 6OQL crystal structure and GNINA 1.0 software were the best combination (Figure 9).

**Figure 10:**
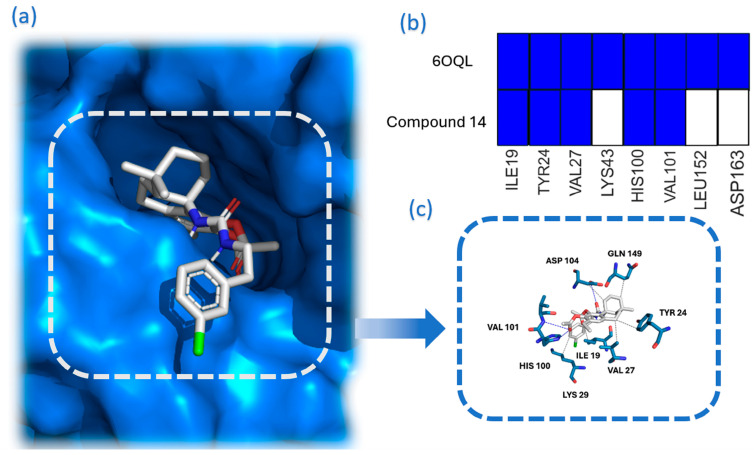
The binding mode of compound **14** into the binding pocket of CDK6 (PDB ID: 6OQL), predicted by the GNINA 1.0 docking software. (**a**) Best docking pose of compound **14**; (**b**) ligand–protein interactions of the 6OQL crystal structure compared to those established by compound **14** (five out of eight interactions are with the same amino acid residues); (**c**) ligand–protein interactions between compound **14** and CDK6 binding site residues.

Docking studies indicated that compound **14** and the ligand in the crystallographic complex occupied the same region within the CDK6 structure (PDB ID: 6OQL). Compound **14** was positioned at the entrance of the CDK6 binding pocket (Figure 10a) and established interactions with ILE19, TYR24, VAL27, LYS29, HIS100, VAL101, ASP104, and GLN149 amino acid residues (Figure 10c). The interactions established with ILE19, TYR24, VAL27, HIS100, and VAL101 residues were also verified in the crystallographic complex (Figure 10b). Additionally, compound **14** formed two hydrophobic interactions with LYS29 and GLU149, contributing to the further stabilization of compound **14**.

Concluding, the results revealed that compound **14** can establish several interactions with important active site residues of CDK6. Moreover, compound **14** shares interactions with the same five amino acid residues as the crystalized ligand, which is a potent inhibitor of this kinase (Ki = 4.9 nM) [80]. These findings suggest that compound **14** could be a potential inhibitor of CDK6, contributing to the reduction in cell proliferation of SW480 cells.

#### 2.2.6. Analysis of ROS Generation and Related Proteins

ROS are highly reactive molecules generated as byproducts of cellular metabolism and serve as important signaling molecules in cell regulation and immune responses [81,82,83]. Disruption of the balance of ROS levels can contribute to the initiation and progression of some diseases, such as cancer. In cancer, ROS plays a dichotomous role. A modest increase in ROS levels plays a pivotal role in tumorigenesis and cancer development. However, a significant increase in ROS levels can be cytotoxic, potentially inducing anti-tumorigenic effects via oxidative damage and ROS-mediated cell death. Conversely, a decrease in ROS levels inhibits pro-tumorigenic signals, leading to reduced cell proliferation and survival, fewer metabolic adaptations, and decreased genetic instability and DNA damage [83,84,85,86]. Considering the role of ROS in cancer, modulating ROS levels holds promise as an effective strategy for combating this disease [81,83,84,85,86].

**Figure 11:**
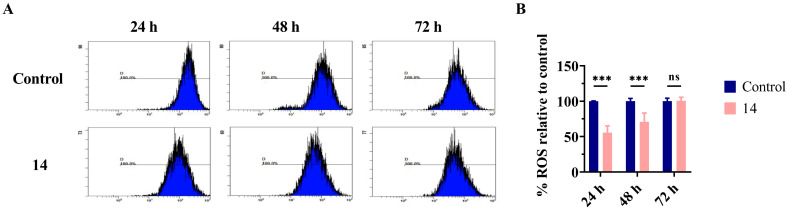
Intracellular ROS levels in SW480 cells untreated (control) or treated with compound **14** (IC_50_ concentration) for 24, 48, and 72 h. After each time point, the cells were incubated with the H_2_DCFDA probe and stained with PI, and the intensity of fluorescence emitted by DCF was quantified by flow cytometry. (**A**) Representative histograms showing the magnitude of the DCF fluorescent probe intensity proportional to ROS activity. (**B**) Graph bar summarizing variations in the percentage of intracellular ROS levels. Data are expressed as the mean ± SD of three independent experiments. Differences between the control and treated groups were evaluated using multiple *t*-tests (one independent *t*-test per row), considering statistical significance at *p* < 0.001 (***). ns, statistically non-significant.

Various methods have been employed to measure intracellular ROS, with one common approach being fluorescence-based techniques that involve probes [87]. In our ROS generation assay, we incubated untreated cells and cells treated with compound **14** with a 2′,7′-dichlorodihydrofluorescein diacetate (H_2_DCFDA) probe. As depicted in Figure 11, at 24 and 48 h, the percentage of intracellular ROS significantly decreased to 56% and 71%, respectively, in SW480 cells treated with compound **14** compared with the control group. However, no significant changes in the ROS levels were observed at 72 h. Periodic oscillations in the cellular redox environment, known as the redox cycle, regulate cell cycle progression [88,89,90]. Cells encounter a more oxidizing redox environment as the cycle progresses. In fact, intracellular ROS levels may play a crucial role in the decision-making process for cells to transition from the G1 to S phase. Some studies have observed a decrease in ROS levels accompanied by cell arrest in the G0/G1 phase, similar to our observations with compound **14** [91,92,93].

In conclusion, our results indicate that compound **14** plays an antioxidant role in SW480 cells. In recent years, several studies have explored the potential of molecules with antioxidant properties for cancer treatment [85,94,95,96,97,98]. This approach has gained prominence because of its effectiveness in mitigating oxidative stress, which is a key contributor to cancer development and progression. The reduction in oxidative stress is beneficial for minimizing the adverse effects of oxidative stress in normal cells, reducing the side effects often associated with anticancer drugs that induce ROS production [96,99,100].

**Figure 12:**
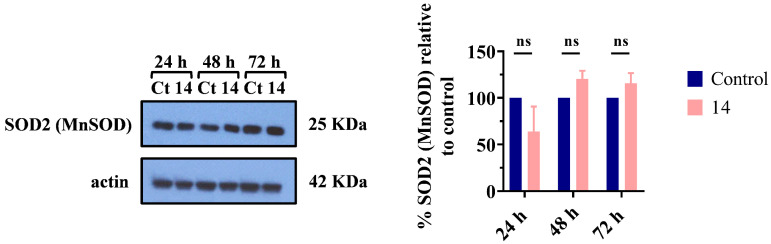
SOD2 (MnSOD) expression levels in SW480 cells untreated (control) or treated with compound **14** at IC_50_ concentration for 24, 48, and 72 h. Western blot was performed to evaluate the levels of the indicated proteins. Actin was used as loading control. Graph bars depict the variation in the percentage of protein expression levels. The results are expressed as mean ± SD of at least two independent experiments (24 and 48 h, *n* = 2; 72 h, *n* = 3). Differences between the control and treated groups were evaluated using multiple *t*-tests (one independent *t*-test per row). ns, statistically non-significant.

Detoxification of ROS can be accomplished by enzymatic antioxidants, which are responsible for neutralizing various types of ROS [83,85]. Superoxide dismutases (SODs) are metalloenzymes present in different cellular compartments that use different metal ions as cofactors [83,85,101]. For example, SOD1 and SOD3 are present in the mitochondrial intermembrane space/cytosol and extracellular space, respectively, using copper (Cu^2+^) and zinc (Zn^2+^) as cofactors. SOD2 (MnSOD) uses manganese (Mn^2+^) as a cofactor and is located in the mitochondrial matrix and the inner mitochondrial membrane. Mitochondria are the major producers of intracellular ROS, and most superoxide anions generated within the mitochondria are efficiently dismutated by SOD2 (MnSOD). To verify whether SOD2 (MnSOD) contributed to the observed reduction in ROS levels, we evaluated the expression of this enzyme in SW480 cells treated with compound **14** for 24, 48, and 72 h. As shown in Figure 12, there were no significant changes in the expression of SOD2 (MnSOD) at the three time points tested. Based on these findings, we conclude that the antioxidant effect of compound **14** does not arise from the direct scavenging of intracellular ROS through SOD2 (MnSOD). Therefore, it is possible that the antioxidant effect is attributable to the regulation of other antioxidant enzymes, non-enzymatic antioxidant systems, or an influence on ROS production [85,95,96,97,98,102]. Further investigations are required to understand this effect.

## 3. Materials and Methods

### 3.1. Chemistry

#### 3.1.1. General

The compounds (3-methyloxetan-3-yl) methanamine hydrohloride (EN300-75686), oxetan-3-amine (EN300-49064), (oxan-4-yl) methanamine (EN300-57193), 1-(pyridin-4-yl) methanamine (EN300-19585), and 1-(furan-3-yl) methanamine (EN300-53867) were obtained from Enamine (Kyiv, Ukraine). CA **1** and the remaining amines were obtained from Sigma-Aldrich (St. Louis, MO, USA). Reagents and solvents were purchased from Sigma-Aldrich (St. Louis, MO, USA) and VWR Portugal (Carnaxide, Portugal) and were subjected to standard drying procedures. Thin-layer chromatography (TLC) was performed on aluminum TLC plates coated with silica gel 60 and the fluorescent indicator F254 (Kieselgel 60 HF254) obtained from Merck Co. (Rahway, NJ, USA). Preparative TLCs were prepared using glass plates coated with a mixture of silica gel 60 (Kieselgel 60G) and silica gel 60 F254 (Kieselgel 60HF254) obtained from Merck Co. (Rahway, NJ, USA). Mp was determined using a Büchi^®^ Mp B-540 apparatus (Büchi^®^, Flawil, Switzerland) and reported without correction. IR spectra were acquired using a PerkinElmer Spectrum 400 FT-IR/FT-NIR spectrometer (PerkinElmer, Waltham, MA, USA). NMR was used to elucidate chemical structures based on 1D NMR (^1^H and ^13^C) and 2D NMR (COSY, NOESY, HSQC, and HMBC). NMR spectra were recorded on a Bruker Avance III 400 MHz spectrometer (Bruker, Billerica, MA, USA) in CDCl_3_, operating at 400 MHz for ^1^H and 100 MHz for ^13^C. Chemical shifts (δ) are reported in parts per million (ppm), and coupling constants (J) are expressed in hertz (Hz). Spectra were calibrated at δ 7.26 ppm (^1^H) and δ 77.16 ppm (^13^C) to residual solvent signals (CDCl_3_). MS was performed using a Thermo Scientific Finnigan LXQ Linear Ion Trap Mass Spectrometer (Thermo Fisher Scientific, Waltham, MA, USA) with electrospray ionization (ESI). The ESI conditions were as follows: 5 kV in positive mode, a capillary temperature of 250 °C, and a sheath gas flow of 5 U. Elemental analysis was performed using a TruSpec 630-200-200 CHNS analyzer (Leco Corporation, St. Joseph, MI, USA). Compounds were named following IUPAC rules as applied by ChemDraw Professional 22.0.0.22 (PerkinElmer, Shelton, CT, USA).

#### 3.1.2. (4a*R*,10a*S*)-5,6-Diacetoxy-7-isopropyl-1,1-dimethyl-1,3,4,9,10,10a-hexahydrophenanthrene-4a(*2H*)-carboxylic acid (**2**)

To a stirred solution of CA **1** (150.0 mg, 0.45 mmol) in dry THF (3.5 mL) were added acetic anhydride (0.25 mL, 2.64 mmol) and DMAP (22.5 mg, 0.18 mmol). The mixture was stirred for 24 h at room temperature under an anhydrous atmosphere. Subsequently, the reaction mixture was evaporated under reduced pressure to remove THF and extracted with ethyl acetate (3 × 30 mL) from water (15 mL). The organic phase obtained was sequentially washed with 5% aqueous HCl (45 mL), 10% aqueous NaHCO_3_ (45 mL), water (2 × 45 mL), and brine (45 mL). The organic layer was dried over anhydrous Na_2_SO_4_, filtered, and concentrated under reduced pressure to obtain **2** as a white powder (177.2 mg, 94%). Mp: 220.4–222.3 °C. IR (neat) _vmax_: 3022, 1777, 1768, 1689, 1194, 1186, 1166 cm^−1^. ^1^H NMR (400 MHz, CDCl_3_) δ 6.95 (1H, s, 14-H), 3.22 (1H, d, *J* = 13.4 Hz), 2.98–2.79 (3H, m), 2.38–2.27 (1H, m), 2.26 (3H, s, OCOCH_3_), 2.24 (3H, s, OCOCH_3_), 2.13–2.07 (1H, m), 1.88–1.80 (1H, m), 1.57–1.44 (3H, m), 1.32–1.23 (2H, m), 1.21 (3H, d, *J* = 6.9 Hz, CHCH_3_), 1.14 (3H, d, *J* = 6.9 Hz, CHCH_3_), 0.96 (3H, s, CH_3_), 0.86 (3H, s, CH_3_); ^13^C NMR (100 MHz, CDCl_3_) δ 179.61 (COOH), 168.80 (OCOCH3), 168.33 (OCOCH3), 141.49, 140.07, 138.82, 136.93, 132.09, 125.36 (C14), 53.91, 47.70, 41.21, 34.66, 34.17, 32.62, 32.05, 27.52, 23.12, 22.87, 20.68, 20.56, 20.20, 19.96, 18.26. ESI-MS m/z: 439.3 [M + Na]^+^. Anal. Calcd. For C_24_H_32_O_6_·0.3C_6_H_14_: C, 70.05; H, 8.25. Found: C, 70.52; H, 7.90%.

#### 3.1.3. (4b*R*, 8a*S*)-4b-Isocyanato-2-isopropyl-8,8-dimethyl-4b,5,6,7,8,8a,9,10-octahydrophenanthrene-3,4-diyl diacetate (**5**)

To a solution of **2** (300.0 mg, 0.72 mmol) in dry CH_2_Cl_2_ (8 mL) was added slowly oxalyl chloride (0.16 mL, 1.9 mmol) and dry DMF (0.15 mL, 0.72 mmol). The mixture was stirred for 5 h 30 min. at 40 °C (reflux) under nitrogen atmosphere. The mixture was concentrated to dryness under reduced pressure to obtain crude **3**. The resulting residue (**3**) was dissolved in acetone (8 mL) and transferred dropwise to a stirred aqueous solution (1.8 mL) of sodium azide (176.5 mg, 2.71 mmol) at 0 °C. Et_3_N (1.5 mmol, 0.21 mL) was added to the mixture, which was maintained for 22 h at room temperature under agitation. The reaction mixture was extracted with ethyl acetate (3 × 15 mL) from water (7.5 mL). The organic phase was washed with brine (22.5 mL), dried over anhydrous Na_2_SO_4_, filtered, and concentrated under reduced pressure to obtain crude **4**. The resulting residue (**4**) was dissolved in toluene (8 mL) and refluxed at 115 °C for 1 h under nitrogen atmosphere. The solvent was evaporated under reduced pressure to obtain **5** as a dark-yellow powder (232.2 mg, 78%). Mp: 58.4–61.3 °C. IR (neat) _vmax_: 3018, 2247, 1777, 1197, 1181, 1170 cm^−1^. ^1^H NMR (400 MHz, CDCl_3_) δ 6.94 (1H, s, 14-H), 3.03–2.93 (1H, m), 2.93–2.87 (2H, m), 2.85–2.75 (1H, m), 2.32 (3H, s, OCOCH_3_), 2.29 (3H, s, OCOCH_3_), 1.92–1.85 (2H, m), 1.63–1.56 (2H, m), 1.53–1.46 (2H, m), 1.34 (1H, dd, *J* = 11.9, 1.4 Hz), 1.29–1.24 (1H, m), 1.22 (3H, d, *J* = 6.9 Hz, CHCH_3_), 1.16 (3H, d, *J* = 6.9 Hz, CHCH_3_), 1.03 (3H, s, CH_3_), 0.98 (3H, s, CH_3_); ^13^C NMR (100 MHz, CDCl_3_) δ 168.67 (OCOCH_3_), 168.10 (OCOCH_3_), 141.54, 141.20, 139.19, 135.78, 132.22, 125.17 (C14), 123.40 (NCO), 61.07, 53.29, 40.66, 37.32, 33.89, 32.85, 32.31, 27.61, 22.99, 22.93, 21.16, 21.02, 20.56, 19.43, 19.28. ESI-MS m/z: 436.3 [M + Na]^+^. Anal. Calcd. For C_24_H_31_NO_5_·0.2C_6_H_14_: C, 70.27; H, 7.91; N, 3.25. Found: C, 70.50; H, 7.57; N, 3.22%.

#### 3.1.4. Synthesis of Urea (**6**–**20**)—General Procedure

To a solution of **5** (100 mg, 0.24 mmol) in dry THF (5 mL) was added the respective amine and the reaction was stirred for 1 h at room temperature and nitrogen atmosphere, unless otherwise specified. The reaction mixture was evaporated under reduced pressure to remove the THF. The obtained residue was extracted using ethyl acetate (3 × 30 mL) from water (15 mL). The organic phase was then washed with 5% aqueous HCl (15 mL), water (15 mL), and brine (15 mL). The organic layer was dried over anhydrous Na_2_SO_4_, filtered, and evaporated under reduced pressure to afford the crude product. Finally, the crude product was purified by preparative TLC (PE/EtOAc) to obtain the urea product.

- (4b*R*,8a*S*)-2-isopropyl-8,8-dimethyl-4b-(3-methylureido)-4b,5,6,7,8,8a,9,10-octahydrophenanthrene-3,4-diyl diacetate (**6**)

Prepared according to general procedure from compound **5** (100 mg, 0.24 mmol), and methylamine 2 M in THF (0.18 mL, 0.36 mmol). The crude product was purified by preparative TLC (PE/EtOAc 1:2) to obtain **6** as a white powder (60.0 mg, 67%). Mp: 181.4–184.1 °C. IR (neat) _vmax_: 3374, 3331, 3011, 1777, 1768, 1647, 1566, 1196, 1173 cm^−1^. ^1^H NMR (400 MHz, CDCl_3_) δ 6.95 (1H, s, 14-H), 4.49 (1H, br s, NH), 4.06 (1H, br s, NH), 3.46–3.24 (1H, m), 2.99 (1H, ddd, *J* = 16.6, 6.7, 4.1 Hz), 2.93–2.79 (2H, m), 2.56 (3H, d, *J* = 3.1 Hz, NHCH_3_), 2.27 (6H, s, 2 × OCOCH_3_), 1.86–1.70 (3H, m), 1.61–1.47 (3H, m), 1.41 (1H, dd, *J* = 11.7, 4.2 Hz), 1.30–1.24 (1H, m), 1.20 (3H, d, *J* = 6.9 Hz, CHCH_3_), 1.16 (3H, d, *J* = 6.9 Hz, CHCH_3_), 1.04 (3H, s, CH_3_), 0.93 (3H, s, CH_3_); ^13^C NMR (100 MHz, CDCl_3_) δ 168.73 (OCOCH_3_), 168.60 (OCOCH_3_), 157.75 (CNHO), 141.24, 140.78, 139.12, 136.89, 133.06, 124.97 (C14), 56.99, 52.88, 40.83, 35.40, 34.05, 32.93, 30.76, 27.62, 27.31, 23.02, 22.91, 21.78, 21.18, 20.52, 19.20, 18.31. ESI-MS m/z: 445.2 [M + H]^+^, 467.4 [M + Na]^+^. Anal. Calcd. For C_25_H_36_N_2_O_5_·0.3C_6_H_14_: C, 68.43; H, 8.61; N, 5.96. Found: C, 68.64; H, 8.18; N, 5.95%.

- (4b*R*,8a*S*)-4b-(3-Ethylureido)-2-isopropyl-8,8-dimethyl-4b,5,6,7,8,8a,9,10-octahydrophenanthrene-3,4-diyl diacetate (**7**)

Prepared according to general procedure from compound **5** (100 mg, 0.24 mmol), and ethylamine 2 M in THF (0.18 mL, 0.36 mmol). The crude product was purified by preparative TLC (PE/EtOAc 1:1) to obtain **7** as a white powder (59.2 mg, 69%). Mp: 176.3–178.1 °C. IR (neat) _vmax_: 3368, 3319, 3014, 1779, 1763, 1645, 1558, 1197, 1174 cm^−1^. ^1^H NMR (400 MHz, CDCl_3_) δ 6.96 (1H, s, 14-H), 4.43 (1H, br s, NH), 3.96 (1H, br s, NH), 3.43–3.21 (1H, m), 3.04–2.99 (2H, m), 2.96 (1H, td, *J* = 6.8, 2.7 Hz), 2.92–2.79 (2H, m), 2.27 (6H, s, 2 × OCOCH_3_), 1.91–1.76 (3H, m), 1.62–1.47 (3H, m), 1.41 (1H, dd, *J* = 11.8, 4.1 Hz), 1.30–1.22 (1H, m), 1.19 (3H, d, *J* = 7.0 Hz, CHCH_3_), 1.17 (3H, d, *J* = 7.0 Hz, CHCH_3_), 1.05 (3H, s, CH_3_), 0.94 (3H, s, CH_3_), 0.89 (3H, t, *J* = 7.2 Hz, NHCH_2_CH_3_); ^13^C NMR (100 MHz, CDCl_3_) δ 168.57 (OCOCH_3_), 168.45 (OCOCH_3_), 157.21 (NHCO), 141.39, 141.05, 139.22, 136.97, 132.89, 124.96 (C14), 56.90, 52.98, 40.76, 35.63, 35.31, 33.98, 32.96, 30.90, 27.62, 23.05, 22.94, 21.81, 21.12, 20.51, 19.13, 18.33, 14.96. ESI-MS m/z: 459.2 [M + H]^+^. Anal. Calcd. For C_26_H_38_N_2_O_5_·0.5C_6_H_14_: C, 69.43; H, 9.04; N, 5.58. Found: C, 70.14; H, 8.30; N, 6.03%.

- (4b*R*,8a*S*)-2-isopropyl-8,8-dimethyl-4b-(3-propylureido)-4b,5,6,7,8,8a,9,10-octahydrophenanthrene-3,4-diyl diacetate (**8**)

Prepared according to general procedure from compound **5** (100 mg, 0.24 mmol), and propylamine (0.03 mL, 0.36 mmol). The crude product was purified by preparative TLC (PE/EtOAc 3:1) to obtain **8** as a white powder (64.9 mg, 73%). Mp: 103.6–105.8 °C. IR (neat) _vmax_: 3421, 3365, 3008, 1776, 1769, 1643, 1544, 1199, 1175 cm^−1^. ^1^H NMR (400 MHz, CDCl_3_) δ 6.97 (1H, s, 14-H), 4.49 (1H, br s, NH), 4.02 (1H, br s, NH), 3.31–3.19 (1H, m), 3.00–2.94 (2H, m), 2.92–2.80 (3H, m), 2.27 (3H, s, OCOCH_3_), 2.26 (3H, s, OCOCH_3_), 1.87–1.78 (3H, m), 1.63–1.57 (1H, m), 1.50 (2H, dt, *J* = 13.6, 3.5 Hz), 1.41 (1H, dd, *J* = 11.6, 4.1 Hz), 1.30–1.23 (3H, m), 1.18 (3H, d, *J* = 6.2 Hz, CHCH_3_), 1.17 (3H, d, *J* = 6.5 Hz, CHCH_3_), 1.05 (3H, s, CH_3_), 0.94 (3H, s, CH_3_), 0.71 (3H, t, *J* = 7.4 Hz, NHCH_2_CH_2_CH_3_); ^13^C NMR (100 MHz, CDCl_3_) δ 168.56 (OCOCH_3_), 168.39 (OCOCH_3_), 157.51 (NHCO), 141.46, 141.14, 139.32, 137.06, 132.68, 125.05 (C14), 56.85, 53.18, 42.36, 40.72, 35.92, 33.95, 32.97, 31.04, 27.62, 24.00, 23.05, 22.92, 21.83, 21.05, 20.51, 19.06, 18.36, 11.43. ESI-MS m/z: 473.2 [M + H]^+^. Anal. Calcd. For C_27_H_40_N_2_O_5_·0.2C_6_H_14_: C, 69.14; H, 8.81; N, 5.72. Found: C, 69.47; H, 8.60; N, 5.38%.

- (4b*R*,8a*S*)-4b-(3-butyl-3-methylureido)-2-isopropyl-8,8-dimethyl-4b,5,6,7,8,8a,9,10-octahydrophenanthrene-3,4-diyl diacetate (**9**)

Prepared according to general procedure from compound **5** (100 mg, 0.24 mmol), and N-butylmethylamine (0.04 mL, 0.36 mmol). The crude product was purified by preparative TLC (PE/EtOAc 3:1) to obtain **9** as a white powder (71.3 mg, 73%). Mp: 53.2–55.1 °C. IR (neat) _vmax_: 3469, 3021, 1774, 1663, 1521, 1200, 1176 cm^−1^. ^1^H NMR (400 MHz, CDCl_3_) δ 6.89 (1H, s, 14-H), 4.47 (1H, br s, NH), 3.17–3.08 (2H, m), 2.98–2.92 (1H, m), 2.89–2.82 (2H, m), 2.77 (3H, s, NCH_3_), 2.26 (6H, s, 2 × OCOCH_3_), 1.91–1.67 (5H, m), 1.48–1.39 (5H, m), 1.29–1.24 (3H, m), 1.20 (3H, d, *J* = 6.7 Hz, CHCH_3_), 1.14 (3H, d, *J* = 6.6 Hz, CHCH_3_), 1.00 (3H, s, CH_3_), 0.95 (3H, s, CH_3_), 0.88 (3H, t, *J* = 7.1 Hz, N(CH_3_)CH_2_CH_2_CH_2_CH_3_); ^13^C NMR (100 MHz, CDCl_3_) δ 168.79 (2 × OCOCH_3_), 155.70 (NHCO), 141.43, 139.74, 138.89, 135.77, 134.66, 124.45 (C14), 56.92, 53.02, 48.89, 40.96, 34.71, 34.58, 34.03, 32.95, 29.84, 30.47, 27.55, 23.16, 22.86, 21.75, 21.45, 20.58, 20.22, 19.64, 18.44, 14.08. ESI-MS m/z: 501.3 [M + H]^+^. Anal. Calcd. For C_29_H_44_N_2_O_5_: C, 69.57; H, 8.86; N, 5.60. Found: C, 70.24; H, 8.99; N, 5.71%.

- (4b*R*,8a*S*)-4b-(3-isobutylureido)-2-isopropyl-8,8-dimethyl-4b,5,6,7,8,8a,9,10-octahydrophenanthrene-3,4-diyl diacetate (**10**)

Prepared according to general procedure from compound **5** (100 mg, 0.24 mmol), and isobutylamine (0.04 mL, 0.36 mmol). The crude product was purified by preparative TLC (PE/EtOAc 2:1) to obtain **10** as a white powder (66.4 mg, 74%). Mp: 115.2–117.4 °C. IR (neat) _vmax_: 3425, 3382, 3005, 1776, 1637, 1542, 1199, 1176 cm^−1^. ^1^H NMR (400 MHz, CDCl_3_) δ 6.98 (1H, s, 14-H), 4.49 (1H, br s, NH), 4.06 (1H, br s, NH), 3.25–3.11 (1H, m), 2.99 (1H, dt, *J* = 16.9, 5.1 Hz), 2.92–2.82 (3H, m), 2.78–2.69 (1H, m), 2.27 (3H, s, OCOCH_3_), 2.26 (3H, s, OCOCH_3_), 1.87–1.70 (4H, m), 1.61 (1H, dt, *J* = 14.2, 3.7 Hz), 1.53–1.48 (2H, m), 1.41 (1H, dd, *J* = 11.1, 4.6 Hz), 1.30–1.23 (1H, m), 1.17 (3H, d, *J* = 6.9 Hz, CHCH_3_), 1.16 (3H, d, *J* = 6.9 Hz, CHCH_3_), 1.06 (3H, s, CH_3_), 0.94 (3H, s, CH_3_), 0.69 (6H, d, *J* = 6.6 Hz, NHCH_2_CH(CH_3_)(CH_3_)); ^13^C NMR (100 MHz, CDCl_3_) δ 168.61 (OCOCH_3_), 168.32 (OCOCH_3_), 157.70 (NHCO), 141.50, 141.20, 139.37, 137.15, 132.42, 125.17 (C14), 56.74, 53.32, 48.16, 40.64, 36.17, 33.90, 32.99, 31.14, 28.40, 27.59, 23.05, 22.85, 21.84, 21.02, 20.52, 20.23, 20.18, 19.00, 18.35. ESI-MS m/z: 487.2 [M + H]^+^. Anal. Calcd. For C_28_H_42_N_2_O_5_·0.3C_6_H_14_: C, 69.84; H, 9.09; N, 5.47. Found: C, 70.11; H, 8.84; N, 5.60%.

- (4b*R*,8a*S*)-4b-(3-(3-(dimethylamino)-2,2-dimethylpropyl)ureido)-2-isopropyl-8,8-dimethyl-4b,5,6,7,8,8a,9,10-octahydrophenanthrene-3,4-diyl diacetate (**11**)

Prepared according to general procedure from compound **5** (100 mg, 0.24 mmol), and *N*,*N*,2,2-tetramethyl-1,3-propanediamine (0.06 mL, 0.36 mmol) to obtain **11** as a white powder (73.1 mg, 56%). Mp: 248.4–250.4 °C. IR (neat) _vmax_: 3248, 3005, 1758, 1644, 1580, 1209, 1200, 1180 cm^−1^. ^1^H NMR (400 MHz, CDCl_3_) δ 8.58 (1H, br s, NHCH_2_), 7.60 (1H, br s, NH), 6.86 (1H, s, 14-H), 3.34–3.18 (2H, m,), 3.06 (1H, dd, *J* = 15.0, 7.1 Hz, NHCH_2_), 2.97–2.90 (1H, m, 7-Ha), 2.86–2.74 (3H, m, NHCH_2_, 7-Hb, 15-H), 2.59 (6H, s, OCOCH_3_, NCH_3_), 2.44–2.32 (2H, m), 2.30–2.24 (1H, m, NCH_2_), 2.23 (3H, s, OCOCH_3_), 2.13 (3H, d, *J* = 3.5 Hz, NCH_3_), 1.74–1.69 (1H, m), 1.65–1.56 (2H, m), 1.48 (1H, td, *J* = 13.3, 4.2 Hz), 1.36 (1H, dd, *J* = 12.9, 2.8 Hz, 5-H), 1.27–1.21 (1H, m), 1.14 (3H, d, *J* = 6.9 Hz, CHCH_3_), 1.08 (3H, d, *J* = 6.8 Hz, CHCH_3_), 1.04 (6H, s, NHCH_2_C(CH_3_)(CH_3_), CH_3_), 0.99 (3H, s, NHCH_2_C(CH_3_)(CH_3_)), 0.92 (3H, s, CH_3_); ^13^C NMR (100 MHz, CDCl_3_) δ 169.96 (OCOCH_3_), 168.58 (OCOCH_3_), 160.93 (NHCO), 140.85, 139.29, 138.73, 138.41, 133.06, 123.23 (C14), 64.47 (NCH_2_), 56.27 (C10), 53.49 (C5), 46.77 (NHCH_2_), 45.72 (NCH_3_), 43.29 (NCH_3_), 40.92, 37.32, 36.33 (NHCH_2_C(CH_3_)(CH_3_)), 34.03 (C4), 33.02, 31.75 (C7), 27.47 (C15), 26.93, 26.84, 23.35, 22.99, 21.93, 21.69, 20.61, 19.17, 18.45. ESI-MS m/z: 544.4 [M + H]^+^. Anal. Calcd. For C_31_H_49_N_3_O_5_·3H_2_O: C, 62.29; H, 9.27; N, 7.03. Found: C, 62.61; H, 9.44; N, 7.25%.

- (4b*R*,8a*S*)-2-isopropyl-4b-(3-methoxyureido)-8,8-dimethyl-4b,5,6,7,8,8a,9,10-octahydrophenanthrene-3,4-diyl diacetate (**12**)

Prepared according to general procedure from compound **5** (100 mg, 0.24 mmol), methoxyamine hydrochloride (60.0 mg, 0.72 mmol), and Et_3_N (0.70 mL, 5.00 mmol). The reaction was stirred for 24 h at room temperature under nitrogen atmosphere. The crude product was purified by preparative TLC (PE/EtOAc 1:1) to obtain **12** as a white powder (75.8 mg, 70%). Mp: 110.5–112.3 °C. IR (neat) _vmax_: 3406, 3009, 1773, 1681, 1531, 1202, 1176 cm^−1^. ^1^H NMR (400 MHz, CDCl_3_) δ 6.92 (1H, s, 14-H), 6.64 (1H, br s, NH), 5.96 (1H, br s, NH), 3.64 (3H, s, NHOCH_3_), 3.62–3.55 (1H, m), 2.95 (1H, dd, *J* = 5.3, 3.2 Hz, 7-Ha), 2.93–2.82 (m, 2H, 7-Hb, 15-H), 2.27 (3H, s, OCOCH_3_), 2.26 (3H, s, OCOCH_3_), 1.90–1.72 (3H, m), 1.68–1.63 (1H, m), 1.54–1.47 (2H, m), 1.44 (1H, dd, *J* = 11.3, 3.9 Hz, 5-H), 1.34–1.27 (1H, m), 1.21 (3H, d, *J* = 6.9 Hz, CHCH_3_), 1.13 (3H, d, *J* = 6.9 Hz, CHCH_3_), 1.00 (3H, s, CH_3_), 0.98 (3H, s, CH_3_); ^13^C NMR (100 MHz, CDCl_3_) δ 168.87 (OCOCH_3_), 168.69 (OCOCH_3_), 157.63 (NHCO), 141.35, 140.12, 138.87, 136.46, 132.96, 124.59 (C14), 64.21 (OCH_3_), 56.35 (C10), 53.21, 40.77, 34.90, 33.97 (C4), 32.96, 31.50 (C7), 27.54 (C15), 23.20, 22.76, 21.48, 21.38, 20.59, 19.45, 18.41. ESI-MS m/z: 483.3 [M + Na]^+^. Anal. Calcd. For C_25_H_36_N_2_O_6_·0.75H_2_O: C, 63.34; H, 7.97; N, 5.91. Found: C, 63.61; H, 8.51; N, 5.91%.

- (4b*R*,8a*S*)-2-isopropyl-8,8-dimethyl-4b-(3-phenethylureido)-4b,5,6,7,8,8a,9,10-octahydrophenanthrene-3,4-diyl diacetate (**13**)

Prepared according to general procedure from compound **5** (100 mg, 0.24 mmol), and phenethylamine (0.05 mL, 0.36 mmol). The crude product was purified by preparative TLC (PE/EtOAc 3:1) to obtain **13** as a white powder (66.7 mg, 58%). Mp: 114.5–116.9 °C. IR (neat) _vmax_: 3425, 3379, 3026, 1776, 1660, 1544, 1200, 1176 cm^−1^. ^1^H NMR (400 MHz, CDCl_3_) δ 7.27–7.22 (2H, m, Ar-H), 7.20–7.15 (1H, m, Ar-H), 7.13–7.09 (2H, m, Ar-H), 6.90 (1H, s, 14-H), 4.47 (1H, br s, NH), 4.11 (1H, br s, NH), 3.34–3.17 (3H, m), 2.92–2.87 (1H, m), 2.80–2.73 (2H, m), 2.67–2.57 (2H, m), 2.27 (3H, s, OCOCH_3_), 2.20 (3H, s, OCOCH_3_), 1.80–1.61 (4H, m), 1.51–1.45 (2H, m), 1.37 (1H, dd, *J* = 11.9, 4.0 Hz), 1.27–1.23 (1H, m), 1.20 (3H, d, *J* = 6.9 Hz, CHCH_3_), 1.16 (3H, d, *J* = 6.9 Hz, CHCH_3_), 1.03 (3H, s, CH_3_), 0.92 (3H, s, CH_3_); ^13^C NMR (100 MHz, CDCl_3_) δ 168.62 (OCOCH_3_), 168.54 (OCOCH_3_), 157.00 (NHCO), 141.22, 140.85, 139.72, 139.09, 136.94, 132.64, 128.93 (2 × ArC), 128.43 (2 × ArC), 126.20 (ArC), 125.04 (C14), 56.82, 53.00, 41.53, 40.70, 36.01, 35.50, 33.96, 32.91, 30.65, 27.58, 22.99 (2C), 21.78, 21.08, 20.54, 19.09, 18.25. ESI-MS m/z: 557.5 [M + Na]^+^. Anal. Calcd. For C_32_H_42_N_2_O_5_·1H_2_O: C, 69.54; H, 8.02; N, 5.07. Found: C, 69.47; H, 8.55; N, 4.90%.

- (4b*R*,8a*S*)-4b-(3-(3-chlorophenethyl)ureido)-2-isopropyl-8,8-dimethyl-4b,5,6,7,8,8a,9,10-octahydrophenanthrene-3,4-diyl diacetate (**14**)

Prepared according to general procedure from compound **5** (100 mg, 0.24 mmol), and 2-(3-chlorophenyl) ethylamine (0.05 mL, 0.36 mmol). The crude product was purified by preparative TLC (PE/EtOAc 2:1) to obtain **14** as a white powder (86.6 mg, 74%). Mp: 116.8–118.3 °C. IR (neat) _vmax_: 3428, 3379, 3023, 3006, 1773, 1769, 1660, 1544, 1201, 1176 cm^−1^. ^1^H NMR (400 MHz, CDCl_3_) δ 7.18–7.14 (2H, m, Ar-H), 7.11–7.09 (1H, m, Ar-H), 7.01–6.98 (1H, m, Ar-H), 6.93 (1H, s, 14-H), 4.52 (1H, br s, NH), 4.11 (1H, br s, NH), 3.32–3.15 (3H, m), 2.93–2.86 (1H, m, 15-H), 2.80–2.73 (2H, m, 7-H), 2.65–2.59 (1H, m, NHCH_2_CH_2_), 2.57–2.49 (1H, m, NHCH_2_CH_2_), 2.28 (3H, s, OCOCH_3_), 2.21 (3H, s, OCOCH_3_), 1.80–1.62 (4H, m), 1.52–1.46 (2H, m), 1.38 (1H, dd, *J* = 11.7, 4.5 Hz, H-5), 1.28–1.23 (1H, m), 1.20 (3H, d, *J* = 6.9 Hz, CHCH_3_), 1.16 (3H, d, *J* = 6.9 Hz, CHCH_3_), 1.03 (3H, s, CH_3_), 0.92 (3H, s, CH_3_); ^13^C NMR (100 MHz, CDCl_3_) δ 168.61 (OCOCH_3_), 168.54 (OCOCH_3_), 157.07 (NHCO), 141.97 (ArC), 141.23, 141.00, 139.10, 137.10, 134.11 (Ar-Cl), 132.40, 129.68 (ArC), 129.06 (ArC), 127.16 (ArC), 126.37 (ArC), 125.08 (C14), 56.90 (C10), 52.95 (C5), 41.32 (NHCH_2_), 40.67, 35.70, 35.63, 33.98 (C4), 32.90, 30.63 (C7), 27.60 (C15), 23.02, 22.95, 21.78, 21.05, 20.54, 19.03, 18.26. ESI-MS m/z: 569.2 [M + H]^+^, 591.4 [M + Na]^+^. Anal. Calcd. For C_32_H_41_ClN_2_O_5_·0.75H_2_O: C, 65.97; H, 7.35; N, 4.81. Found: C, 66.16; H, 7.85; N, 4.66%.

- (4b*R*,8a*S*)-2-isopropyl-8,8-dimethyl-4b-(3-(pyridin-4-ylmethyl)ureido)-4b,5,6,7,8,8a,9,10-octahydrophenanthrene-3,4-diyl diacetate (**15**)

Prepared according to general procedure from compound **5** (100 mg, 0.24 mmol), and 1-(pyridin-4-yl)methanamine (0.04 mL, 0.36 mmol). The reaction was stirred for 3 h at room temperature under nitrogen atmosphere. The crude product was purified by preparative TLC (PE/EtOAc 1:5) to obtain **15** as a white powder (24.7 mg, 49%). Mp: 134.8–137.0 °C. IR (neat) _vmax_: 3420, 3374, 3028, 3008, 1772, 1667, 1545, 1202, 1176 cm^−1^. ^1^H NMR (400 MHz, CDCl_3_) δ 8.44 (2H, d, *J* = 4.2 Hz, Ar-H), 7.02 (2H, d, *J* = 4.8 Hz, Ar-H), 6.92 (1H, s, 14-H), 4.67 (1H, br s, NH), 4.60 (1H, br s, NH), 4.26–4.10 (2H, m, ArCH_2_NH), 3.37–3.17 (1H, m), 2.92–2.79 (3H, m), 2.27 (3H, s, OCOCH_3_), 2.20 (3H, s, OCOCH_3_), 1.87–1.76 (3H, m), 1.62–1.50 (3H, m), 1.43 (1H, dd, *J* = 11.9, 4.1 Hz), 1.29–1.24 (1H, m), 1.15 (6H, d, *J* = 6.8 Hz, CH(CH_3_)(CH_3_)), 1.06 (3H, s, CH_3_), 0.94 (3H, s, CH_3_); ^13^C NMR (100 MHz, CDCl_3_) δ 168.82 (OCOCH_3_), 168.59 (OCOCH_3_), 156.95 (NHCO), 149.70 (2C, CHNCH), 149.00 (ArC), 141.25 (2C), 139.16, 137.02, 132.58, 125.14 (C14), 122.38 (2C, CHCHNCHCH), 57.18, 53.03, 43.51, 40.63, 35.47, 34.11, 32.91, 30.71, 27.58, 22.95 (2C), 21.76, 21.08, 20.52, 19.10, 18.28. ESI-MS m/z: 522.4 [M + H]^+^. Anal. Calcd. For C_30_H_39_N_3_O_5_·0.75H_2_O: C, 67.33; H, 7.63; N, 7.85. Found: C, 67.30; H, 8.12; N, 7.38%.

- (4b*R*,8a*S*)-2-isopropyl-8,8-dimethyl-4b-(3-(3-(methyl(phenyl)amino)propyl)ureido)-4b,5,6,7,8,8a,9,10-octahydrophenanthrene-3,4-diyl diacetate (**16**)

Prepared according to general procedure from compound **5** (100 mg, 0.24 mmol), and N-(3-aminopropyl)-N-methyl-N-phenylamine (0.06 mL, 0.36 mmol). The reaction was stirred for 3 h at room temperature under nitrogen atmosphere. The crude product was purified by preparative TLC (PE/EtOAc 1:1) to obtain **16** as a white powder (57.2 mg, 48%). Mp: 90.2–92.4 °C. IR (neat) _vmax_: 3385, 3022, 3003, 1773, 1769, 1600, 1545, 1198, 1176 cm^−1^. ^1^H NMR (400 MHz, CDCl_3_) δ 7.19 (2H, t, *J* = 7.8 Hz, Ar-H), 6.96 (1H, s, 14-H), 6.70–6.61 (3H, m, Ar-H), 4.49 (1H, br s, NH), 4.13 (1H, br s, NH), 3.35–3.21 (1H, m), 3.17 (2H, t, *J* = 7.4 Hz, NCH_2_), 3.08–2.96 (3H, m, 7-Ha, NHCH_2_), 2.93–2.85 (2H, m, 7-Hb, 15-H), 2.84 (3H, s, NCH_3_), 2.27 (3H, s, OCOCH_3_), 2.25 (3H, s, OCOCH_3_), 1.86–1.75 (3H, m), 1.64–1.47 (5H, m,), 1.42 (1H, dd, *J* = 11.7, 4.2 Hz, 5-H), 1.30–1.25 (1H, m), 1.15 (6H, d, *J* = 6.7 Hz, CH(CH_3_)(CH_3_)), 1.05 (3H, s, CH_3_), 0.94 (3H, s, CH_3_); ^13^C NMR (100 MHz, CDCl_3_) δ 168.58 (2 × OCOCH_3_), 157.22 (NHCO), 149.33 (ArC), 141.29, 141.08, 139.18, 137.01, 132.68, 129.24 (2 × ArC), 125.06 (C14), 116.12 (ArC), 112.30 (2 × ArC), 56.92 (C10), 52.96 (C5), 50.40 (NCH_2_), 40.68, 38.51 (NHCH_2_), 38.42 (NCH_3_), 35.62, 33.99 (C4), 32.93, 30.85 (C7), 27.57 (C15), 27.10 (NHCH_2_CH_2_), 22.93 (2C), 21.79, 21.13, 20.52, 19.07, 18.31. ESI-MS m/z: 578.4 [M + H]^+^. Anal. Calcd. For C_34_H_47_N_3_O_5_·0.25H_2_O: C, 70.13; H, 8.22; N, 7.22. Found: C, 70.31; H, 8.35; N, 7.01%.

- (4b*R*,8a*S*)-4b-(3-(furan-3-ylmethyl)ureido)-2-isopropyl-8,8-dimethyl-4b,5,6,7,8,8a,9,10-octahydrophenanthrene-3,4-diyl diacetate (**17**)

Prepared according to general procedure from compound **5** (100 mg, 0.24 mmol) and 1-(furan-3-yl)methanamine (0.03 mL, 0.36 mmol). The crude product was purified by preparative TLC (PE/EtOAc 2:1) to obtain **17** as a white powder (81.0 mg, 74%). Mp: 142.6–145.0 °C. IR (neat) _vmax_: 3429, 3369, 3007, 1772, 1643, 1539, 1201, 1176 cm^−1^. ^1^H NMR (400 MHz, CDCl_3_) δ 7.27 (1H, t, *J* = 1.5 Hz, Ar-H), 7.17 (1H, s, Ar-H), 6.90 (1H, s, 14-H), 6.20 (1H, s, Ar-H), 4.60 (1H, br s, NH), 4.25 (1H, br s, NH), 4.06–3.94 (2H, m, ArCH_2_NH), 3.34–3.22 (1H, m), 2.90–2.80 (3H, m), 2.27 (3H, s, OCOCH_3_), 2.21 (3H, s, OCOCH_3_), 1.85–1.74 (3H, m), 1.60 (1H, dt, *J* = 14.8, 3.9 Hz), 1.51–1.46 (2H, m), 1.39 (1H, dd, *J* = 11.9, 3.8 Hz), 1.29–1.25 (1H, m), 1.17 (3H, d, *J* = 6.9 Hz, CHCH_3_), 1.15 (3H, d, *J* = 6.9 Hz, CHCH_3_), 1.03 (3H, s, CH_3_), 0.93 (3H, s, CH_3_); ^13^C NMR (100 MHz, CDCl3) δ 168.64 (OCOCH_3_), 168.51 (OCOCH_3_), 157.22 (NHCO), 143.06 (ArC), 141.35, 141.09, 139.96 (ArC), 139.23, 136.91, 132.49, 125.07 (C14), 123.05 (ArC), 110.39 (ArC), 56.82, 53.07, 40.66, 35.75, 35.66, 33.90, 32.94, 30.97, 27.57, 22.97, 22.88, 21.77, 20.98, 20.53, 19.04, 18.22. ESI-MS m/z: 511.2 [M + H]^+^, 533.4 [M + Na]^+^. Anal. Calcd. For C_29_H_38_N_2_O_6_: C, 68.21; H,7.50; N, 5.49. Found: C, 68.71; H, 7.65; N, 4.87%.

- (4b*R*,8a*S*)-2-isopropyl-8,8-dimethyl-4b-(3-((tetrahydro-2*H*-pyran-4-yl)methyl)ureido)-4b,5,6,7,8,8a,9,10-octahydrophenanthrene-3,4-diyl diacetate (**18**)

Prepared according to general procedure from compound **5** (100 mg, 0.24 mmol), and (oxan-4-yl)methanamine (0.04 mL, 0.36 mmol). The crude product was purified by preparative TLC (PE/EtOAc 1:2) to obtain **18** as a white powder (90.6 mg, 83%). Mp: 129.6–131.7 °C. IR (neat) _vmax_: 3424, 3381, 3006, 1775, 1666, 1542, 1199, 1176 cm^−1^. ^1^H NMR (400 MHz, CDCl_3_) δ 6.98 (1H, s, 14-H), 4.52 (1H, br s, NH), 4.15 (1H, br s, NH), 3.88–3.83 (2H, m, OCH_2_), 3.27 (2H, td, *J* = 11.7, 1.7 Hz, OCH_2_), 3.22–3.12 (1H, m), 3.00–2.83 (5H, m, 15-H, CH_2_NH), 2.28 (3H, s, OCOCH_3_), 2.27 (3H, s, OCOCH_3_), 1.88–1.76 (3H, m), 1.66–1.55 (2H, m), 1.53–1.48 (2H, m), 1.41 (1H, dd, *J* = 11.5, 4.2 Hz, 5-H), 1.36–1.31 (2H, m), 1.27–1.22 (1H, m), 1.18 (3H, d, *J* = 6.7 Hz, CHCH_3_), 1.17 (3H, d, *J* = 6.7 Hz, CHCH_3_), 1.14–1.07 (2H, m), 1.05 (3H, s, CH_3_), 0.94 (3H, s, CH_3_); ^13^C NMR (100 MHz, CDCl_3_) δ 168.56 (OCOCH_3_), 168.42 (OCOCH_3_), 157.55 (NHCO), 141.35, 141.29, 139.30, 137.17, 132.41, 125.08 (C14), 67.79 (2C, CH_2_OCH_2_), 56.86 (C10), 53.03 (C5), 46.29 (CH_2_NH), 40.61, 35.96, 35.23, 33.95, 32.96 (C4), 31.02, 30.64, 30.61, 27.58 (C15), 23.10, 22.91, 21.80, 21.08, 20.53, 18.99, 18.33. ESI-MS m/z: 529.2 [M + H]^+^, 551.4 [M + Na]^+^. Anal. Calcd. For C_30_H_44_N_2_O_6_: C, 68.16; H, 8.39; N, 5.30. Found: C, 67.96; H, 8.30; N, 5.04%.

- (4b*R*,8a*S*)-2-isopropyl-8,8-dimethyl-4b-(3-(oxetan-3-yl)ureido)-4b,5,6,7,8,8a,9,10-octahydrophenanthrene-3,4-diyl diacetate (**19**)

Prepared according to general procedure from compound **5** (100 mg, 0.24 mmol), and oxetan-3-amine (0.03 mL, 0.36 mmol). The reaction was stirred for 3 h at room temperature under nitrogen atmosphere. The crude product was purified by preparative TLC (PE/EtOAc 1:3) to obtain **19** as a white powder (92.4 mg, 77%). Mp: 123.8–126.6 °C. IR (neat) _vmax_: 3410, 3359, 3008, 1771, 1667, 1546, 1203, 1191, 1177 cm^−1^. ^1^H NMR (400 MHz, CDCl_3_) δ 7.00 (1H, s, 14-H), 4.77–4.70 (3H, m, C_3_H_5_O), 4.59 (2H, br s, 2 × NH), 4.21 (1H, t, *J* = 5.1 Hz, C_3_H_5_O), 4.18–4.11 (1H, m, C_3_H_5_O), 3.39–3.19 (1H, m), 3.02–2.95 (1H, m, 7-Ha), 2.93–2.85 (2H, m, 7-Hb, 15-H), 2.28 (3H, s, OCOCH_3_), 2.26 (3H, s, OCOCH_3_), 1.92–1.86 (1H, m), 1.81–1.72 (2H, m), 1.66–1.59 (1H, m), 1.56–1.48 (2H, m), 1.42 (1H, dd, *J* = 12.1, 3.4 Hz, 5-H), 1.32–1.26 (1H, m), 1.20 (3H, d, *J* = 6.9 Hz, CHCH_3_), 1.17 (3H, d, *J* = 6.9 Hz, CHCH_3_), 1.02 (3H, s, CH_3_), 0.95 (3H, s, CH_3_); ^13^C NMR (100 MHz, CDCl_3_) δ 168.62 (OCOCH_3_), 168.43 (OCOCH_3_), 156.25 (NHCO), 141.55 (2C), 139.34, 136.91, 132.41, 125.15 (C14), 79.39 (OCH_2_), 79.15 (OCH_2_), 56.96 (C10), 53.35 (C5), 45.37 (CHNH), 40.57, 35.74, 33.88 (C4), 32.95, 31.34 (C7), 27.65 (C15), 23.13, 22.84, 21.74, 21.10, 20.52, 19.04, 18.30. ESI-MS m/z: 509.3 [M + Na]^+^. Anal. Calcd. For C_27_H_38_N_2_O_6_·0.25H_2_O: C, 66.03; H, 7.90; N, 5.70. Found: C, 66.02; H, 7.96; N, 5.45%.

- (4b*R*,8a*S*)-2-isopropyl-8,8-dimethyl-4b-(3-((3-methyloxetan-3-yl)methyl)ureido)-4b,5,6,7,8,8a,9,10-octahydrophenanthrene-3,4-diyl diacetate (**20**)

Prepared according to general procedure from compound **5** (100 mg, 0.24 mmol) and (3-methyloxetan-3-yl) methanamine hydrochloride (49.5 mg, 0.36 mmol), along with addition of Et_3_N (0.23 mL, 1.63 mmol) to reaction mixture. The mixture was stirred for 6 h at room temperature under a nitrogen atmosphere. The crude product was purified by preparative TLC (PE/EtOAc 1:5) to obtain **20** as a white powder (55.0 mg, 50%). Mp: 142.3–144.6 °C. IR (neat) _vmax_: 3396, 3006, 1773, 1670, 1542, 1200, 1176 cm^−1^. ^1^H NMR (400 MHz, CDCl_3_) δ 7.00 (1H, s, 14-H), 4.71 (1H, br s, NH), 4.35 (1H, br s, NH), 4.30 (1H, d, *J* = 5.8 Hz, OCH_2_), 4.19–4.15 (2H, m, OCH_2_), 4.12–4.05 (1H, m, OCH_2_), 3.28–3.21 (1H, m, CH_2_NH), 3.17–3.10 (1H, m), 3.04–3.00 (1H, m, CH_2_NH), 3.00–2.95 (1H, m, 7-Ha), 2.91–2.83 (2H, m, 15-H, 7-Hb), 2.27 (3H, s, OCOCH_3_), 2.26 (3H, s, OCOCH_3_), 1.87–1.81 (2H, m), 1.79–1.75 (1H, m), 1.66–1.60 (1H, m), 1.56–1.48 (2H, m), 1.41 (1H, dd, *J* = 9.6, 5.9 Hz, 5-H), 1.31–1.26 (1H, m), 1.18 (3H, d, *J* = 7.4 Hz, CHCH_3_), 1.16 (3H, d, *J* = 7.3 Hz, CHCH_3_), 1.06 (6H, s, C_3_H_4_O-CH_3_, CH_3_), 0.95 (3H, s, CH_3_). ^13^C NMR (100 MHz, CDCl_3_) δ 168.61 (OCOCH_3_), 168.37 (OCOCH_3_), 158.30 (NHCO), 141.48, 141.41, 139.41, 137.48, 131.89, 125.43 (C14), 80.34 (OCH_2_), 80.25 (OCH_2_), 56.79 (C10), 53.54 (C5), 46.99 (CH_2_NH), 40.52, 39.47 (OCH_2_C), 36.53, 33.84 (C4), 32.99, 31.24 (C7), 27.60 (C15), 22.98, 22.87, 22.23, 21.84, 20.95, 20.51, 18.88, 18.38. ESI-MS m/z: 537.4 [M + Na]^+^. Anal. Calcd. For C_29_H_42_N_2_O_6_: C, 67.68; H, 8.23; N, 5.44. Found: C, 67.48; H, 8.14; N, 5.22%.

### 3.2. Biological Assays

#### 3.2.1. Materials

Dulbecco’s Modified Eagle Medium (DMEM) with 4 mM L-glutamine and phenol red, and without D-glucose, sodium pyruvate, and HEPES (#11966025); DMEM with 25 mM D-glucose and phenol red, and without L-glutamine, sodium pyruvate, and HEPES (#11960044); trypsin-EDTA (0.05%) with phenol red; Fetal Bovine Serum (FBS) (#10270-106); L-glutamine (200 mM); sodium pyruvate (100 mM); Penicillin (10,000 U/mL)/Streptomycin (10,000 U/mL) (P/S) solution; Hanks’ Balanced Salt Solution (HBSS) with calcium, magnesium and 5.56 mM D-glucose without phenol red and sodium pyruvate; Trypan Blue solution (0.4%); Annexin V-FITC; PI; Dimethyl Sulfoxide (DMSO) (≥99.7%); Pierce^TM^ BCA protein assay reagents A and B; and Pierce^TM^ bovine serum albumin standard ampules (2 mg/mL) were obtained from Thermo Fisher Scientific (Waltham, MA, USA). Ham’s F12 with 10 mM glucose, 1 mM L-glutamine, and phenol red (#L0135) were obtained from BioWest (Nuaillé, France). HyClone Dulbecco’s Phosphate Buffered Saline (DPBS) without calcium and magnesium was obtained from Cytiva (Marlborough, MA, USA). D-(+)-Glucose solution (405–495 g/L), H_2_DCFDA, HEPES, CaCl_2_, dithiothreitol (DTT), Na-EDTA, Triton X-100, NaOH, HCl, ethanol, sodium deoxycholate, Tween-20, and sodium dodecyl sulphate (SDS) were obtained from Sigma-Aldrich (St. Louis, MO, USA). Protease inhibitor cocktail, phosphatase inhibitor cocktail, Immobilon^®^-P Polyvinylidene Fluoride (PVDF) membranes, and Immobilon Western Chemiluminescent HRP Substrate Kit were obtained from Merck Millipore (Darmstadt, Germany). Thiazolyl Blue Tetrazolium Bromide (MTT), DMSO (≥99.5%), NaCl, NaH_2_PO_4_·2H_2_O, Na_2_HPO_4_·12H_2_O, acrylamide, and Tris were obtained from PanReac AppliChem (Barcelona, Spain). RNase powder from the bovine pancreas was obtained from Roche Diagnostics (Rotkreuz, Switzerland). Skimmed milk was obtained from Nestlé (Vevey, Switzerland). Blue-sensitive X-ray films (CP-BU) were obtained from the AGFA (Mortsel, Belgium). Primary antibodies against Cdk4 (rabbit polyclonal IgG) (sc-260) and Cdk6 (rabbit polyclonal IgG) (sc-177) were obtained from Santa Cruz Biotechnology (Dallas, TX, USA). Primary antibody against actin (monoclonal mouse) (#691001) was obtained from MP Biomedicals (Santa Ana, CA, USA). Primary antibody against SOD2 (MnSOD) (rabbit monoclonal) (ab68155), and anti-rabbit (ab6721) and anti-mouse (ab6728) HRP-conjugated secondary antibodies were obtained from Abcam (Cambridge, UK).

#### 3.2.2. Compounds

CA **1** and its derivatives were stored at −20 °C in powder form. Before each experiment, the compounds were weighed, dissolved in DMSO, and subsequently diluted in the culture medium to obtain working solutions at the required concentration. The final concentration of DMSO in contact with the cells was always equal to or lower than 0.5% for all the working solutions.

#### 3.2.3. Cell Culture

All cell lines were obtained from American Type Culture Collection (ATCC, Manassas, VA, USA). HCT116 cells were maintained in a DMEM (without D-glucose)/Ham’s F12 mixture (1:1) supplemented with 7.5 mM D-glucose, 10% FBS, and 0.5% P/S solution. SW620 and SW480 cells were cultured in DMEM (without D-glucose) supplemented with 12.5 mM D-glucose, 5% FBS, and 1% P/S solution. Caco-2 cells were maintained in DMEM (without D-glucose) supplemented with 10 mM D-glucose, 10% FBS, and 1% P/S solution. A375 and MiaPaca-2 cells were maintained in DMEM (without D-glucose) supplemented with 25 mM D-glucose, 10% FBS, and 1% P/S solution. BJ cells were cultured in DMEM (high glucose) supplemented with 10% FBS, 1% P/S solution, 2 mM L-glutamine, and 1 mM sodium pyruvate. All the cells were incubated at 37 °C in a humidified atmosphere containing 5% CO_2_. Sub-confluent monolayers of cells were used in all experiments.

#### 3.2.4. Cell Viability Assay

The effect of the compounds on cell viability was evaluated using the MTT assay. HCT116 (3000 cells/well), SW620 (8000 cells/well), SW480 (6000 cells/well), Caco-2 (4000 cells/well), A375 (1500 cells/well), MiaPaca-2 (2000 cells/well), and BJ (10,000 cells/well) cells were seeded in 96-well plates. After 24 h, the growth medium was replaced with a fresh medium containing the desired concentration of the compound or DMSO (control). After 72 h of incubation, the supernatant was removed and 100 µL of a mixture (1:1) of filtered MTT solution (1 mg/mL in PBS) with FBS-free medium was added to each well. After 1 h of incubation at 37 °C, the mixture was removed and 100 µL of DMSO was added to each well to dissolve the formed formazan crystals. Absorbance was immediately measured at 570 nm using a Benchmark Plus Microplate Reader (Bio-Rad Laboratories, Hercules, CA, USA). IC_50_ values were calculated using GraphPad Prism 9 software (GraphPad Software, San Diego, CA, USA) and expressed as the mean ± standard deviation (SD) of at least three independent experiments.

#### 3.2.5. Cell Proliferation Assay and Cell Doubling Time

SW480 cells were seeded at a concentration of 1.68 × 10^5^ cells per well in 6-well plates and incubated at 37 °C. After 24 h, cells were incubated with fresh culture medium (control) or treated with compound **14** (IC_50_ concentration) for 24, 48, and 72 h. Prior to treatment initiation (0 h), cells were trypsinized, washed with DPBS, and collected by centrifugation. The collected cells were resuspended in DPBS containing trypan blue and examined using an Invitrogen Countess 3 Automated Cell Counter (Thermo Fisher Scientific, Waltham, MA, USA) to determine the cell number and viability. This procedure was replicated at various treatment time points (24, 48, and 72 h) to generate a cell proliferation curve depicting the changes over time. Cell doubling time was calculated by dividing the natural logarithm of 2 by the growth rate [65].

#### 3.2.6. Apoptosis Assay

SW480 cells (1.68 × 10^5^ cells/well) were seeded in 6-well plates and incubated at 37 °C for 24 h. Subsequently, the growth medium was replaced with fresh medium (control) or compound **14** at the previously determined IC_50_ concentration. Following incubation for 24, 48, or 72 h, the adherent cells were gently detached by trypsinization, washed with DPBS, and collected by centrifugation. Similarly, the cells in suspension were washed with DPBS and collected by centrifugation, along with the adhered cells. After centrifugation, the cells were resuspended in 95 µL binding buffer (10 mM Hepes/NaOH pH 7.4, 140 mM NaCl, 2.5 mM CaCl_2_) and 3 µL Annexin V-FITC. After 30 min. of incubation at room temperature in the dark, 500 µL binding buffer and 10 µL PI solution (1 mg/mL) were added to the cell suspension before FACS analysis. FACS analysis was performed using a Gallios flow cytometer (Beckman Coulter, Brea, CA, USA) at 488 nm and data were collected from 1 × 10^4^ cells.

#### 3.2.7. Cell Cycle Assay

SW480 cells were seeded in 6-well plates and treated as described for the apoptosis assay. After centrifugation, the cells were resuspended in 500 µL DPBS and added dropwise to 4.5 mL 70% (*v*/*v*) cold ethanol. The cell suspension was kept at −20 °C for a minimum of 4 h. The cell suspension was then centrifuged, washed with cold DPBS, resuspended in 360 µL DPBS containing 10 µL RNase (10 mg/mL), and incubated for 1 h, at 37 °C, with agitation. Before FACS analysis, 20 µL PI solution (1 mg/mL) was added to the samples. FACS analysis was performed using a Gallios flow cytometer (Beckman Coulter, Brea, CA, USA) at 488 nm. Data were acquired from 1 × 10^4^ cells and subsequently analyzed using the multicycle software provided by Phoenix Flow Systems (San Diego, CA, USA).

#### 3.2.8. ROS Determination Assay

Intracellular ROS levels were assessed by flow cytometry using H_2_DCFDA probe. SW480 cells were cultured in 6-well plates and treated according to the procedure described for the apoptosis assay. After incubation for 24, 48, and 72 h, adherent cells were collected by centrifugation after trypsinization and washed with DPBS. The cells were then incubated with 1 mL HBSS containing 2 mM L-glutamine and 50 µM H_2_DCFDA probe at 37 °C for 45 min. in a cell culture incubator. Next, the cellular suspension was washed with 2 mL of DPBS and the cells were collected by centrifugation. Finally, the cells were gently resuspended in 350 µL DPBS containing 20 µg/mL PI solution (1 mg/mL). The intracellular internalized probe reacts with ROS and undergoes transformation into 2′,7′-dichlorofluorescein (DCF). DCF fluorescence data were collected from 1 × 10^4^ PI-negative cells by using a Gallios flow cytometer (Beckman Coulter, Brea, CA, USA) at 520 nm.

#### 3.2.9. Total Protein Extraction from Cultured Cells

SW480 cells (1.68 × 10^5^ cells/well) were seeded in 6-well plates and incubated at 37 °C for 24 h. Subsequently, the cells were incubated with fresh culture medium (control) or compound **14** at its IC_50_ value for 24, 48, and 72 h. At the end of the treatment period, cells were washed twice with ice-cold DPBS and incubated for 30 min. at 4 °C with lysis buffer containing 20 mM Tris-HCl (pH 7.5), 1 mM DTT, 0.2% Triton X-100, 1 mM Na-EDTA, 0.5 mM sodium deoxycholate, 1% proteases inhibitor cocktail, and 0.5% phosphatases inhibitor cocktail. The cells were scraped, sonicated, and centrifuged at 12,000× *g* for 20 min. at 4 °C. The supernatants were recovered, and the protein concentration was quantified using Pierce^TM^ BCA protein assay reagents A and B and Pierce^TM^ bovine serum albumin standard ampules (2 mg/mL).

#### 3.2.10. Western Blot Analysis

Equal amounts (5 µg) of protein were separated using 12% SDS–polyacrylamide gel electrophoresis (SDS–PAGE) and electroblotted onto PVDF membranes. Membranes were blocked with 5% skim milk in PBS-Tween-20 (0.1%) for 1 h at room temperature, following its incubation with the specific primary antibodies overnight at 4 °C (CDK4, CDK6, and SOD2 (MnSOD)). Exceptionally, the primary antibody against actin was only incubated for 30 min. at room temperature. The membranes were then washed with PBS-Tween-20 (0.1%) and incubated for 40 min. with an anti-mouse secondary antibody or for 1 h with an anti-rabbit secondary antibody. After incubation, the membranes were washed again with PBS-Tween-20 (0.1%) and treated with the Immobilon Western Chemiluminescent HRP Substrate Kit. The membranes were developed into autoradiographic films after proper exposure using an FPM-100A film processor (Fujifilm, Tokyo, Japan). Results were obtained from at least two independent experiments (CDK6: *n* = 3; CDK4 and SOD2 (MnSOD) at 24 and 48 h, *n* = 2; at 72 h, *n* = 3).

#### 3.2.11. Data Analysis and Statistical Methods

Each condition was performed in triplicate, and unless otherwise specified, the experiments were repeated a minimum of three times. Statistical analyses were conducted using GraphPad Prism 9 software (GraphPad Software, San Diego, CA, USA). Two-way ANOVA (Sidak’s post hoc comparison test) or multiple *t*-tests (one independent *t*-test per row) were used to compare the means between the control and treatment groups. All data are expressed as the mean ± SD. Differences were considered statistically significant at *p* < 0.05 (*), *p* < 0.01 (**), or *p* < 0.001 (***).

### 3.3. Molecular Docking Studies

#### 3.3.1. Protein and Ligand Preparation

Twelve experimental 3D structures of CDK6, identified by PDB IDs 1XO2, 2EUF, 2F2C, 3NUP, 3NUX, 4AUA, 4EZ5, 4TTH, 5L2I, 6OQL, 6OQO, and 8I0M, were obtained from the Protein Data Bank (PDB) to select the most suitable protein structure for docking calculations. Each structure was prepared using Molecular Operating Environment (MOE) (version 2024) [103] and the QuickPrep tool was used to remove water molecules, co-crystallized ligands, and ions. The 3D coordinates of CDK6 structures were then aligned, and a docking box with specific dimensions (15.0 Å) centered at the coordinates (x = −1.09, y = 36.82, z = 139.83) was defined. All the crystallographic ligands from the 12 CDK6 complexes and compound **14** were prepared using MOE, protonated, and minimized using the Amber10 force field [104,105,106].

#### 3.3.2. Docking Protocol Validation and Simulations

Two software packages, GNINA 1.0 [107,108] and SMINA 1.0 [109], were used for the docking validation protocol. Self-docking and cross-docking were performed to assess which combination of CDK6 crystal structure and software best reproduced the pose of the co-crystallized ligands docked into each respective crystal structure (self-docking) and each crystal structure and other ligands (cross-docking). RMSDs were calculated between the poses of the crystallographic ligands and docking results. RMSD lower than 2 Å is widely accepted as corresponding to good docking solutions [110]. A Python script was also developed for these calculations. PDB structure 6OQL and the software GNINA 1.0 demonstrated the best combined performance and were selected for docking simulations of compound **14**. The docking results were analyzed using the Protein–Ligand Interaction Profiler (PLIP) to assess the molecular interactions between the protein and ligand [111].

## 4. Conclusions

In this study, we designed and synthesized a panel of new derivatives of CA **1** by inserting a urea moiety at the C-20 position. Most urea-containing CA **1** derivatives showed better antiproliferative activity than that of the parent compound. Through SAR, it was possible to conclude that longer aliphatic chains, insertion of electronegative atoms, and aromatic rings on the urea moieties favored the antiproliferative activity of the derivatives. Compound **14**, which possesses a benzene ring substituted with a -Cl atom, was the most potent against all tested cancer cell lines (CRC, melanoma, and pancreatic cancer). Among the CRC cell lines, this derivative showed the best antiproliferative activity against p53-mutated and microsatellite stable cell lines, particularly SW480 and SW620 cells. Furthermore, selectivity studies revealed that compound **14** exhibited greater specificity against cancer cell lines than against normal cells. Compound **14** induced a slower rate of cell growth and increased the cell doubling time from 42 h (control) to 70 h (treatment group), confirming its antiproliferative effect. Preliminary mechanistic studies showed that compound **14** arrested the cell cycle at the G0/G1 phase by downregulating CDK4 and CDK6 in SW480 cells. Moreover, molecular docking studies indicated that compound **14** could potentially inhibit CDK6 as it established interactions with active site residues of CDK6. Thus, this kinase could be a potential therapeutic target for compound **14**. Compound **14** also displayed an antioxidant effect in SW480 cells. In conclusion, our study suggests that compound **14** is a promising lead for the development of new anticancer drugs, and merits further investigation.

## Data Availability

The original contributions presented in this study are included in the article/Supplementary Materials; further inquiries can be directed to the corresponding authors.

## Supplementary Materials

The following supporting information can be downloaded at: https://www.mdpi.com/article/10.3390/ijms252413332/s1.
