# Supplementary material for: Design, Synthesis, and Biological Evaluation of Novel Urea-Containing Carnosic Acid Derivatives with Anticancer Activity
Source: Int J Mol Sci. 2024 Dec 12;25(24):13332. doi: 10.3390/ijms252413332 (PMC11728001; doi:10.3390/ijms252413332)
Supplement: Supplementary file 1 [file ijms-25-13332-s001.zip › ijms-3270314-supplementary.pdf]

Supplementary Materials

Design, synthesis, and biological evaluation of novel urea-containing carnosic acid derivatives with anticancer activity

Sara P.S.P. Moura [1, 2], Silvia Marín [3, 4], Ismael Rufino[5], Rita C. Guedes[5], Marta Cascante [3, 4,*] and Jorge A.R. Salvador [1, 2,*]

[1] *Laboratory of Pharmaceutical Chemistry, Faculty of Pharmacy, University of Coimbra, 3000-548 Coimbra, Portugal; spmoura17@gmail.com*

[2] *Center for Neuroscience and Cell Biology (CNC), University of Coimbra, 3004-504 Coimbra, Portugal*

[3] *Department of Biochemistry and Molecular Biomedicine, Faculty of Biology, University of Barcelona, 08028 Barcelona, Spain; silviamarin@ub.edu*

[4] *Centro de Investigación Biomédica en Red de Enfermedades Hepáticas y Digestivas (CIBEREHD), Instituto de Salud Carlos III (ISCIII), 28029 Madrid, Spain*

[5] *Research Institute for Medicines (iMed.ULisboa), Faculty of Pharmacy, University of Lisboa, 1649-003 Lisboa, Portugal; ismaelcarvalho@edu.ulisboa.pt (I.R.); rguedes@ff.ulisboa.pt (R.C.G.)*

** Correspondence: salvador@ci.uc.pt (J.A.R.S); martacascante@ub.edu (M.C.); Tel.: +351-239-488-479 (J.A.R.S; +34-934-021-593 (M.C.)*

Table of Contents

1. NMR data for compound 2 5

1.1. ^{1}H spectrum for compound **2** recorded in $CDCl_3$ 5

1.2. ^{13}C spectrum for compound **2** recorded in $CDCl_3$ 6

2. NMR data for compound 5 7

2.1. ^{1}H spectrum for compound **5** recorded in $CDCl_3$ 7

2.2. ^{13}C spectrum for compound **5** recorded in $CDCl_3$ 8

3. NMR data for compound 6 9

3.1. ^{1}H spectrum for compound **6** recorded in $CDCl_3$ 9

3.2. ^{13}C spectrum for compound **6** recorded in $CDCl_3$ 10

4. NMR data for compound 7 11

4.1. ^{1}H spectrum for compound **7** recorded in $CDCl_3$ 11

4.2. ^{13}C spectrum for compound **7** recorded in $CDCl_3$ 12

5. NMR data for compound 8 13

5.1. ^{1}H spectrum for compound **8** recorded in $CDCl_3$ 13

5.2. ^{13}C spectrum for compound **8** recorded in $CDCl_3$ 14

6. NMR data for compound 9 15

6.1. ^{1}H spectrum for compound **9** recorded in $CDCl_3$ 15

6.2. ^{13}C spectrum for compound **9** recorded in $CDCl_3$ 16

7. NMR data for compound 10 17

7.1. ^{1}H spectrum for compound **10** recorded in $CDCl_3$ 17

7.2. ^{13}C spectrum for compound **10** recorded in $CDCl_3$ 18

8. NMR data for compound 11 19

8.1. ^{1}H spectrum for compound **11** recorded in $CDCl_3$ 19

8.2. ^{13}C spectrum for compound **11** recorded in $CDCl_3$ 20

8.3. COSY spectrum for compound **11** recorded in $CDCl_3$ 21

8.4. NOESY spectrum for compound **11** recorded in $CDCl_3$ 22

8.5. HSQC spectrum for compound **11** recorded in $CDCl_3$ 23

8.6. HMBC spectrum for compound **11** recorded in $CDCl_3$ 24

9. NMR data for compound 12 25

9.1. ^{1}H spectrum for compound **12** recorded in $CDCl_3$ 25

9.2. ^{13}C spectrum for compound **12** recorded in $CDCl_3$ 26
9.3. COSY spectrum for compound **12** recorded in $CDCl_3$ 27
9.4. NOESY spectrum for compound **12** recorded in $CDCl_3$ 28
9.5. HSQC spectrum for compound **12** recorded in $CDCl_3$ 29
9.6. HMBC spectrum for compound **12** recorded in $CDCl_3$ 30
10. NMR data for compound 13 31
10.1. ^{1}H spectrum for compound **13** recorded in $CDCl_3$ 31
10.2. ^{13}C spectrum for compound **13** recorded in $CDCl_3$ 32
11. NMR data for compound 14 33
11.1. ^{1}H spectrum for compound **14** recorded in $CDCl_3$ 33
11.2. ^{13}C spectrum for compound **14** recorded in $CDCl_3$ 34
11.3. COSY spectrum for compound **14** recorded in $CDCl_3$ 35
11.4. NOESY spectrum for compound **14** recorded in $CDCl_3$ 36
11.5. HSQC spectrum for compound **14** recorded in $CDCl_3$ 37
11.6. HMBC spectrum for compound **14** recorded in $CDCl_3$ 38
12. NMR data for compound 15 39
12.1. ^{1}H spectrum for compound **15** recorded in $CDCl_3$ 39
12.2. ^{13}C spectrum for compound **15** recorded in $CDCl_3$ 40
13. NMR data for compound 16 41
13.1. ^{1}H spectrum for compound **16** recorded in $CDCl_3$ 41
13.2. ^{13}C spectrum for compound **16** recorded in $CDCl_3$ 42
13.3. COSY spectrum for compound **16** recorded in $CDCl_3$ 43
13.4. HSQC spectrum for compound **16** recorded in $CDCl_3$ 44
13.5. HMBC spectrum for compound **16** recorded in $CDCl_3$ 45
14. NMR data for compound 17 46
14.1. ^{1}H spectrum for compound **17** recorded in $CDCl_3$ 46
14.2. ^{13}C spectrum for compound **17** recorded in $CDCl_3$ 47
15. NMR data for compound 18 48
15.1. ^{1}H spectrum for compound **18** recorded in $CDCl_3$ 48
15.2. ^{13}C spectrum for compound **18** recorded in $CDCl_3$ 49
15.3. HSQC spectrum for compound **18** recorded in $CDCl_3$ 50

16. NMR data for compound 19 51
16.1. ^{1}H spectrum for compound **19** recorded in $CDCl_3$.. 51
16.2. ^{13}C spectrum for compound **19** recorded in $CDCl_3$... 52
16.3. HSQC spectrum for compound **19** recorded in $CDCl_3$.. 53
17. NMR data for compound 20 54
17.1. ^{1}H spectrum for compound **20** recorded in $CDCl_3$.. 54
17.2. ^{13}C spectrum for compound **20** recorded in $CDCl_3$ 55
17.3. COSY spectrum for compound **20** recorded in $CDCl_3$.. 56
17.4. HSQC spectrum for compound **20** recorded in $CDCl_3$.. 57
17.5. HMBC spectrum for compound **20** recorded in $CDCl_3$... 58
18. Uncropped and unprocessed western blot gels.. 59

1. NMR data for compound 2

*1.1. ^{1}H spectrum for compound **2** recorded in $CDCl_3$*

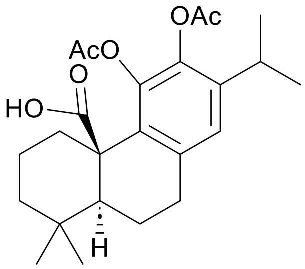

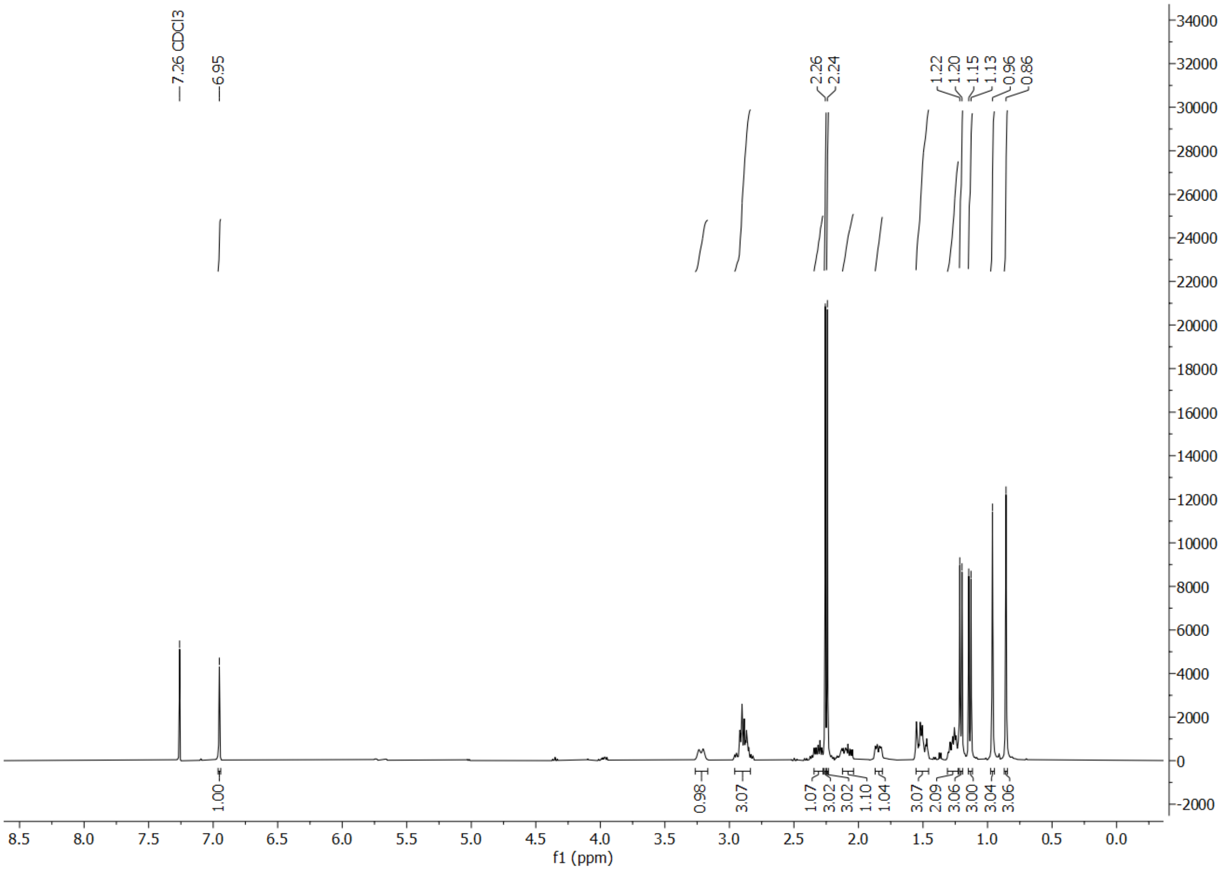

*1.2. ^{13}C spectrum for compound **2** recorded in $CDCl_3$*

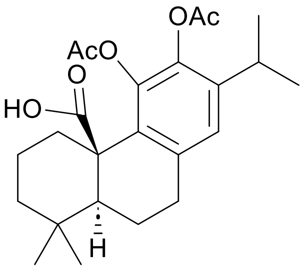

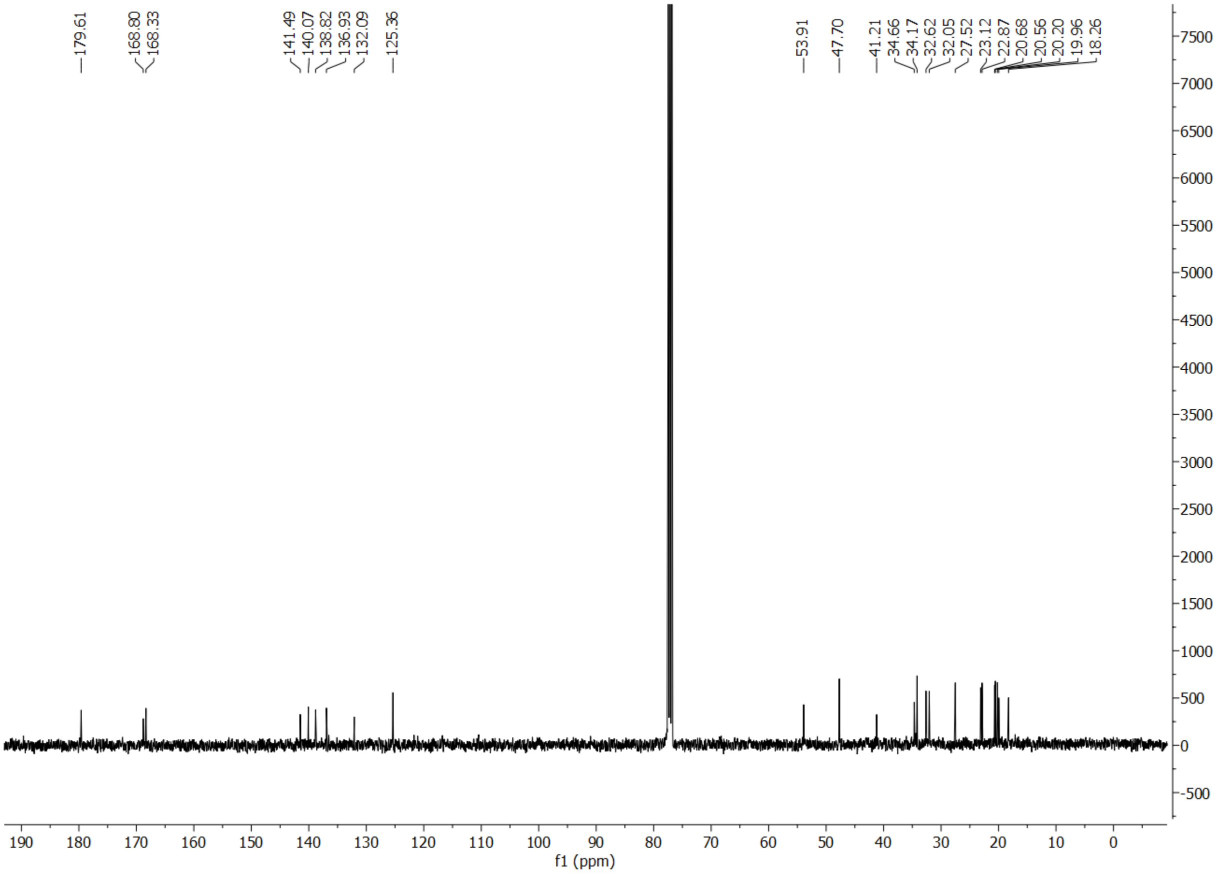

2. NMR data for compound 5

*2.1. ^{1}H spectrum for compound **5** recorded in $CDCl_3$*

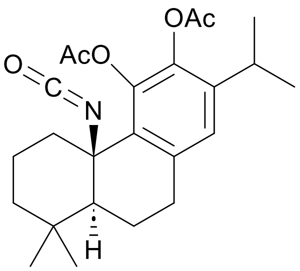

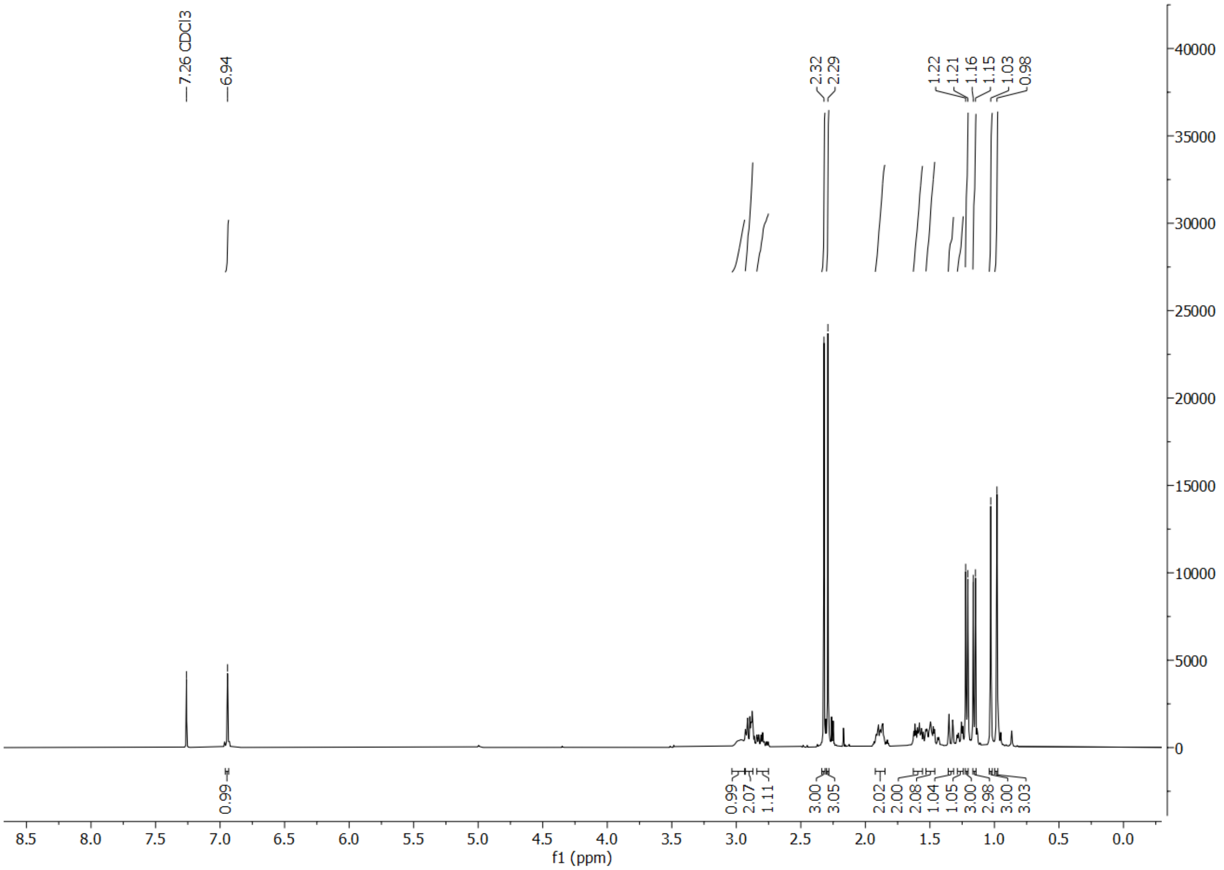

*2.2. ^{13}C spectrum for compound **5** recorded in $CDCl_3$*

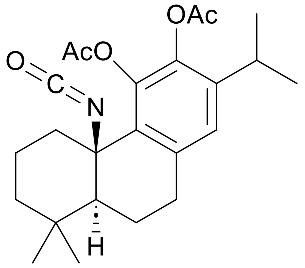

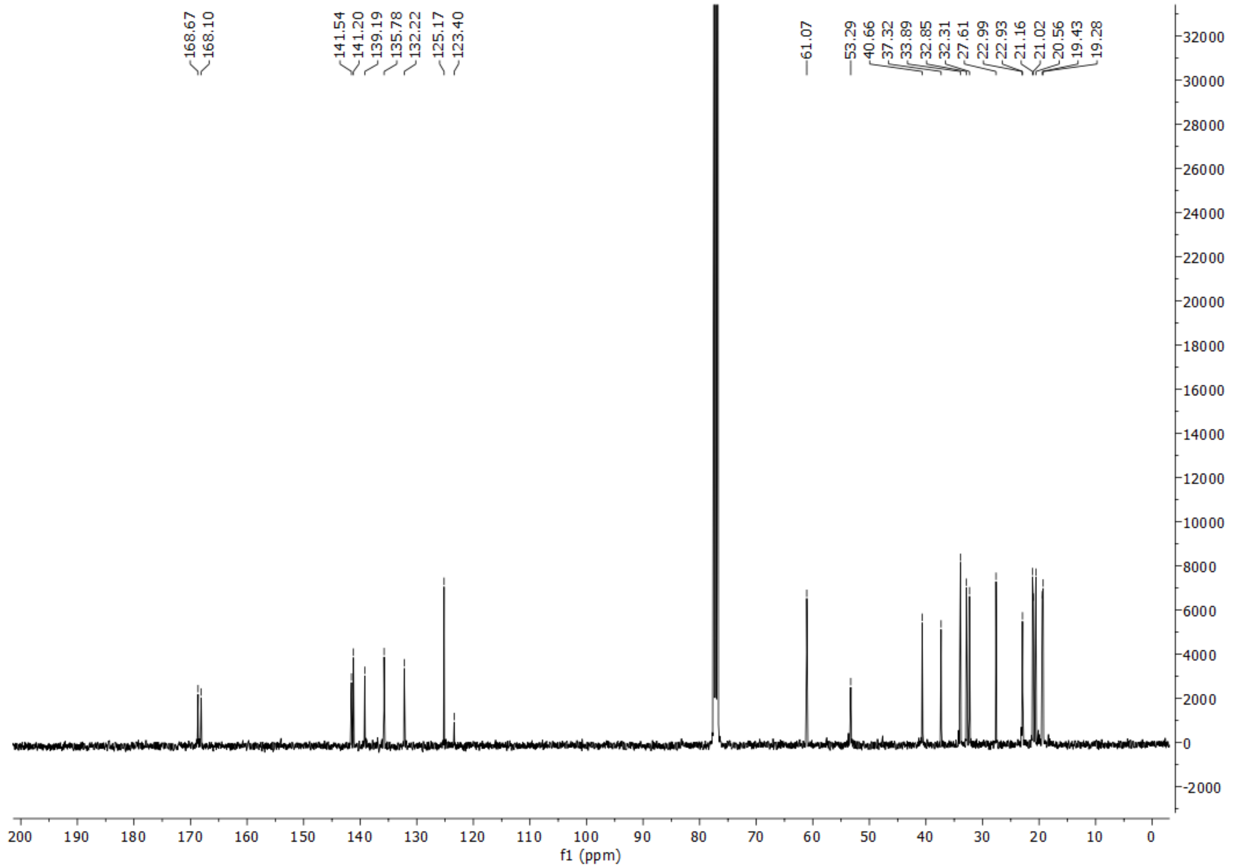

3. NMR data for compound 6

*3.1. ^1H spectrum for compound **6** recorded in $CDCl_3$*

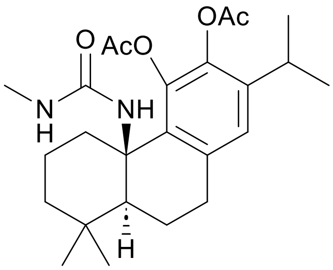

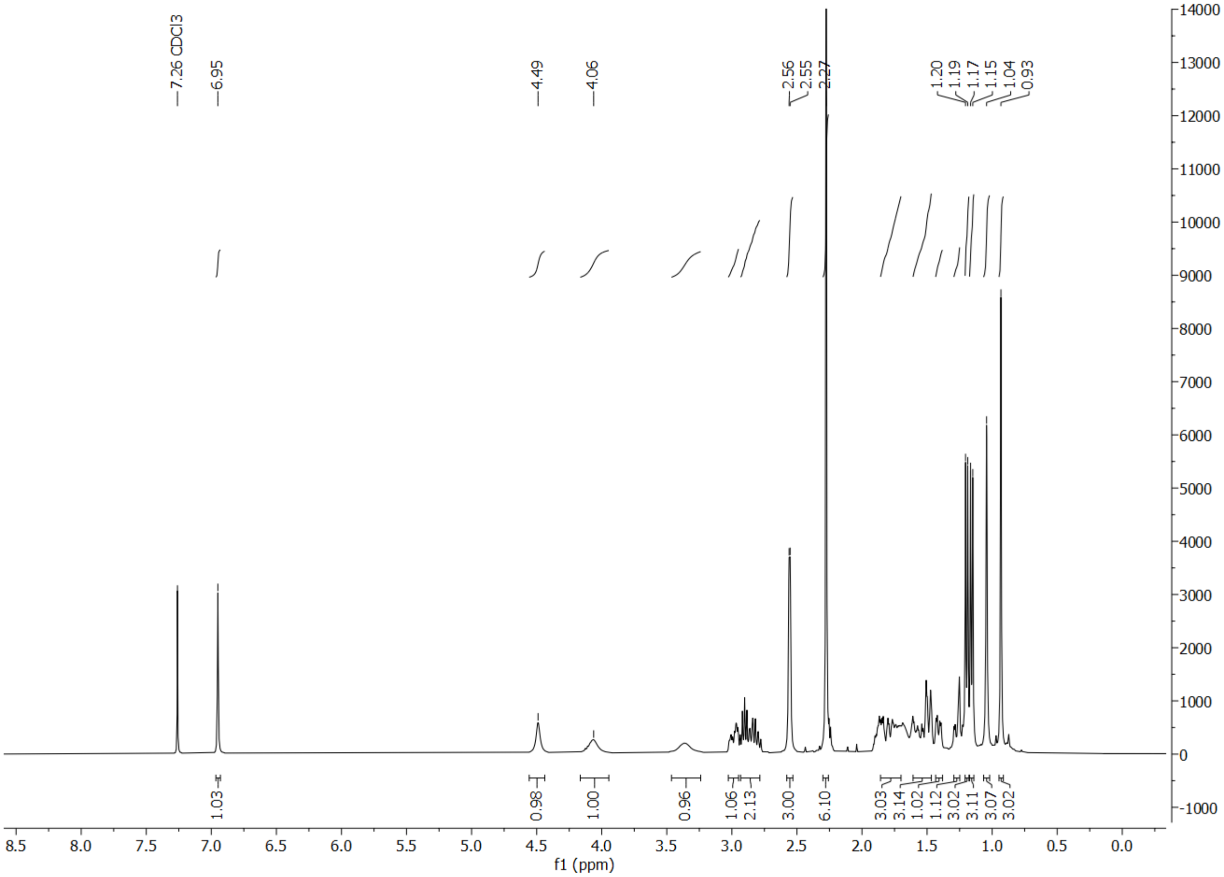

*3.2. ^{13}C spectrum for compound **6** recorded in $CDCl_3$*

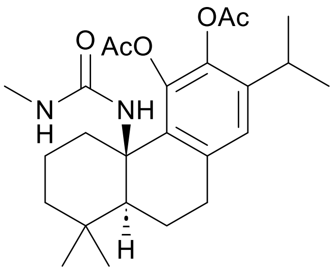

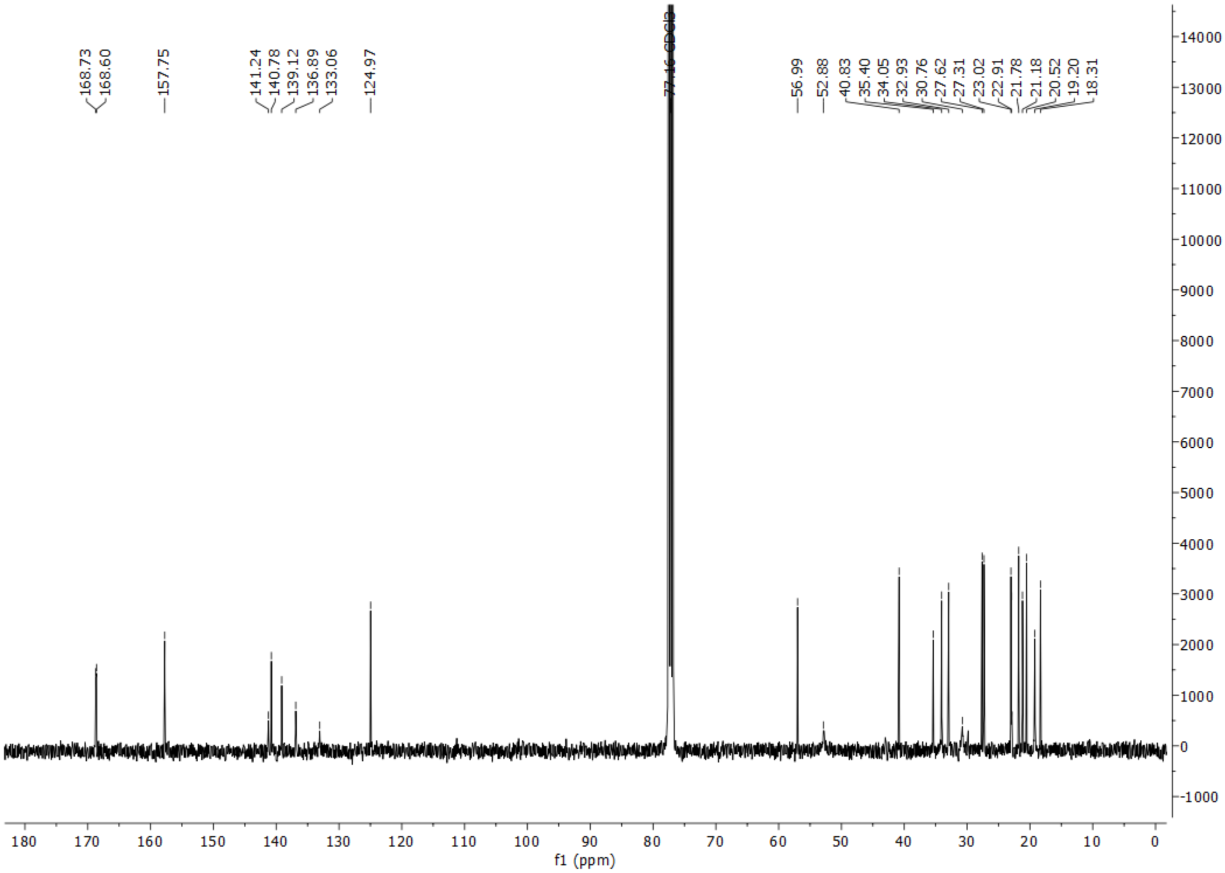

4. NMR data for compound 7

*4.1. ^{1}H spectrum for compound **7** recorded in $CDCl_3$*

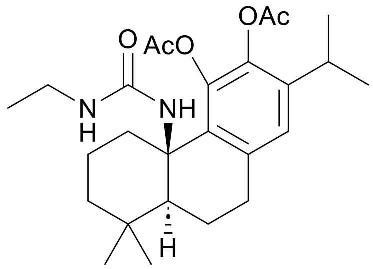

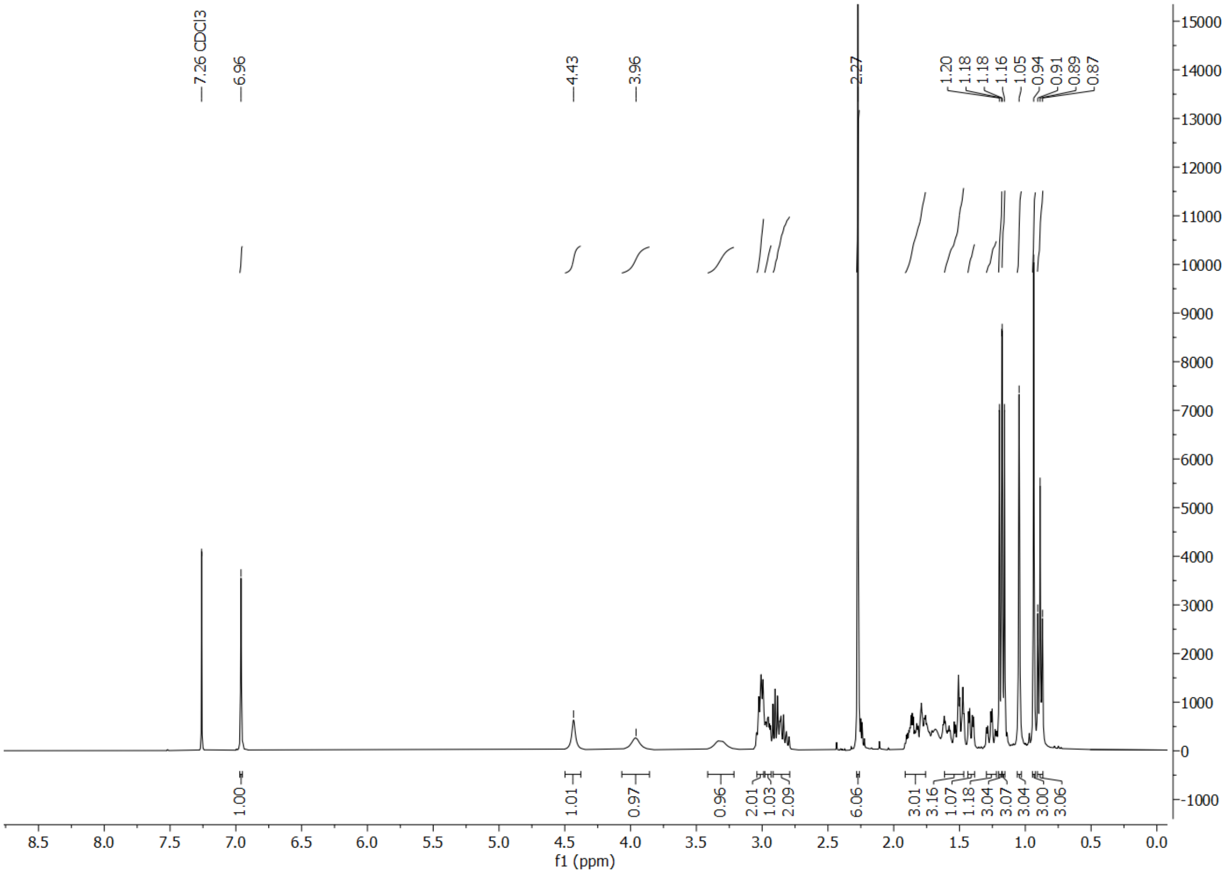

*4.2. ^{13}C spectrum for compound **7** recorded in $CDCl_3$*

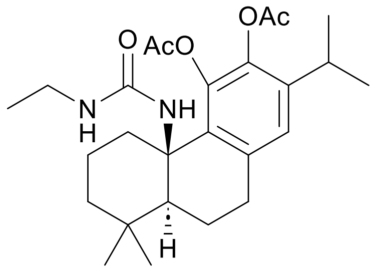

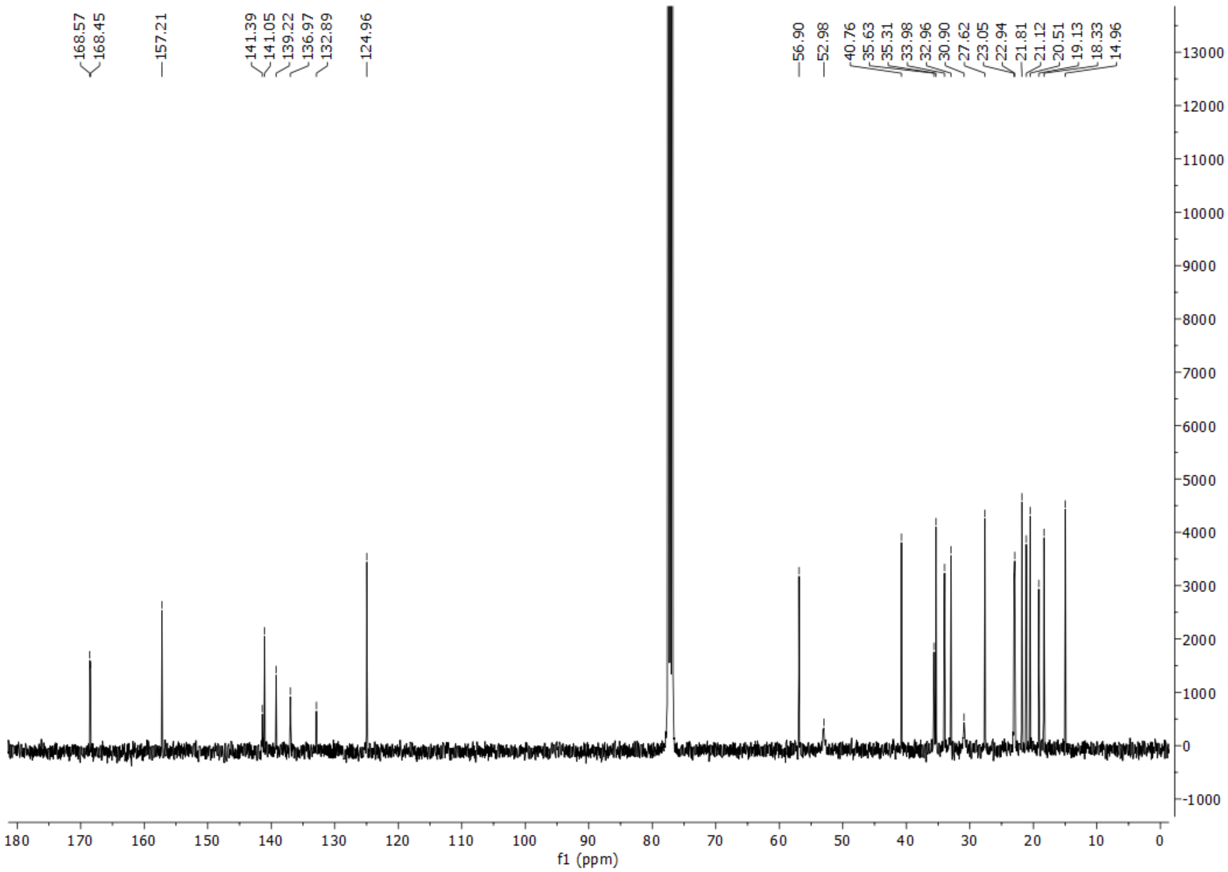

5. NMR data for compound 8

*5.1. ^{1}H spectrum for compound **8** recorded in $CDCl_3$*

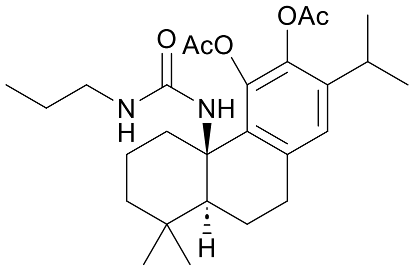

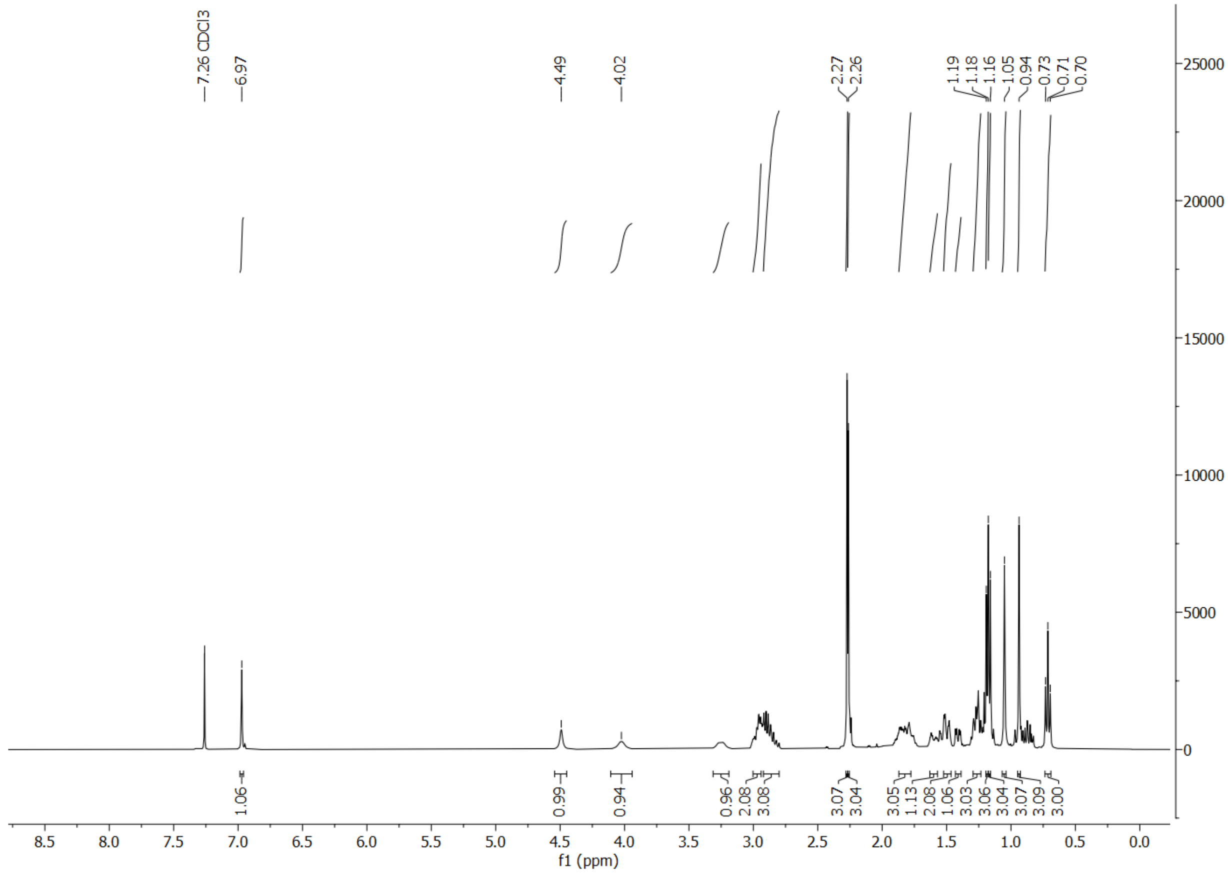

5.2. *^{13}C spectrum for compound **8** recorded in $CDCl_3$*

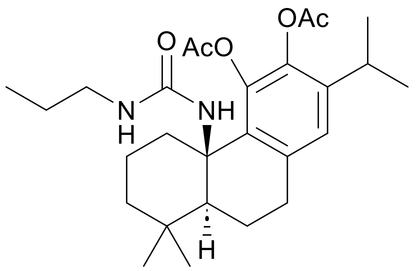

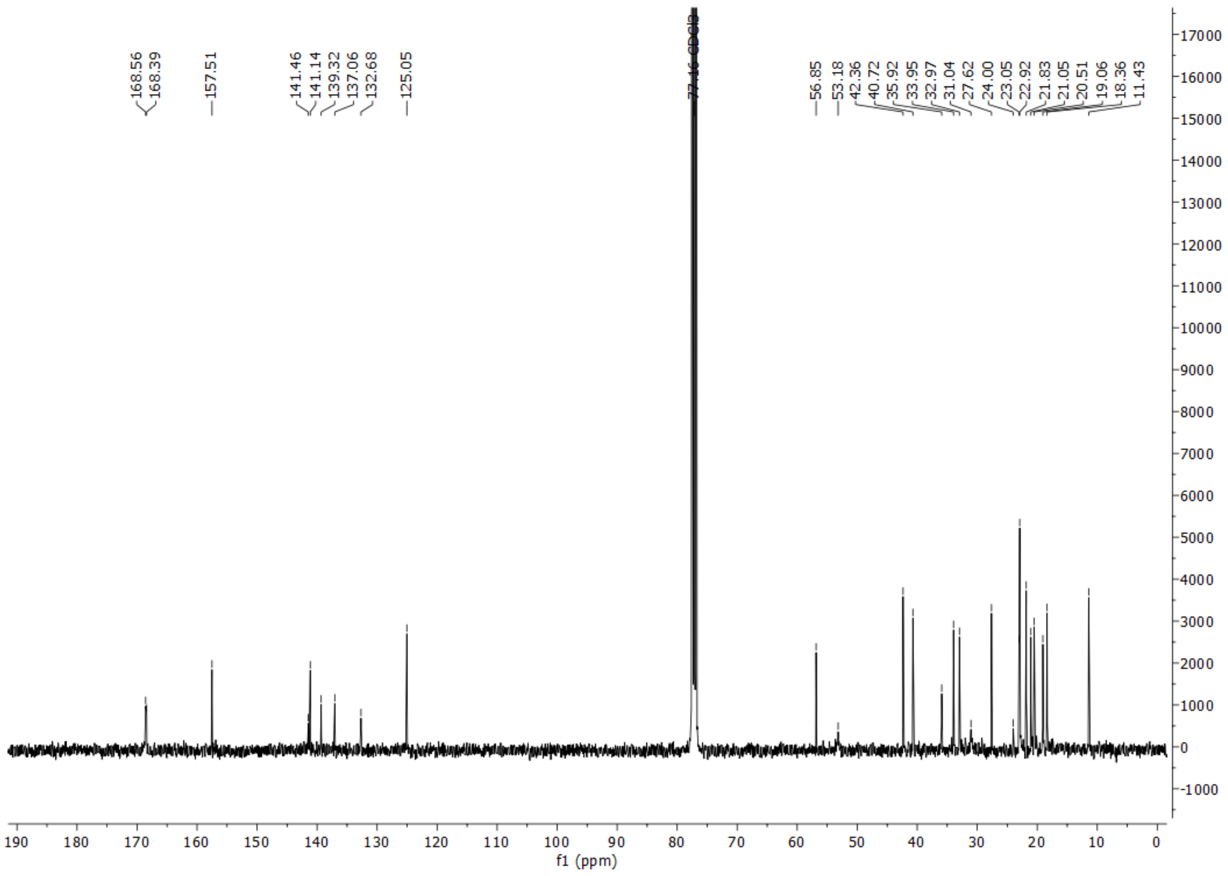

6. NMR data for compound 9

*6.1. ^{1}H spectrum for compound **9** recorded in $CDCl_3$*

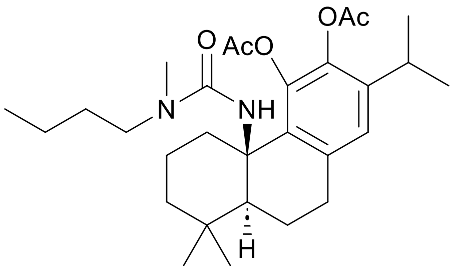

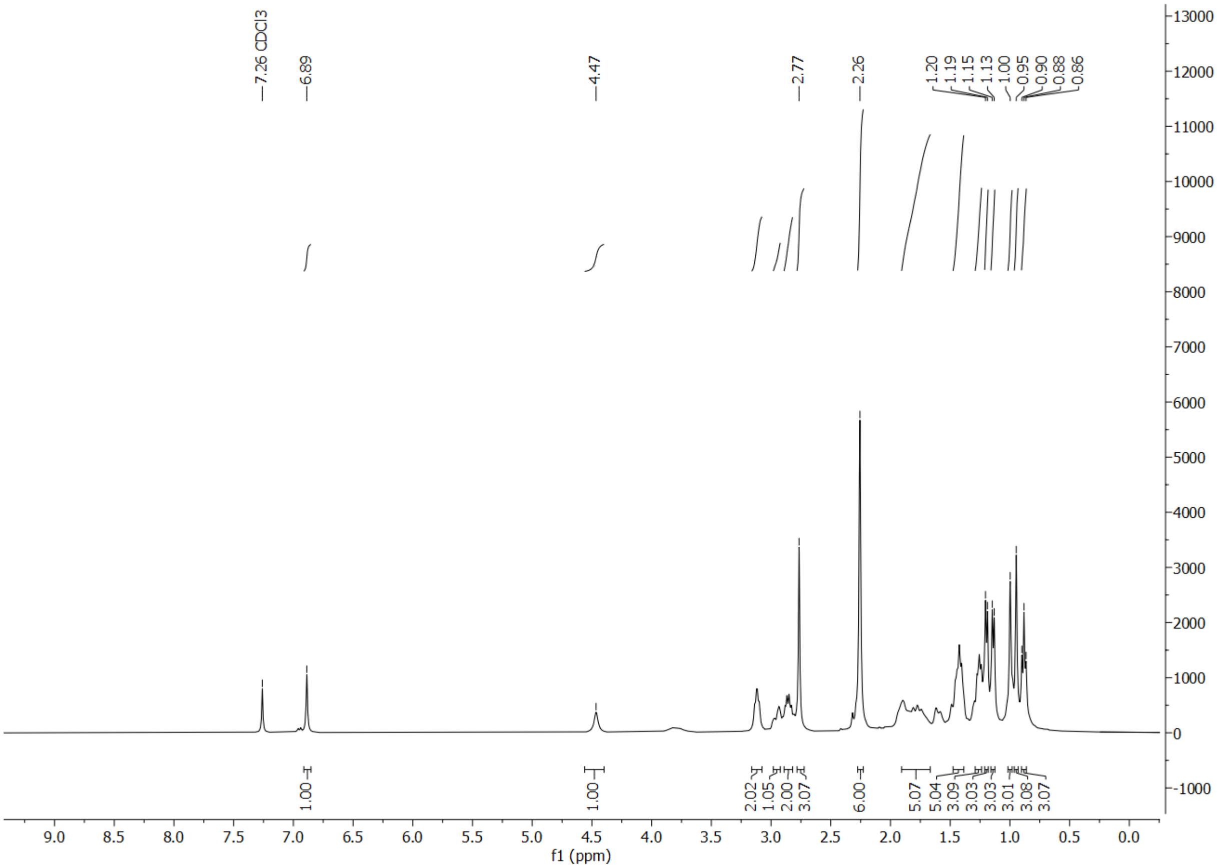

*6.2. ^{13}C spectrum for compound **9** recorded in $CDCl_3$*

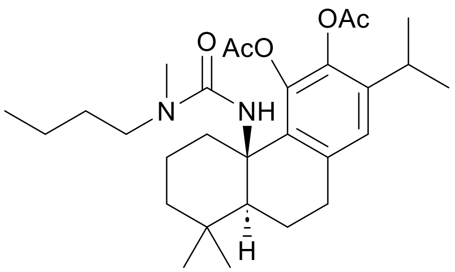

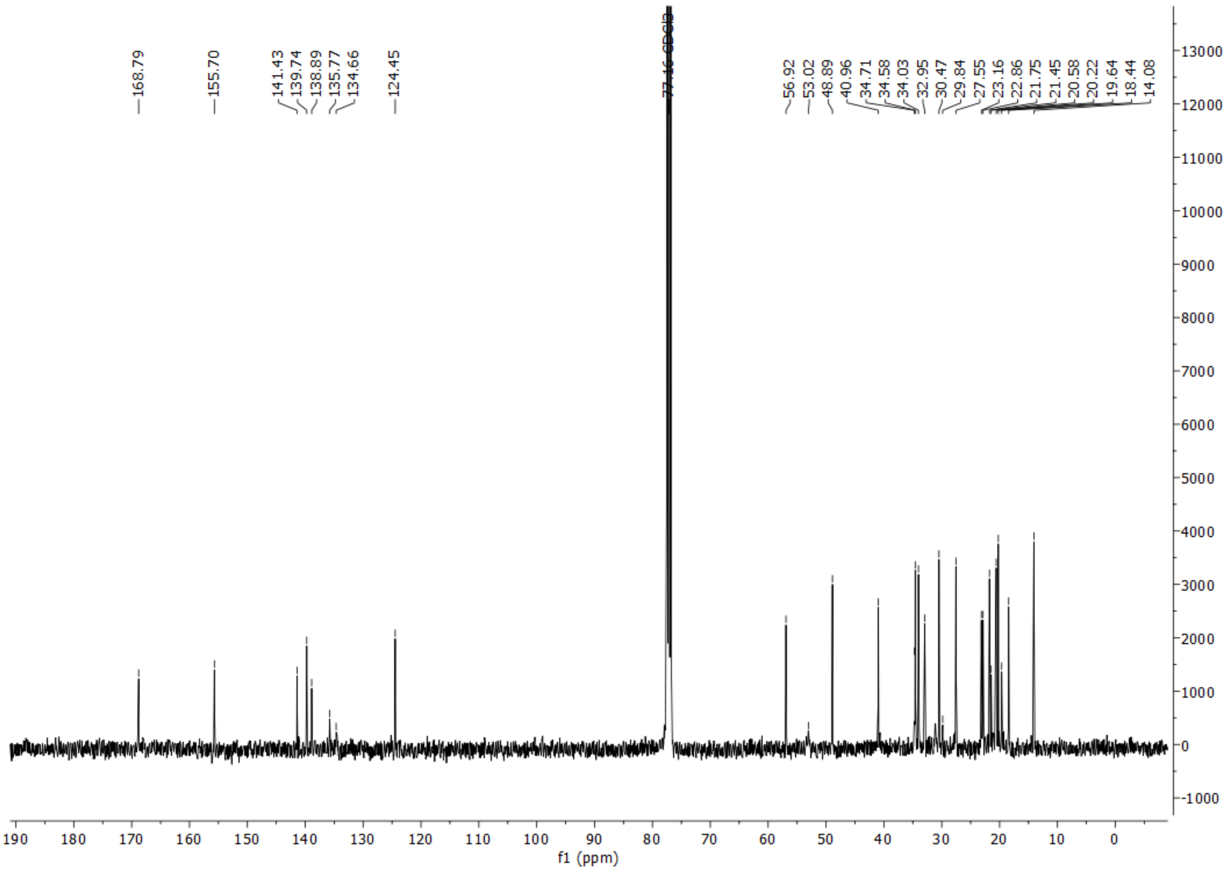

7. NMR data for compound 10

*7.1. ¹H spectrum for compound **10** recorded in $CDCl_3$*

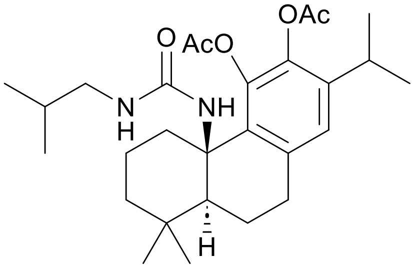

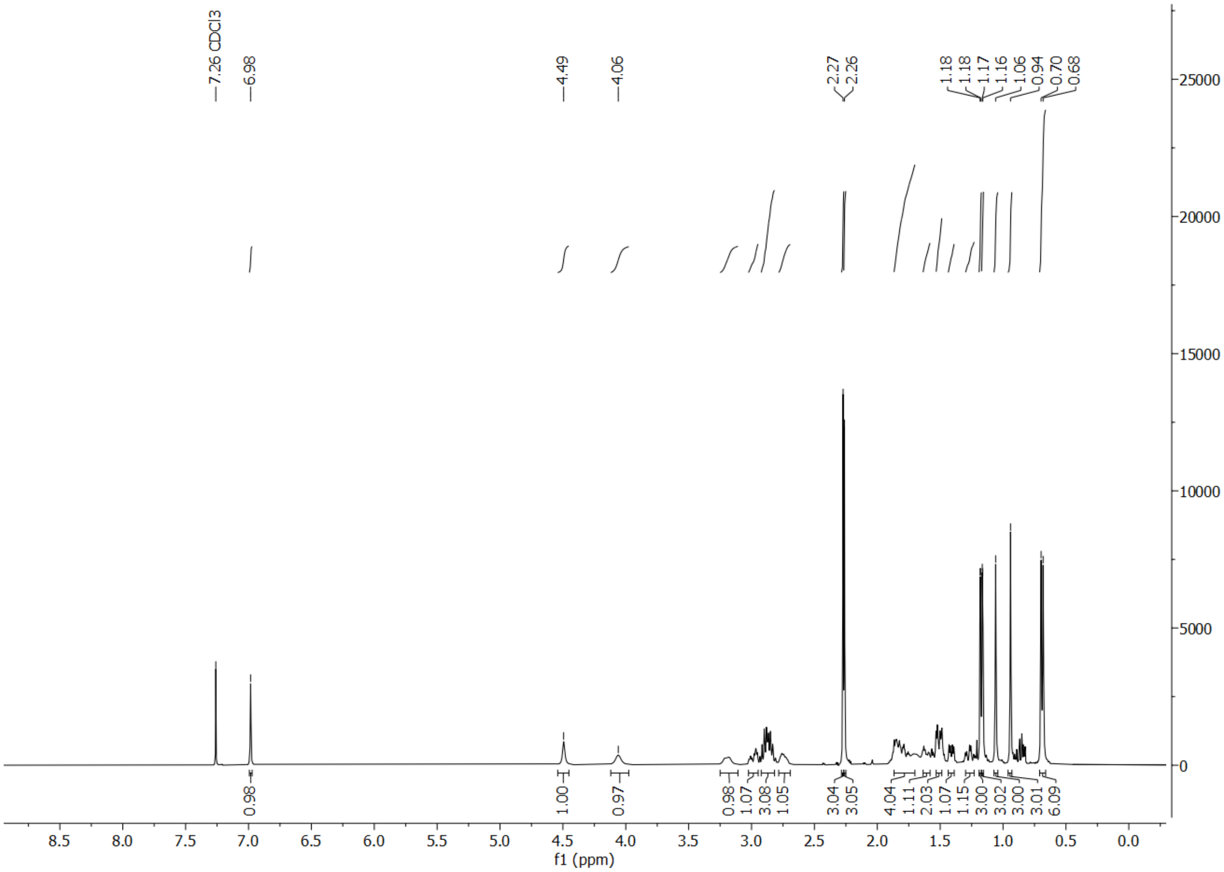

*7.2. ^{13}C spectrum for compound **10** recorded in $CDCl_3$*

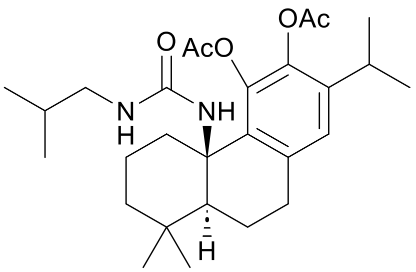

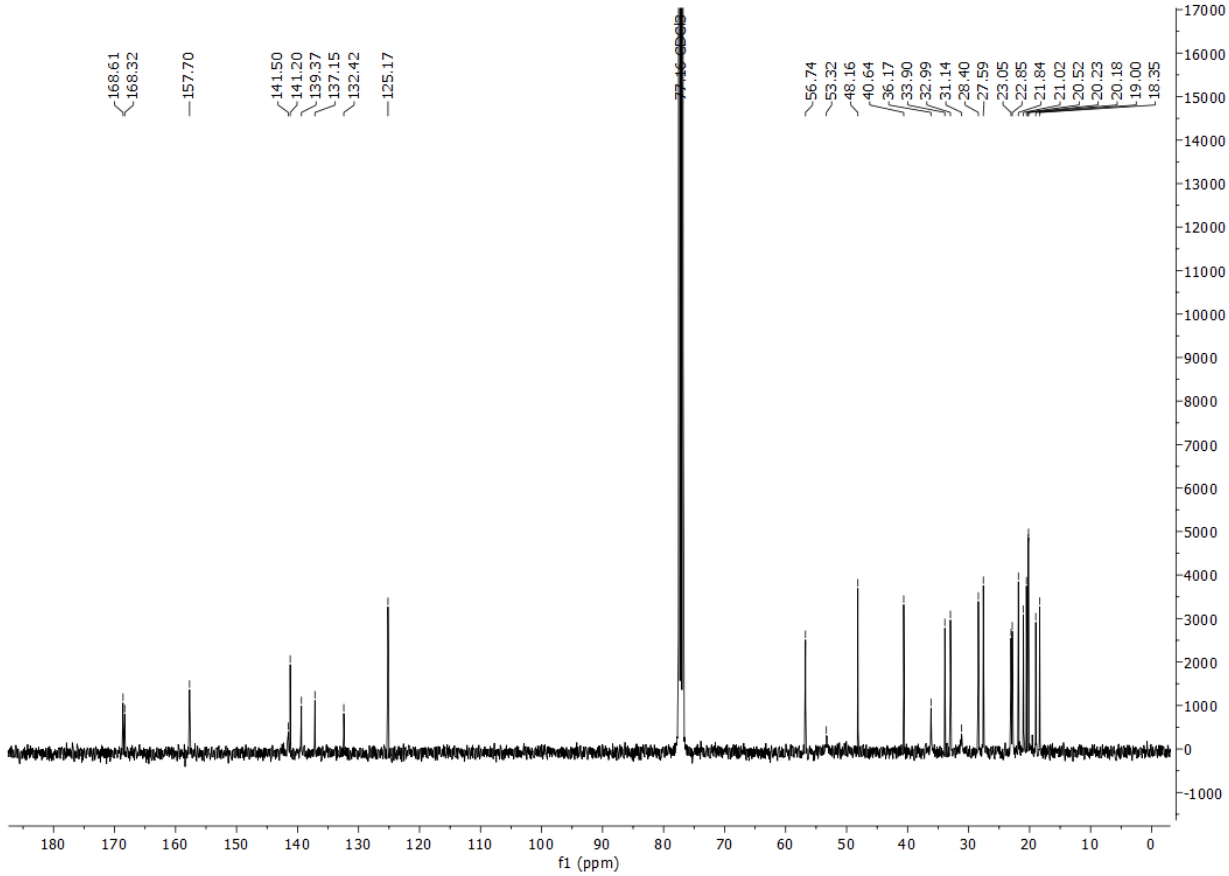

8. NMR data for compound 11

*8.1. ^{1}H spectrum for compound **11** recorded in $CDCl_3$*

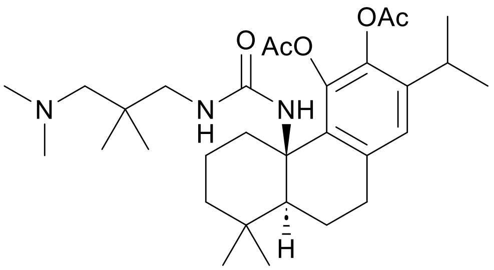

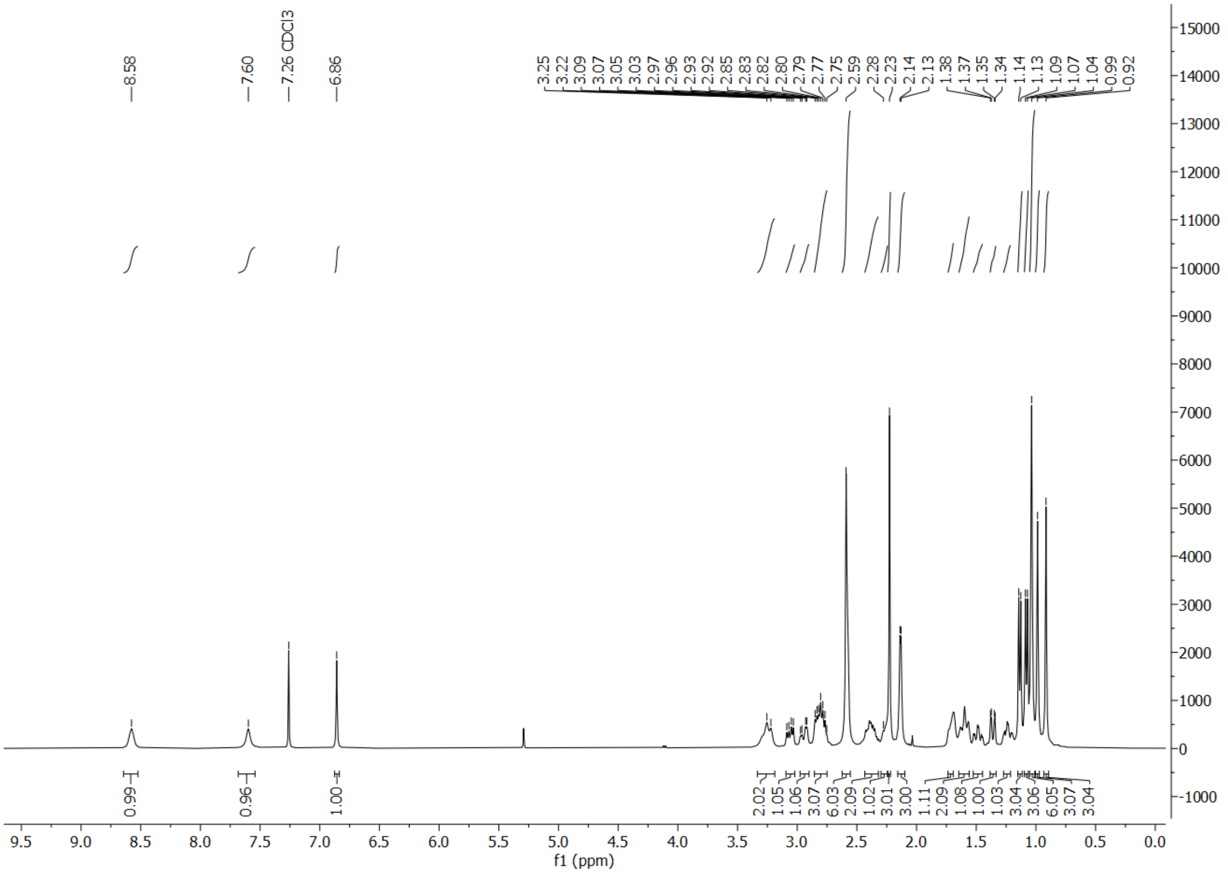

*8.2. ${}^{13}C$ spectrum for compound **11** recorded in $CDCl_3$*

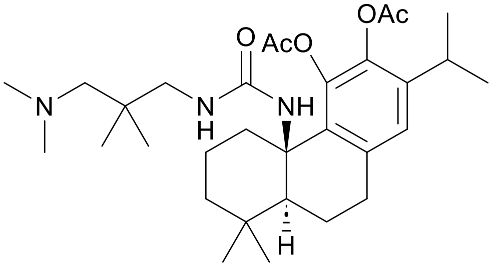

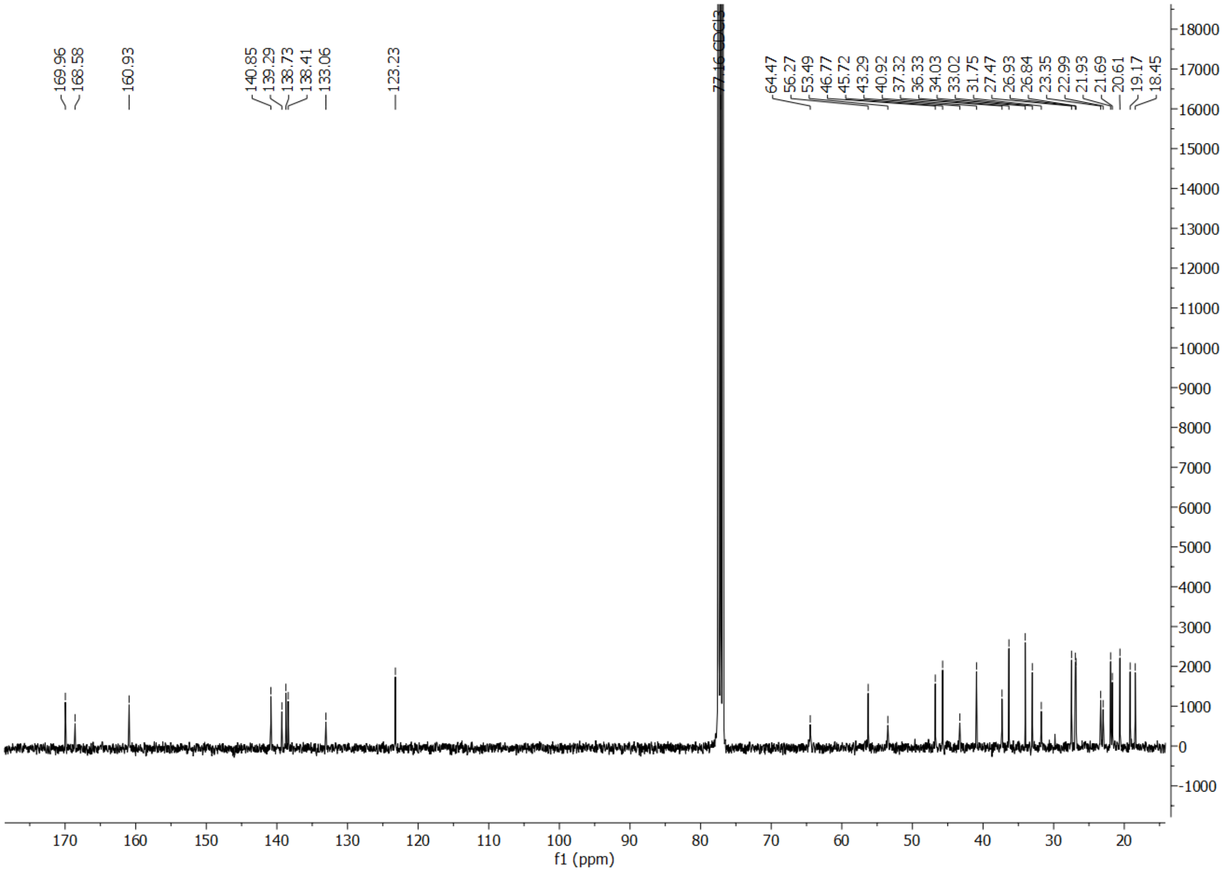

*8.3. COSY spectrum for compound **11** recorded in $CDCl_3$*

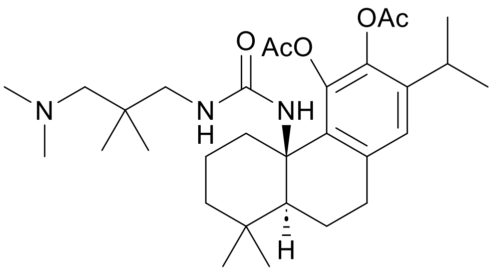

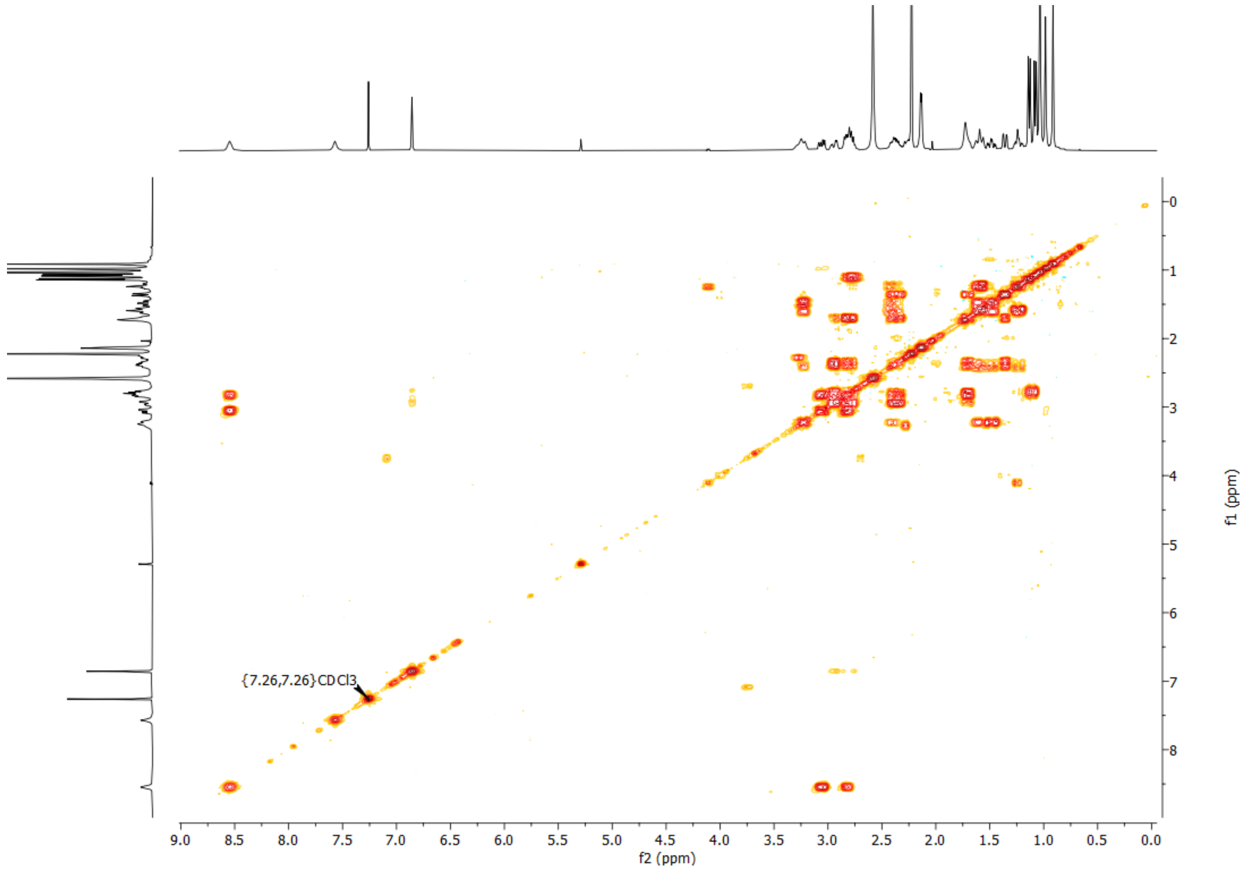

*8.4. NOESY spectrum for compound **11** recorded in $CDCl_3$*

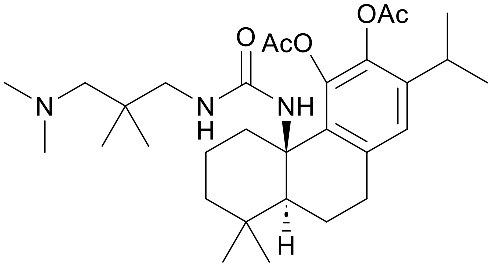

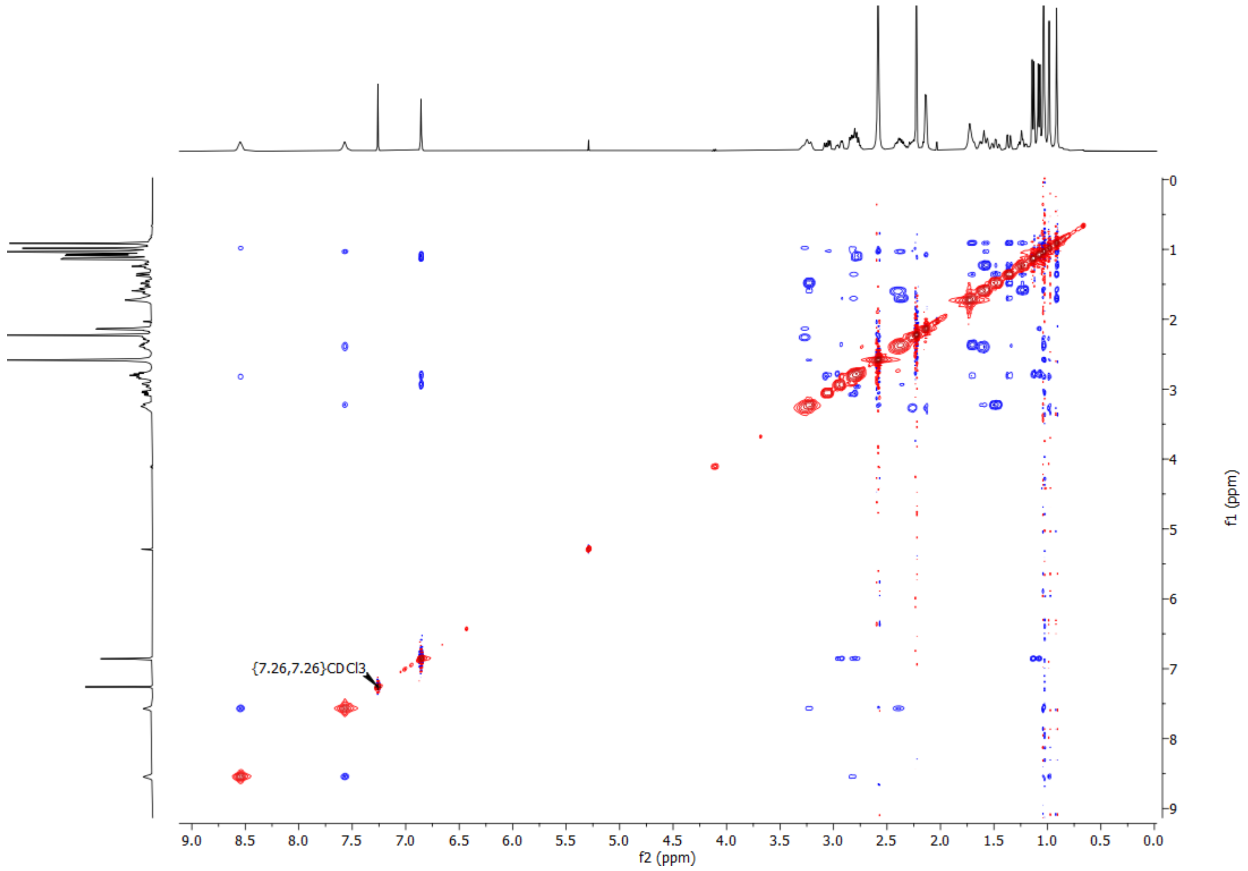

*8.5. HSQC spectrum for compound **11** recorded in $CDCl_3$*

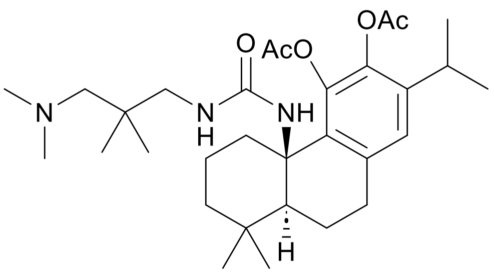

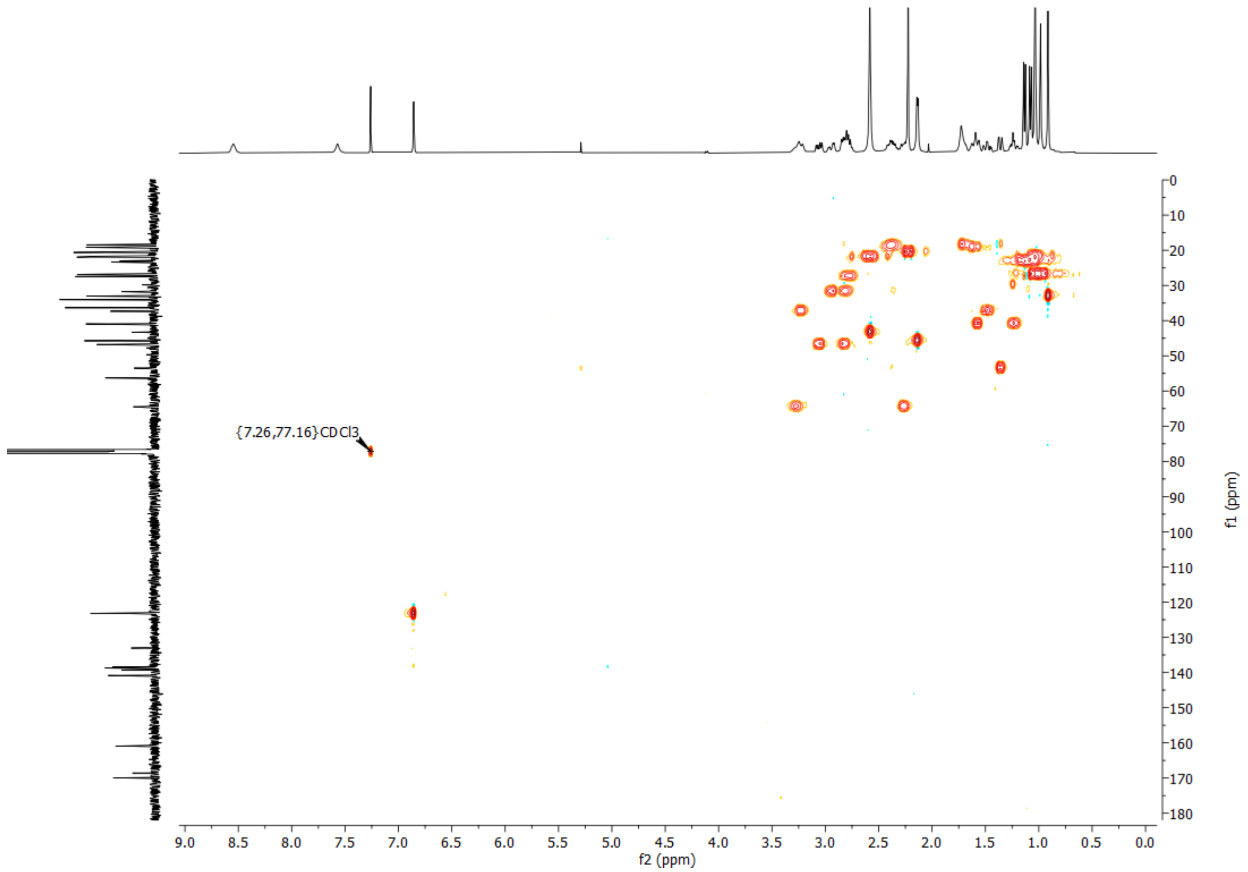

*8.6. HMBC spectrum for compound **11** recorded in $CDCl_3$*

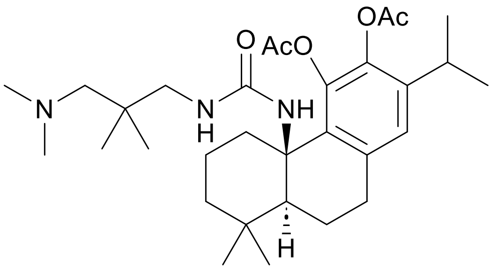

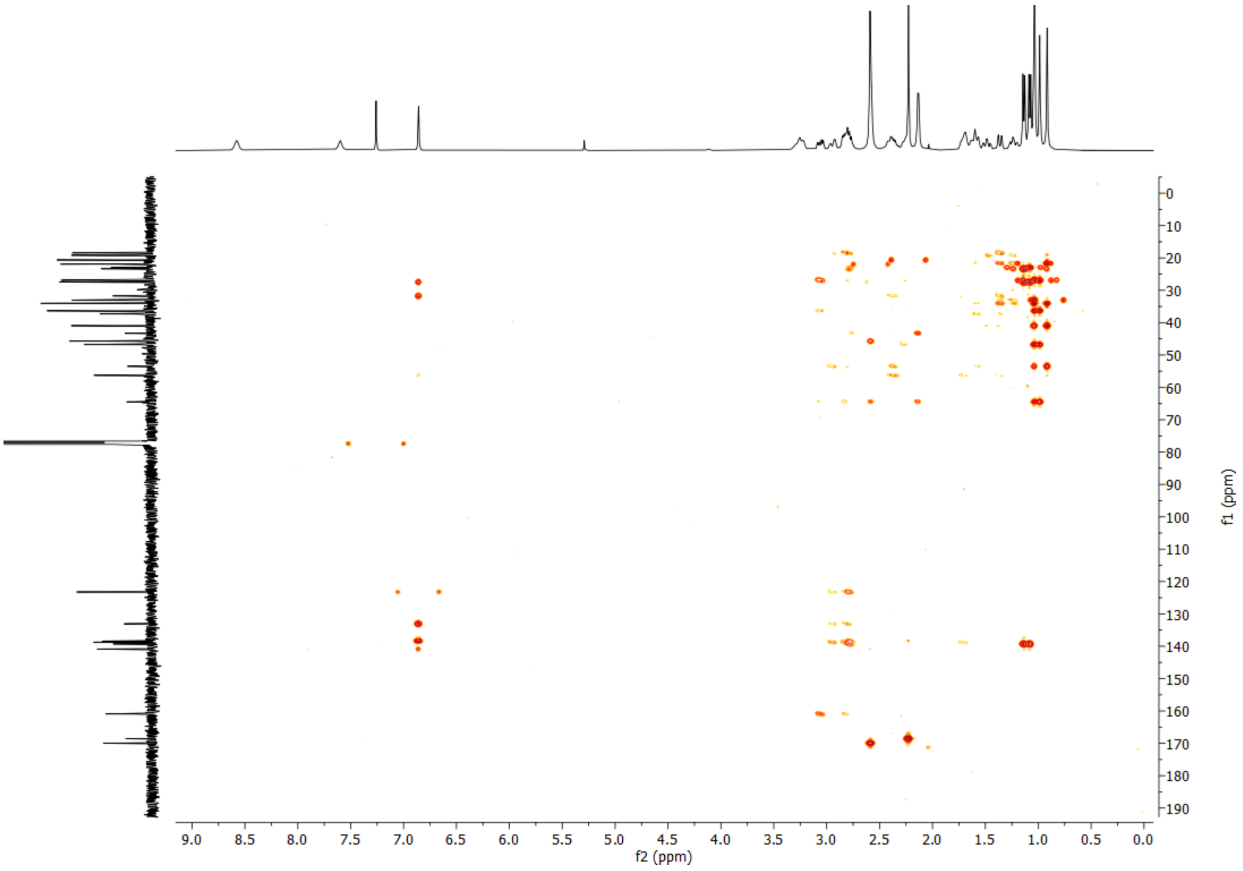

9. NMR data for compound 12

*9.1. ^{1}H spectrum for compound **12** recorded in $CDCl_3$*

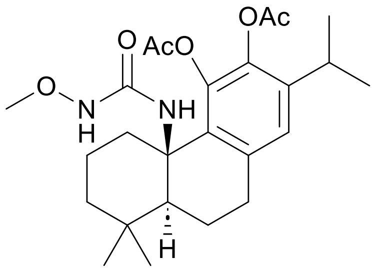

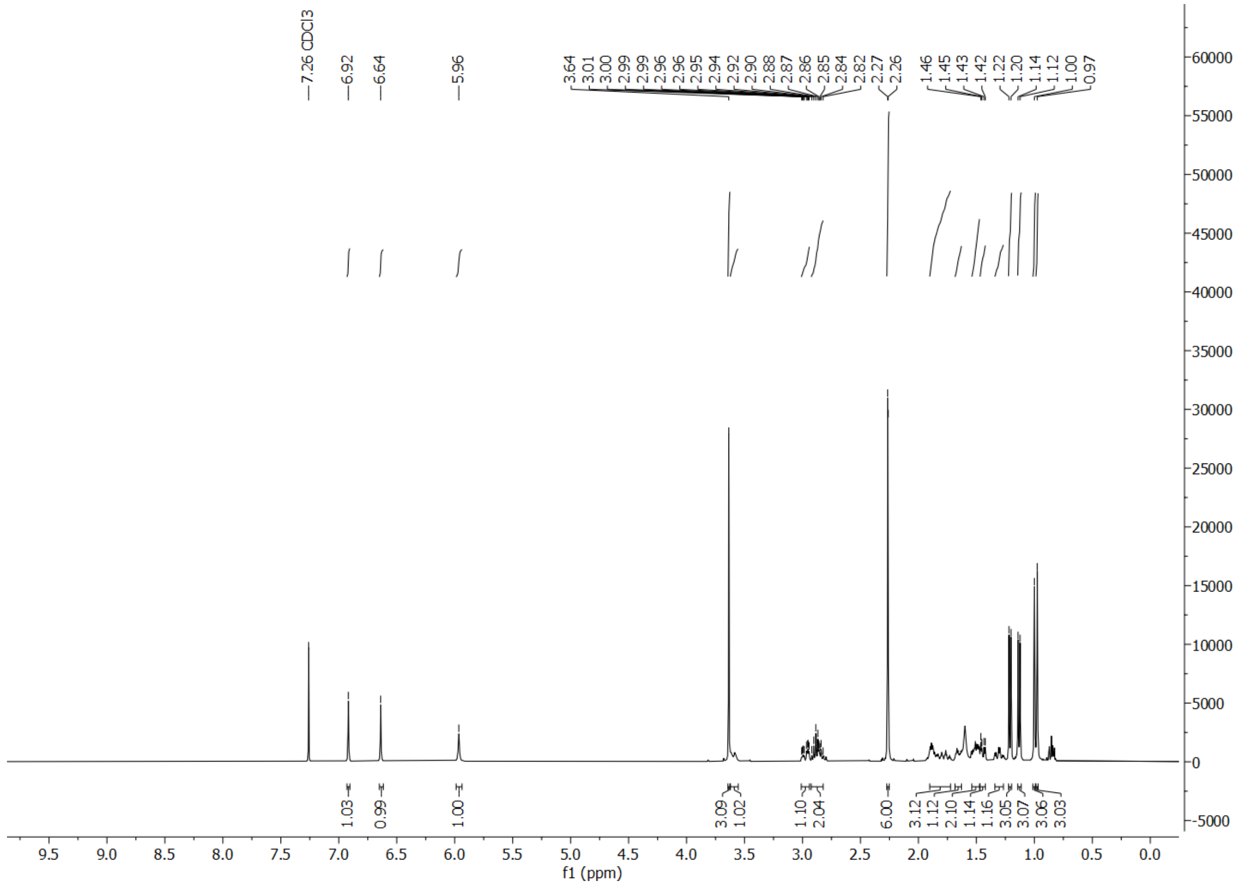

*9.2. ^{13}C spectrum for compound **12** recorded in $CDCl_3$*

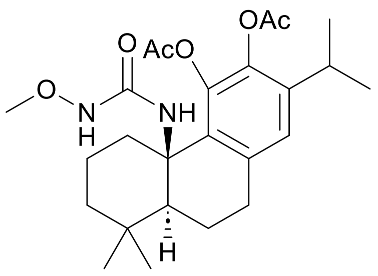

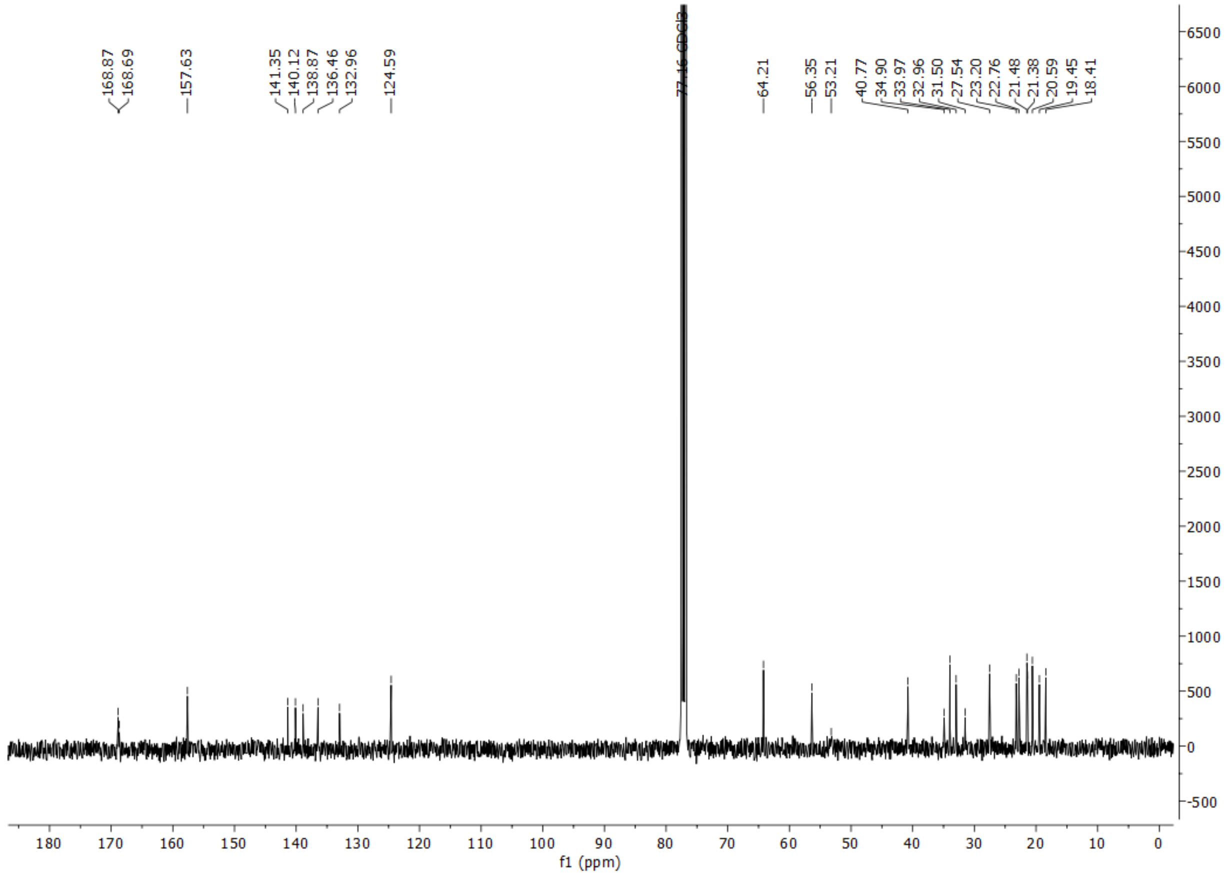

*9.3. COSY spectrum for compound **12** recorded in $CDCl_3$*

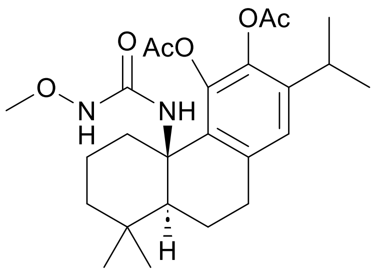

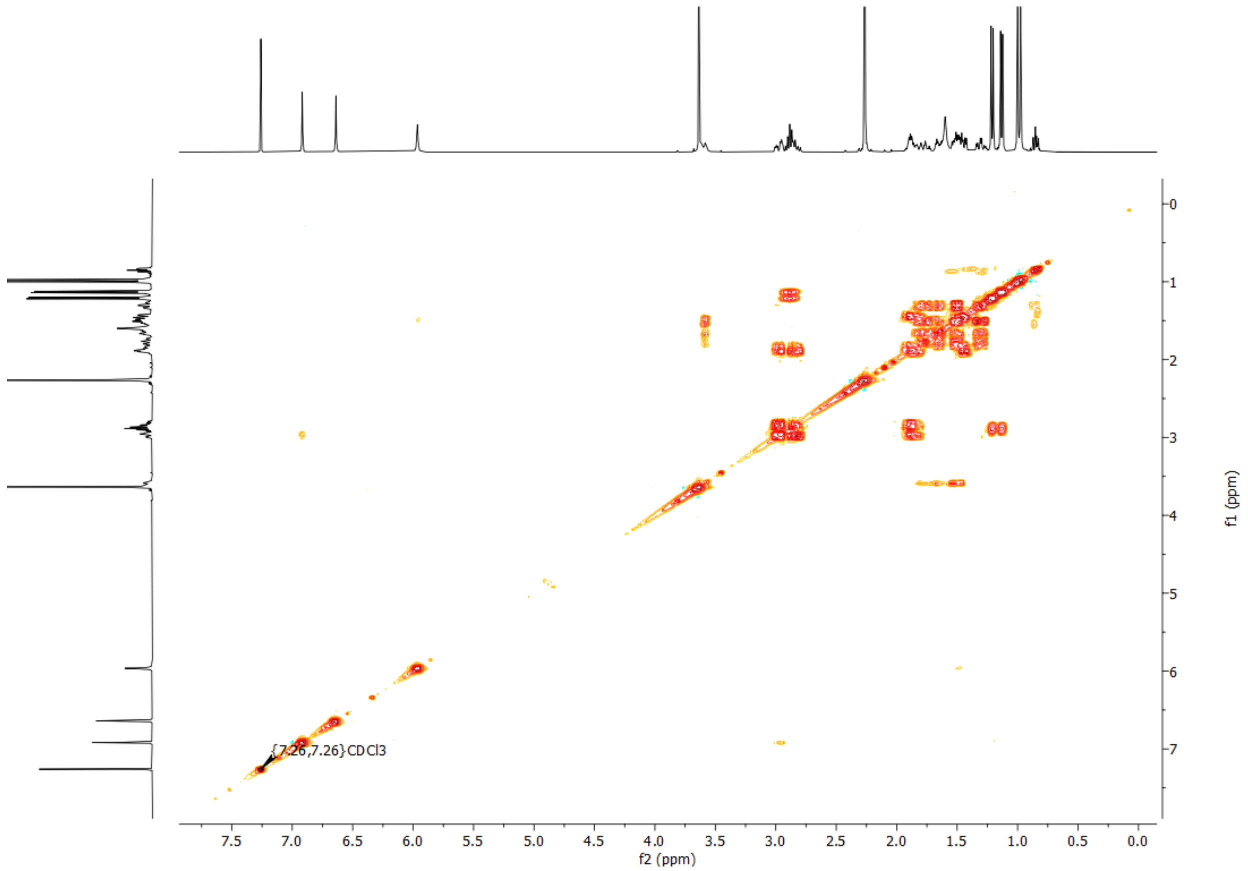

*9.4. NOESY spectrum for compound **12** recorded in $CDCl_3$*

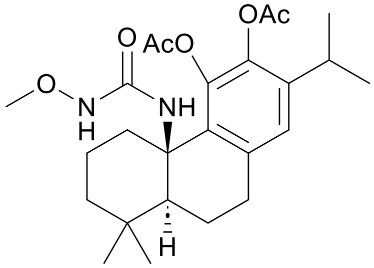

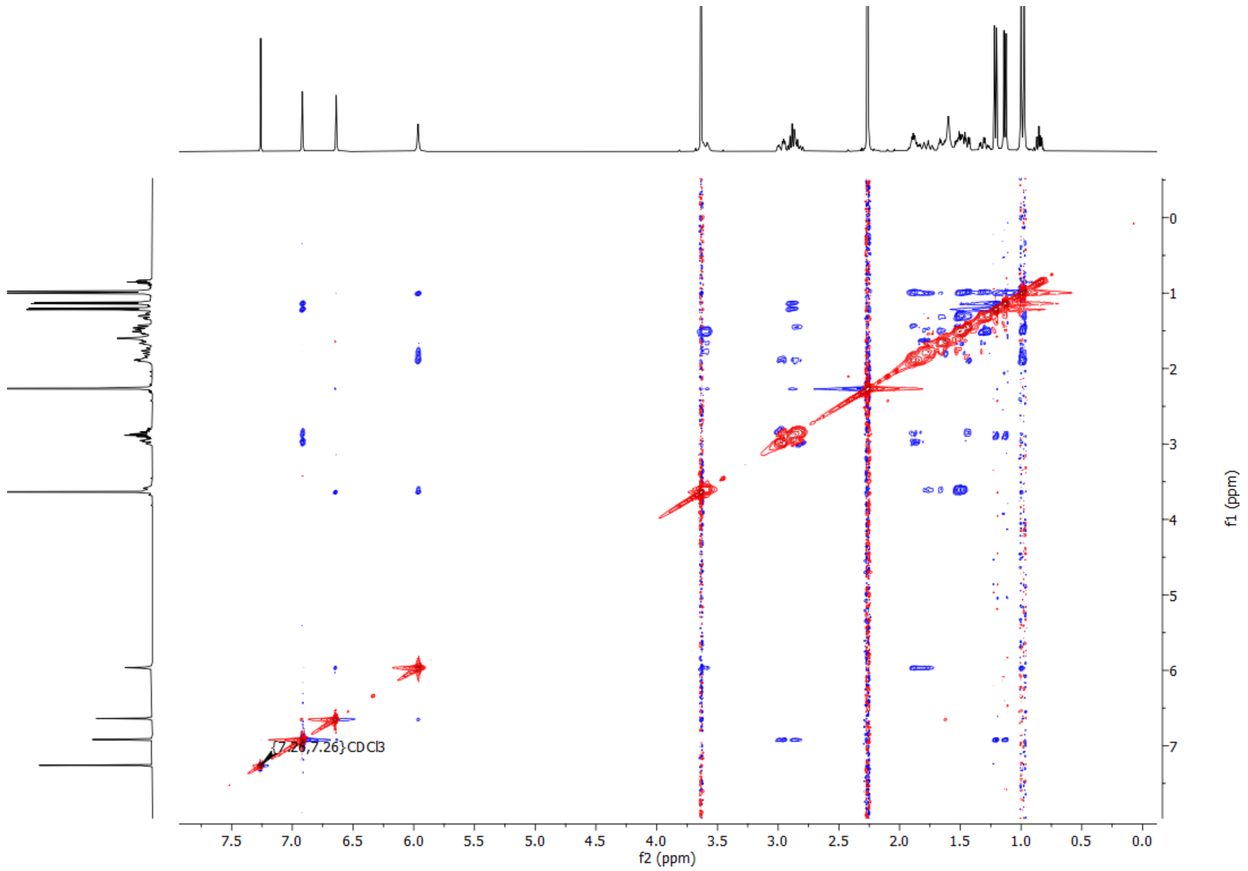

*9.5. HSQC spectrum for compound **12** recorded in $CDCl_3$*

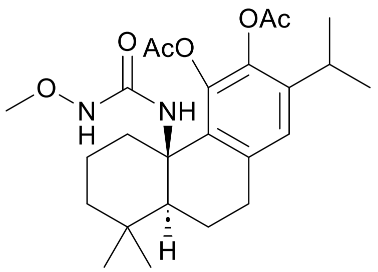

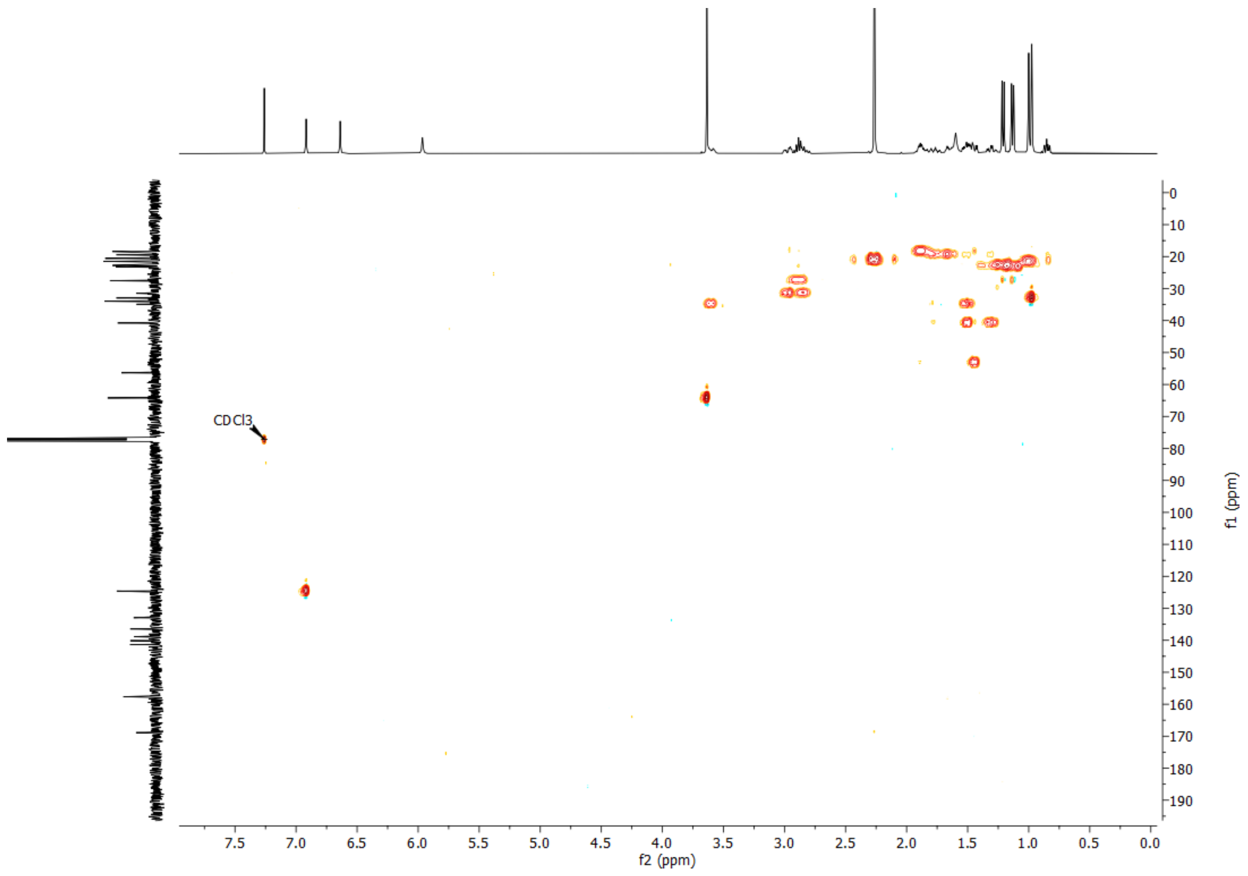

*9.6. HMBC spectrum for compound **12** recorded in $CDCl_3$*

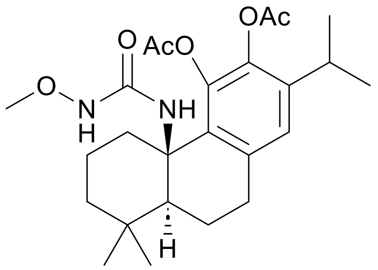

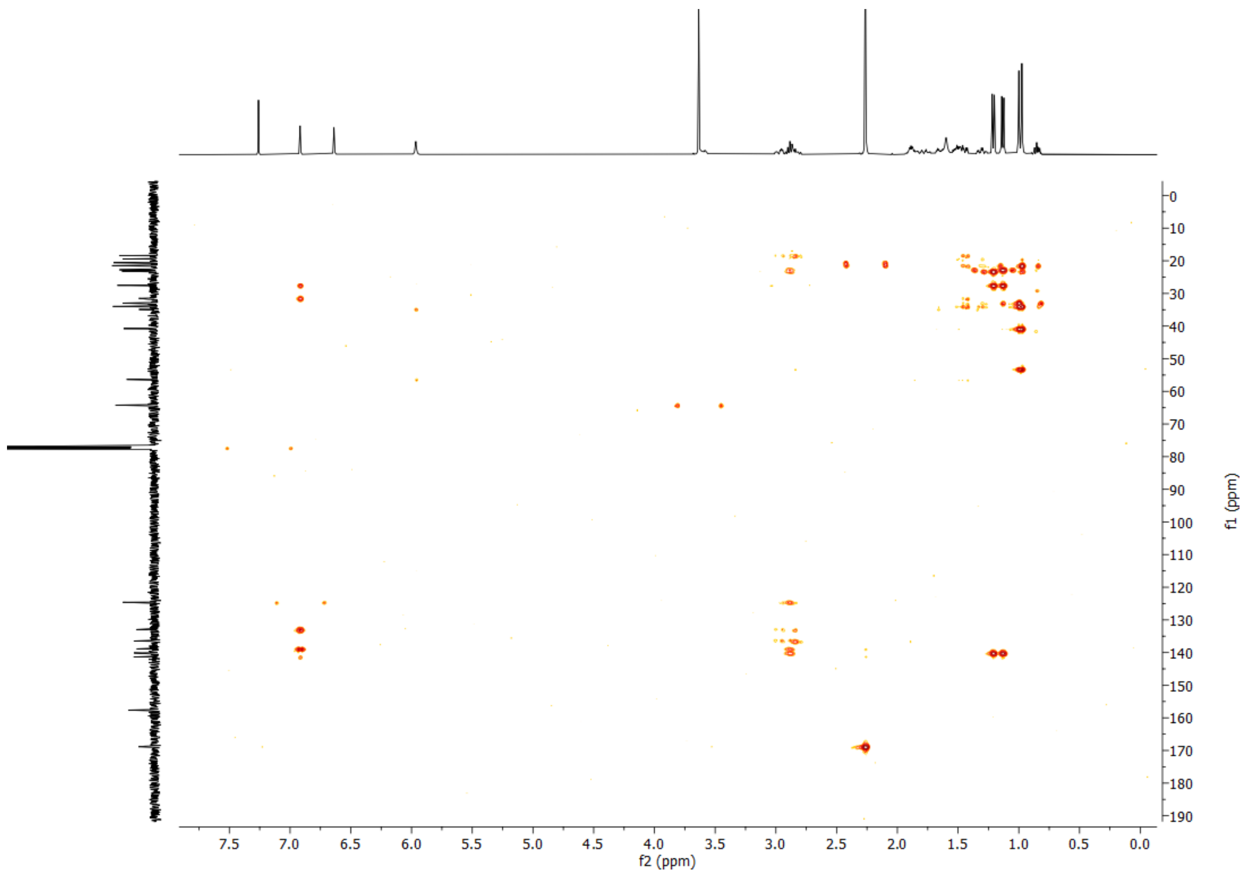

10. NMR data for compound 13

*10.1. ^{1}H spectrum for compound **13** recorded in $CDCl_3$*

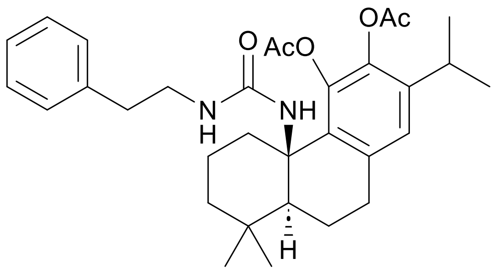

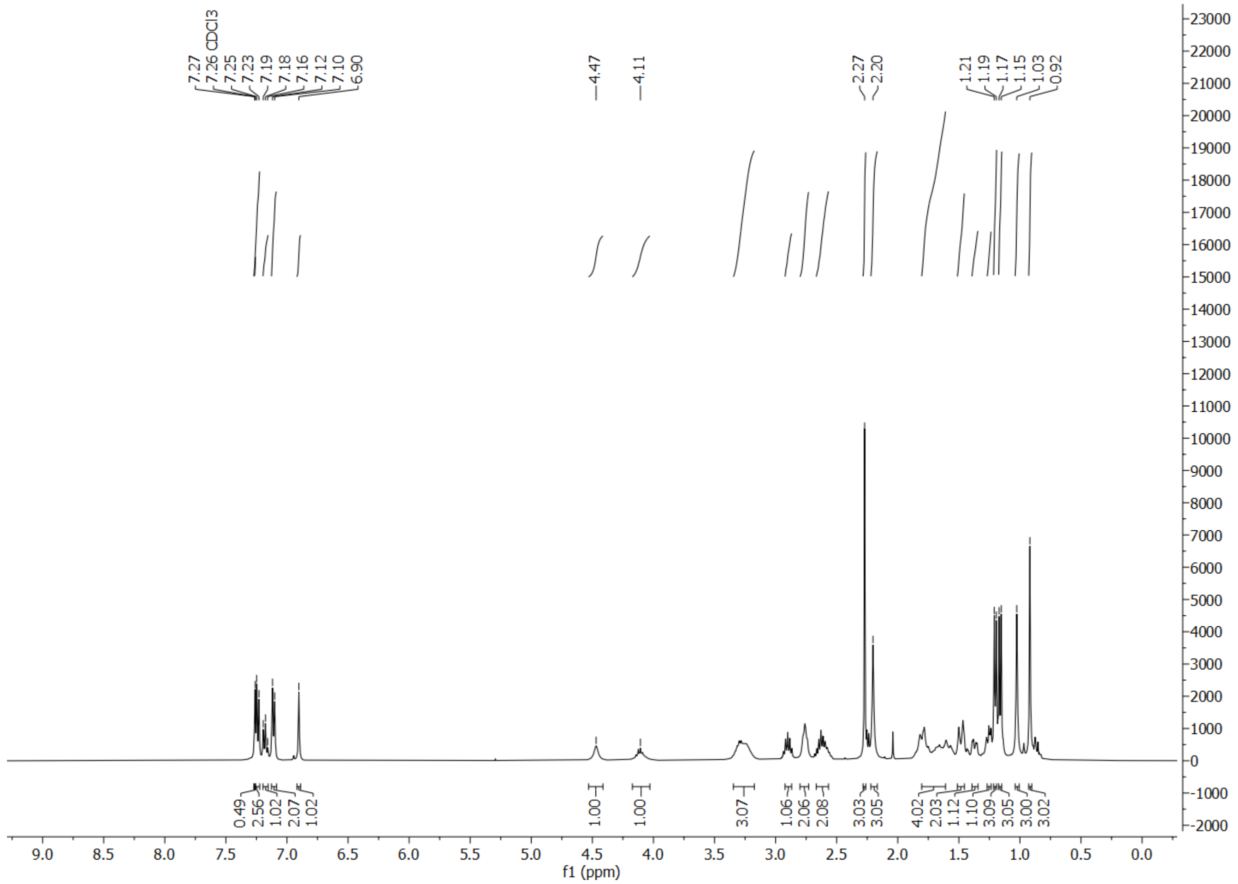

*10.2. ^{13}C spectrum for compound **13** recorded in $CDCl_3$*

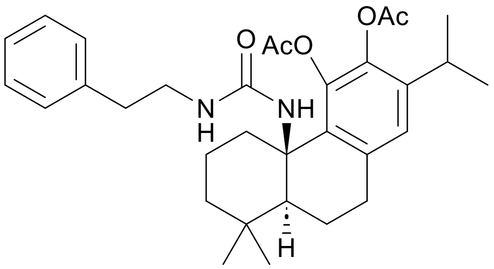

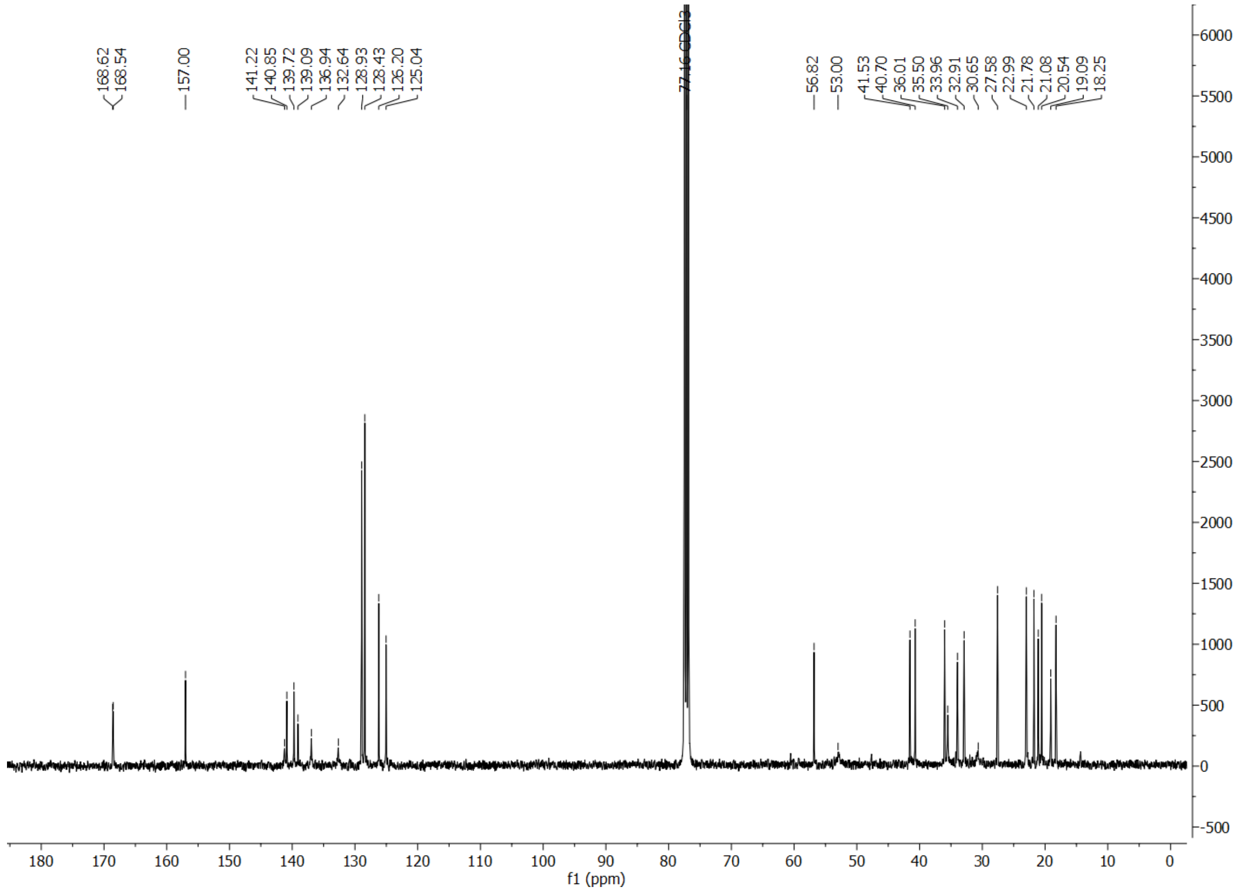

11. NMR data for compound 14

*11.1. ^{1}H spectrum for compound **14** recorded in $CDCl_3$*

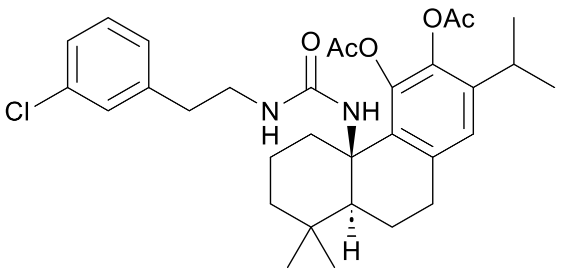

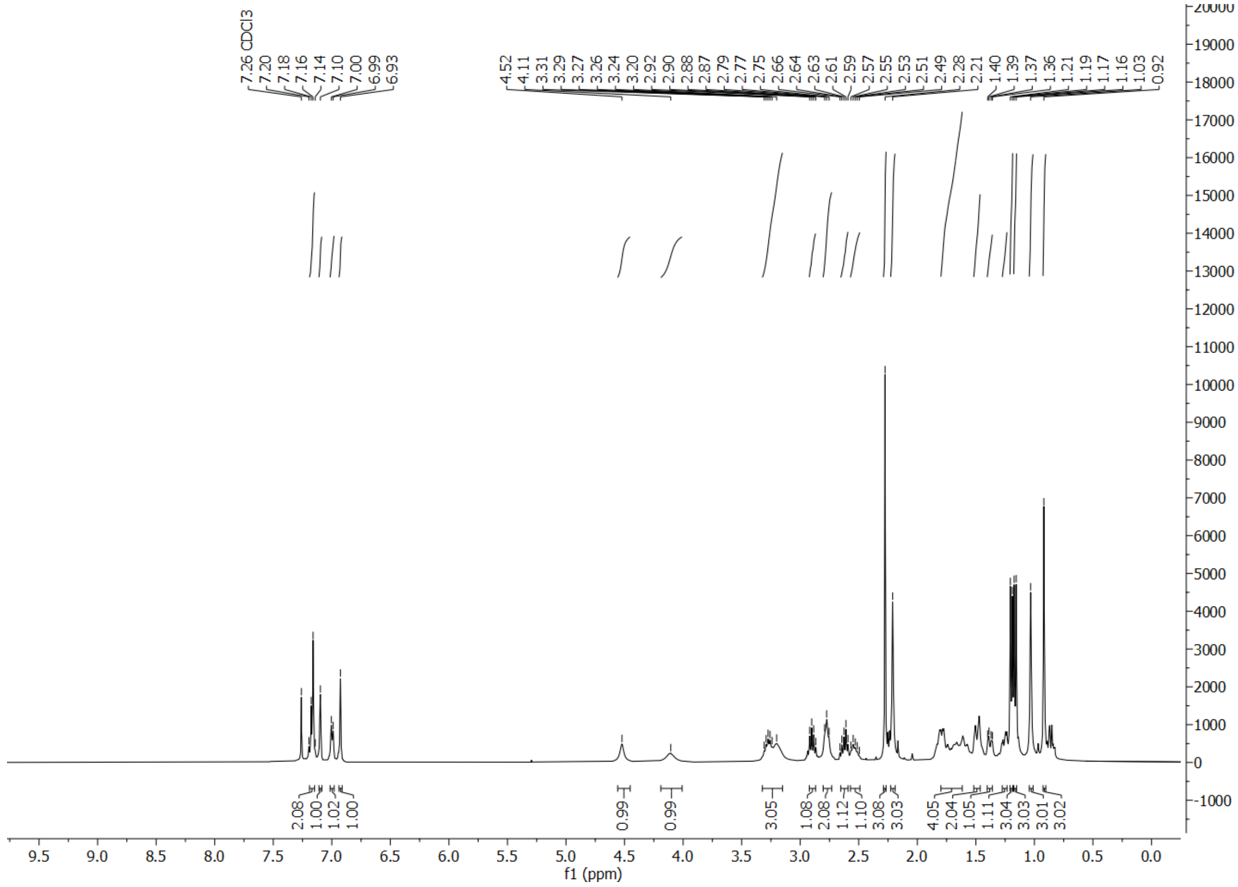

*11.2. ^{13}C spectrum for compound **14** recorded in $CDCl_3$*

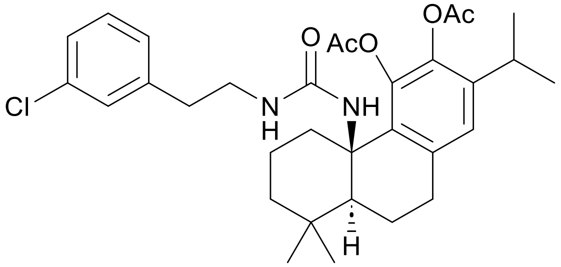

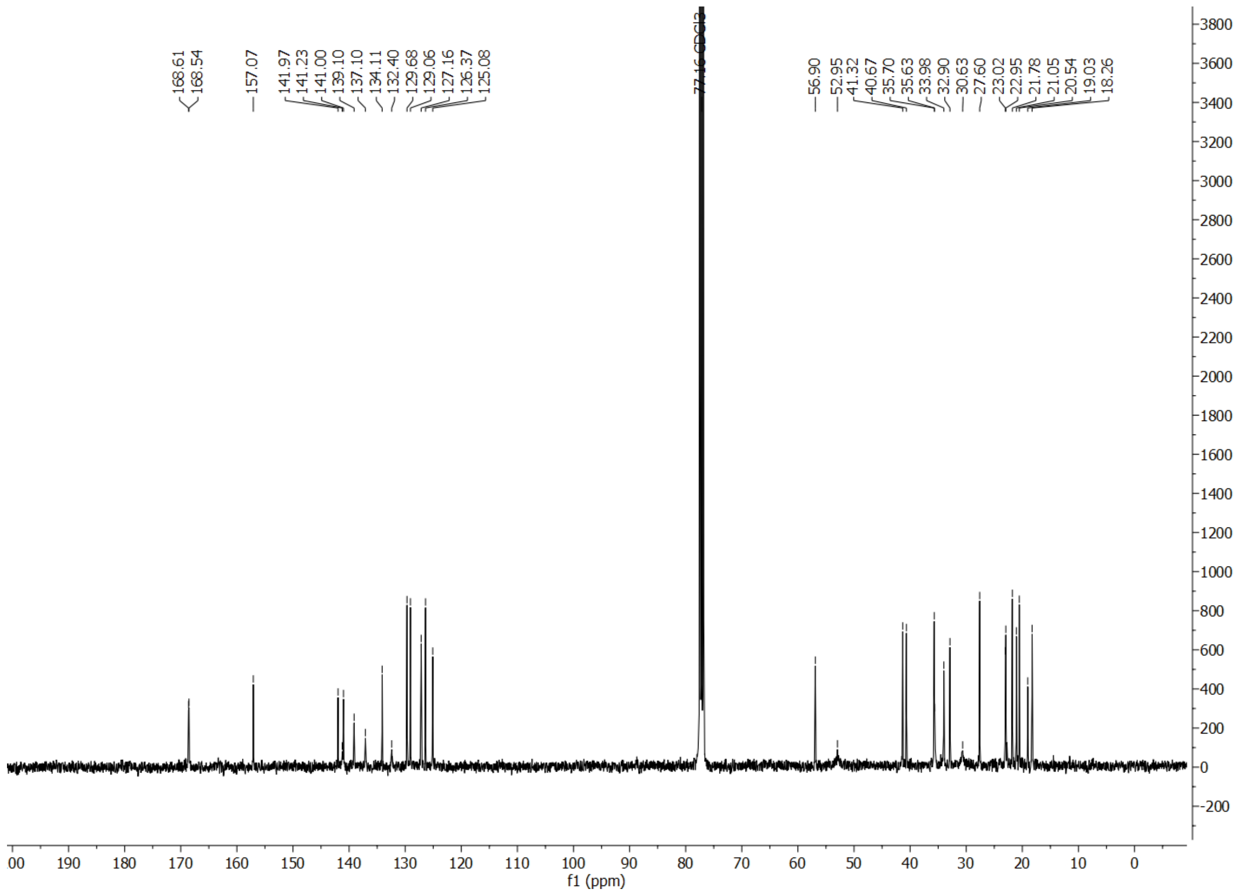

*11.3. COSY spectrum for compound **14** recorded in $CDCl_3$*

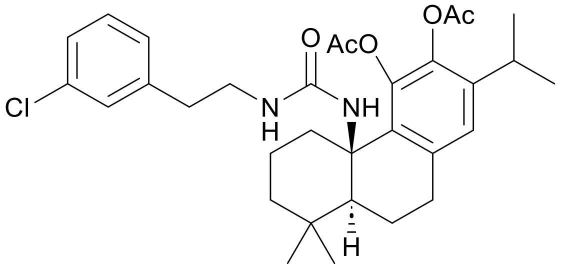

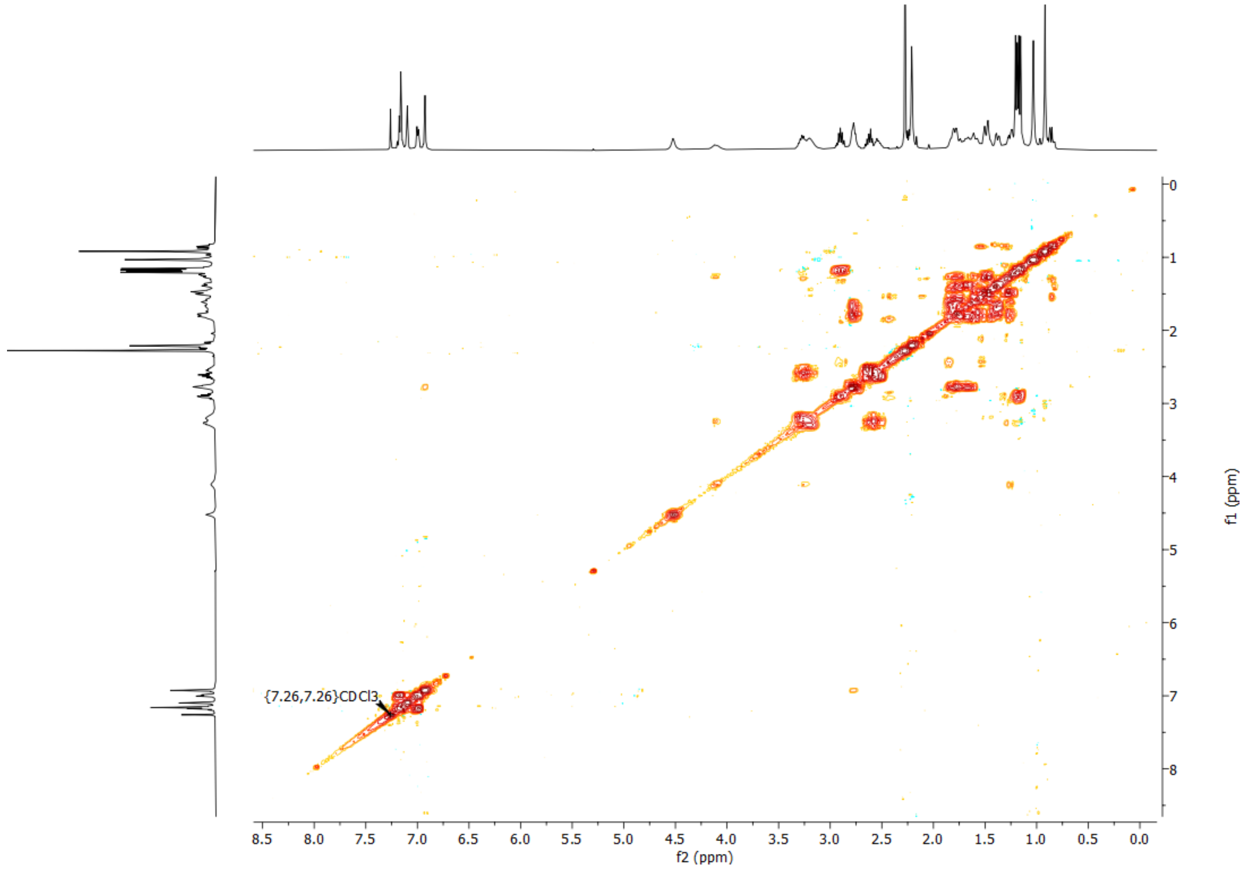

*11.4. NOESY spectrum for compound **14** recorded in $CDCl_3$*

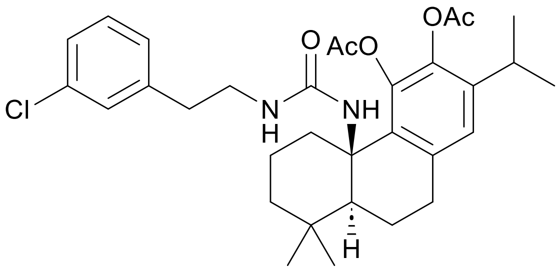

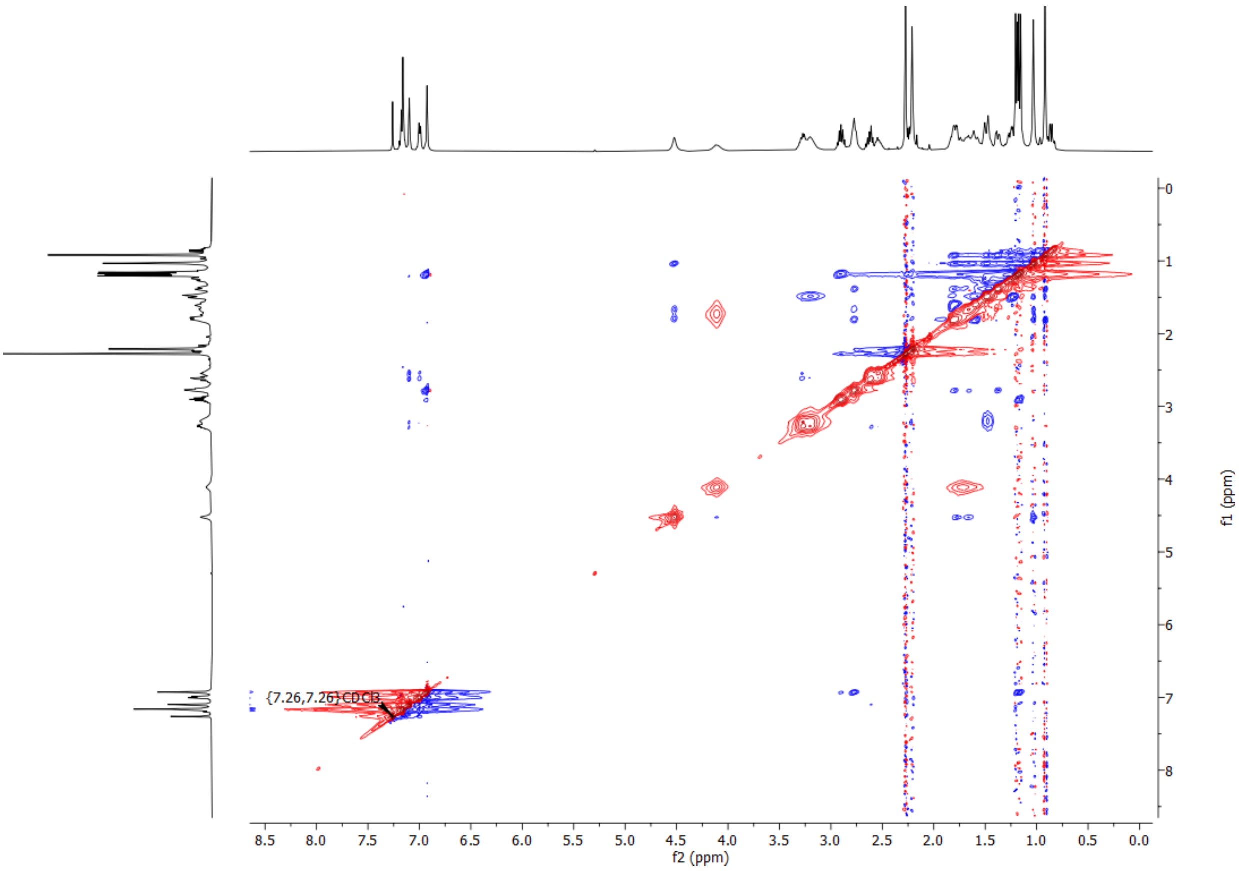

*11.5. HSQC spectrum for compound **14** recorded in $CDCl_3$*

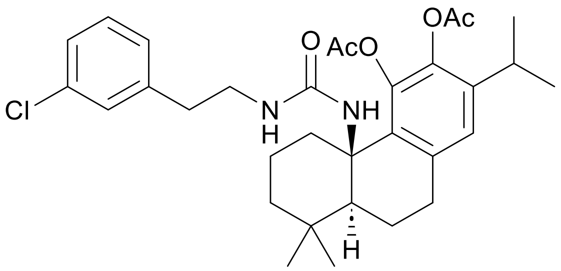

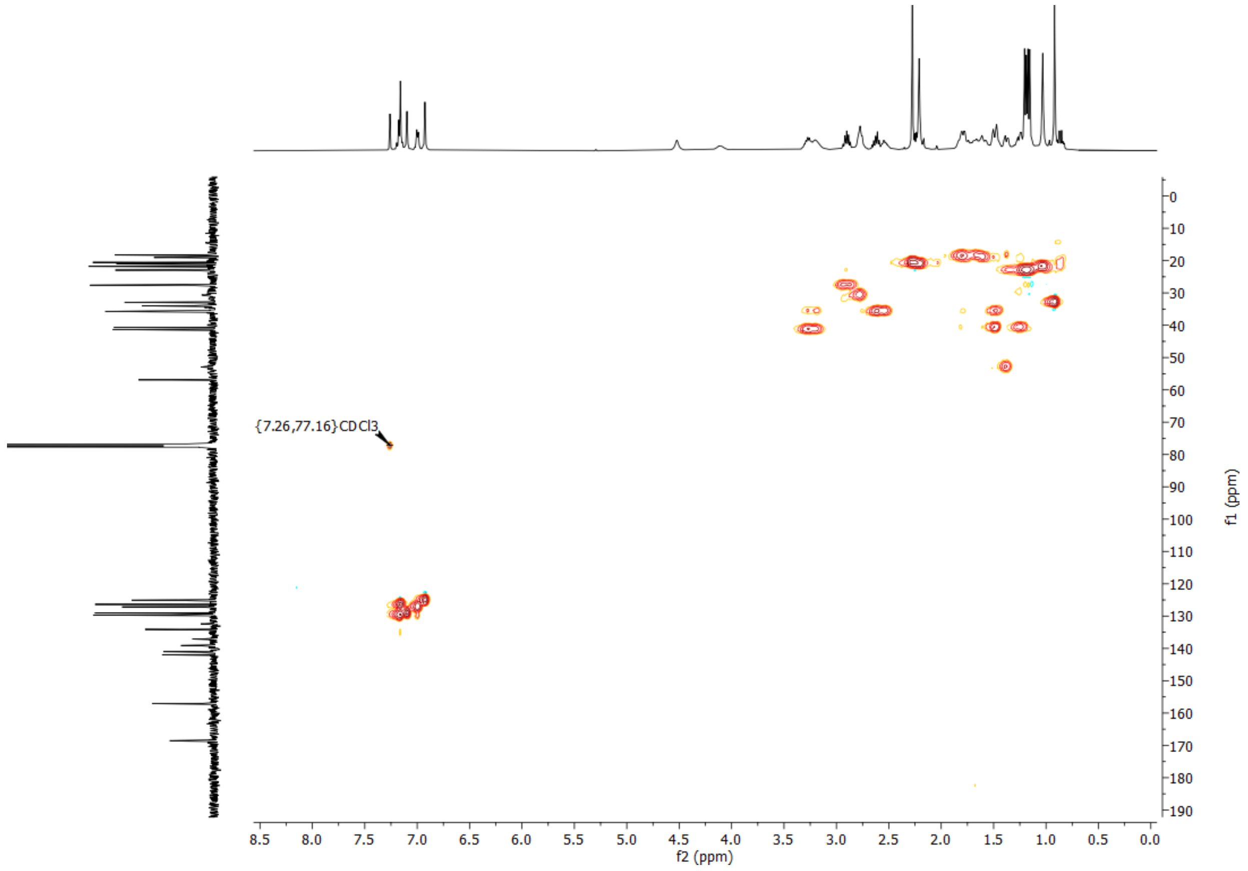

*11.6. HMBC spectrum for compound **14** recorded in $CDCl_3$*

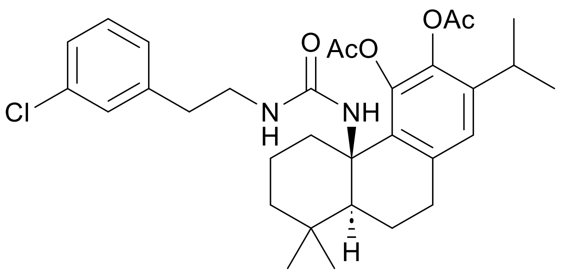

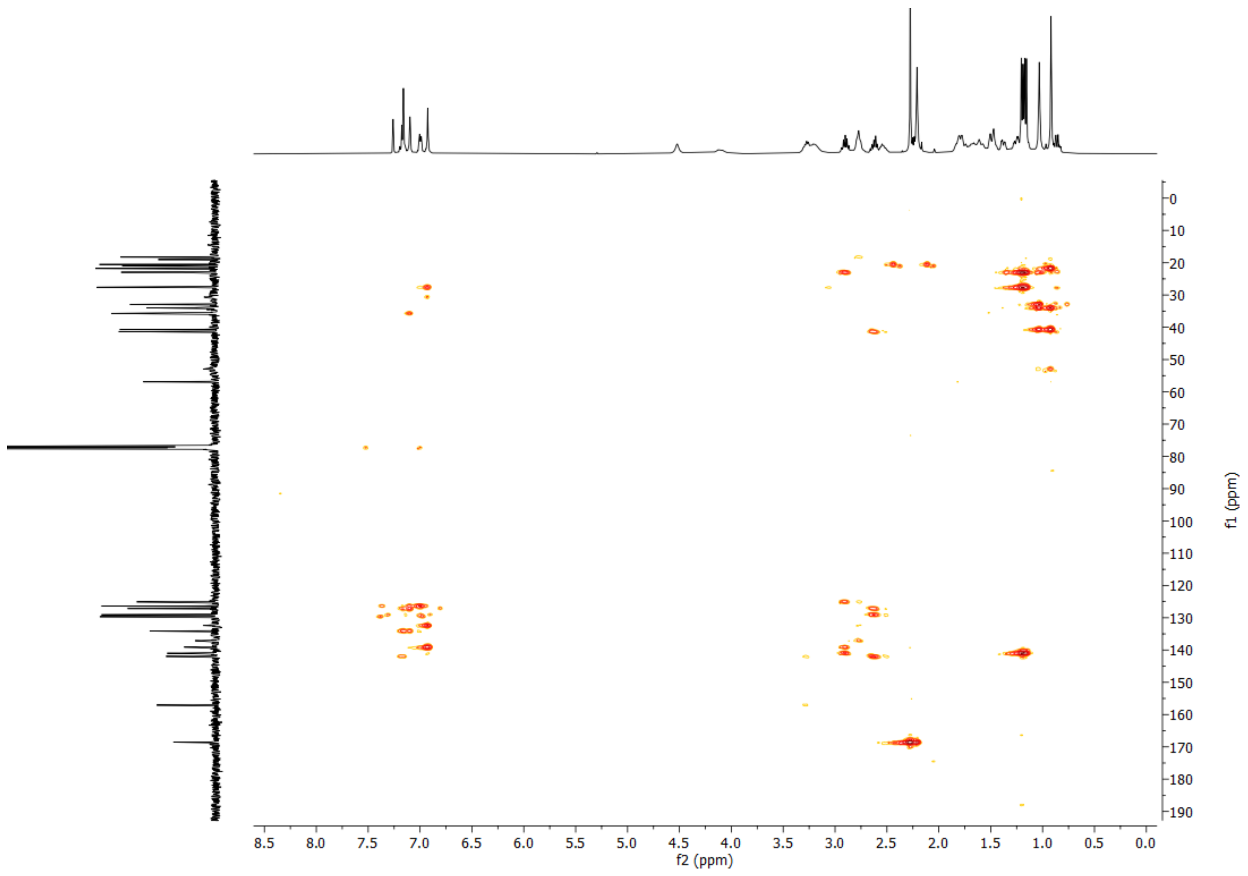

12. NMR data for compound 15

*12.1. ^{1}H spectrum for compound **15** recorded in $CDCl_3$*

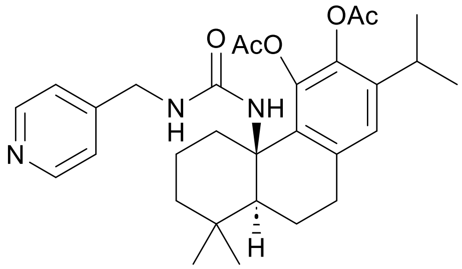

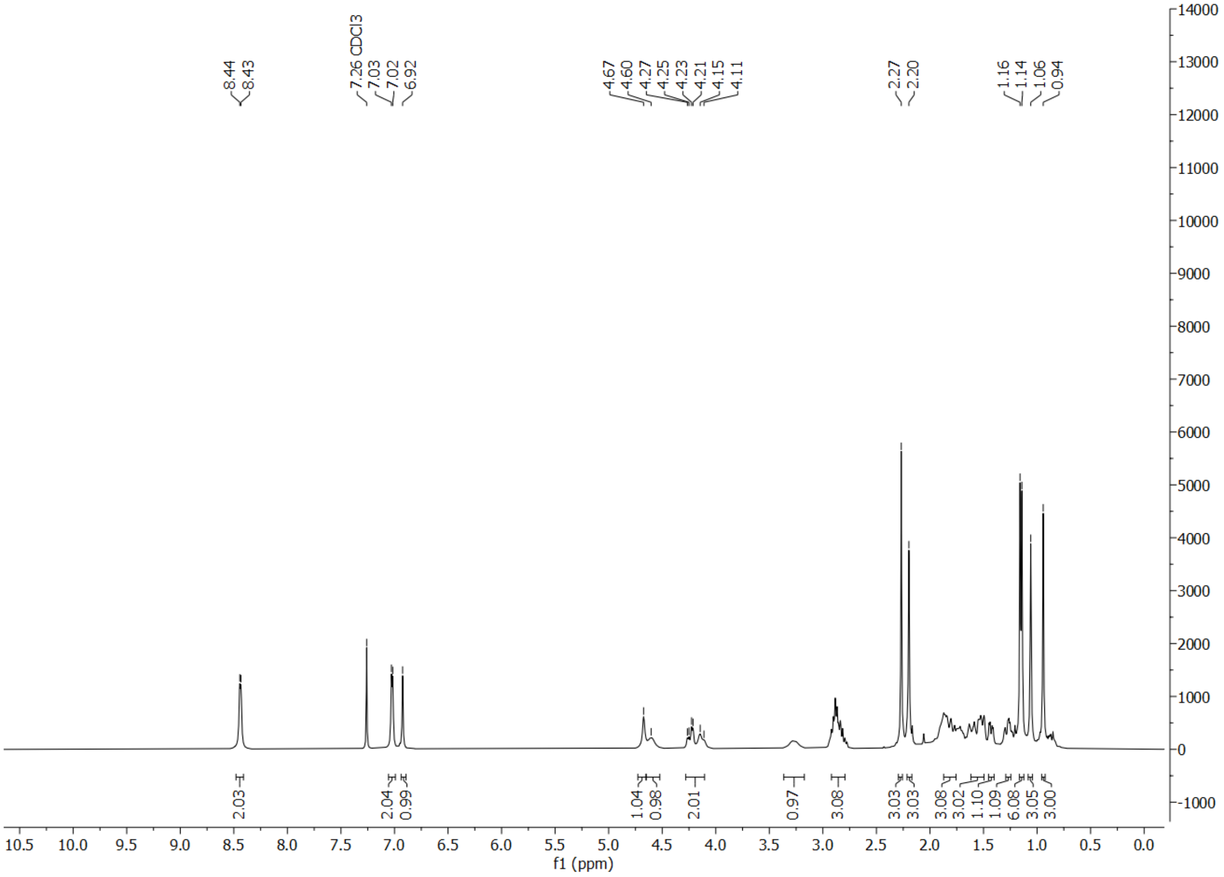

*12.2. ^{13}C spectrum for compound **15** recorded in $CDCl_3$*

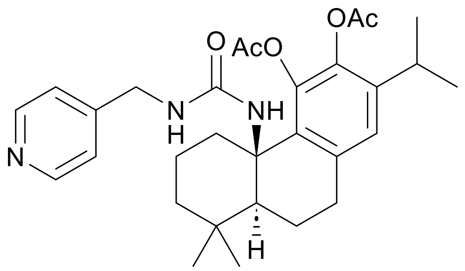

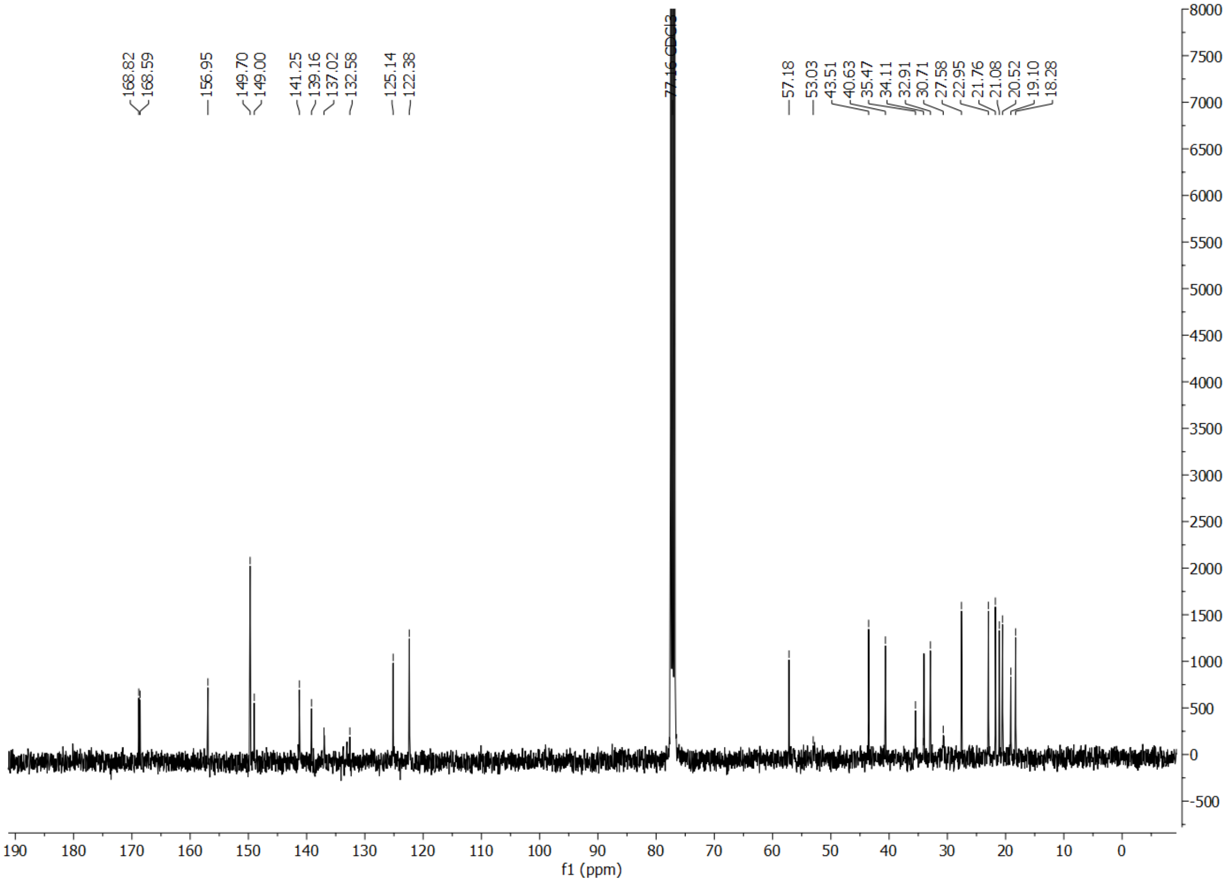

13. NMR data for compound 16

*13.1. ¹H spectrum for compound **16** recorded in CDCl₃*

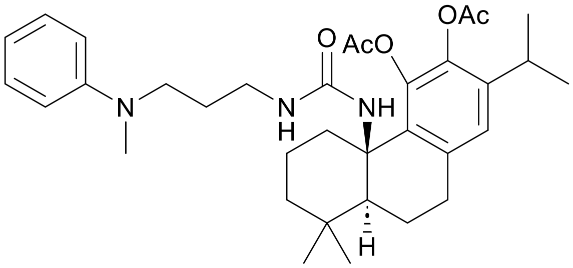

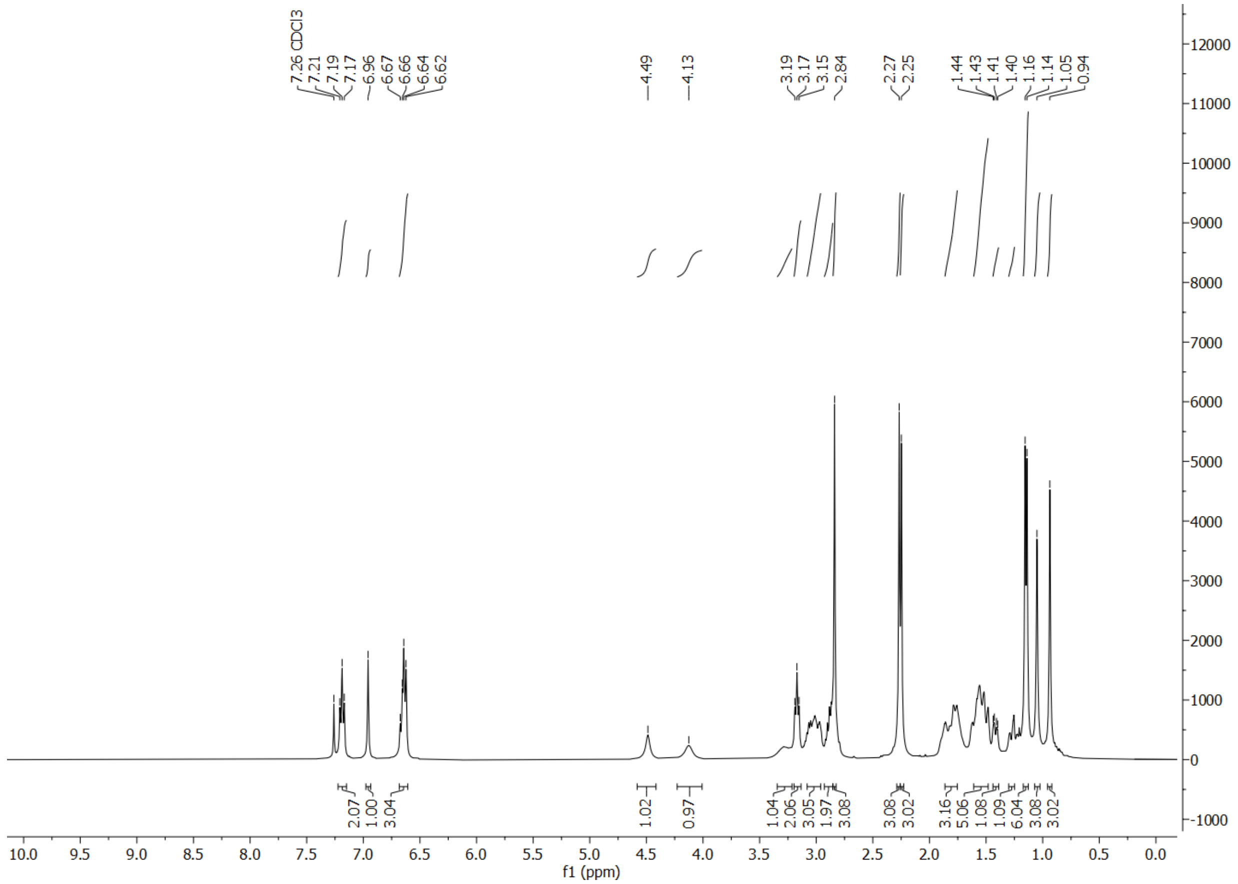

*13.2. ^{13}C spectrum for compound **16** recorded in $CDCl_3$*

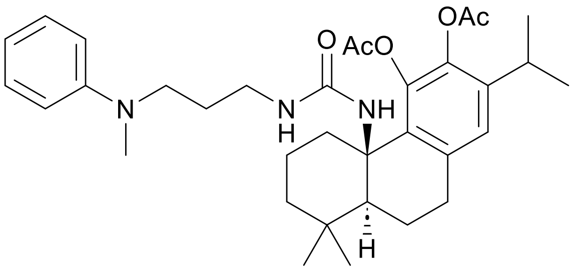

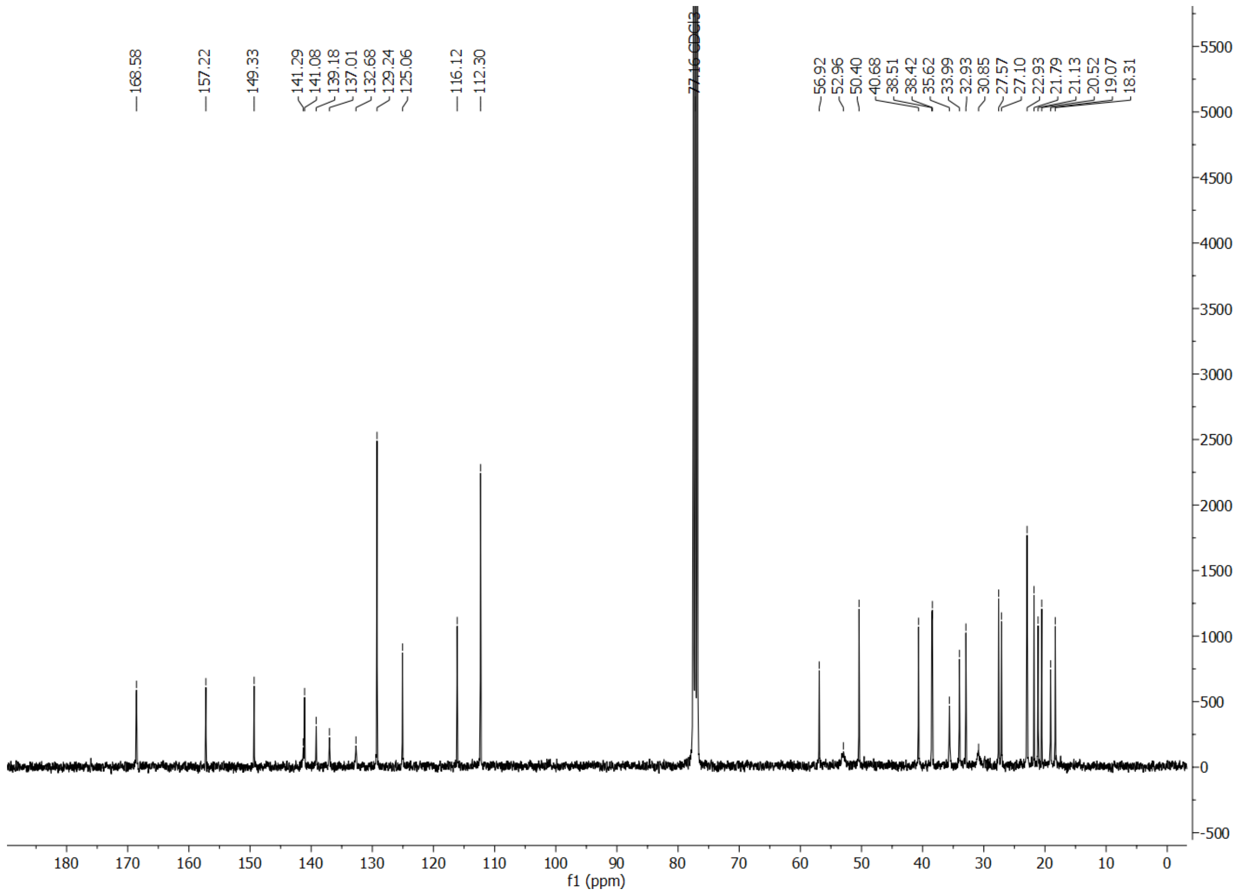

*13.3. COSY spectrum for compound **16** recorded in $CDCl_3$*

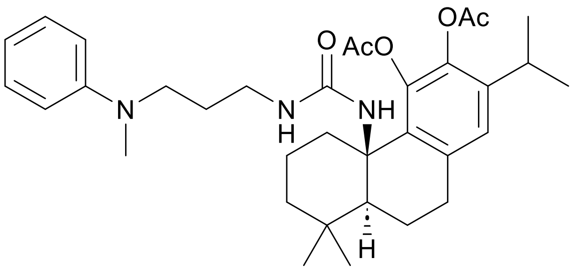

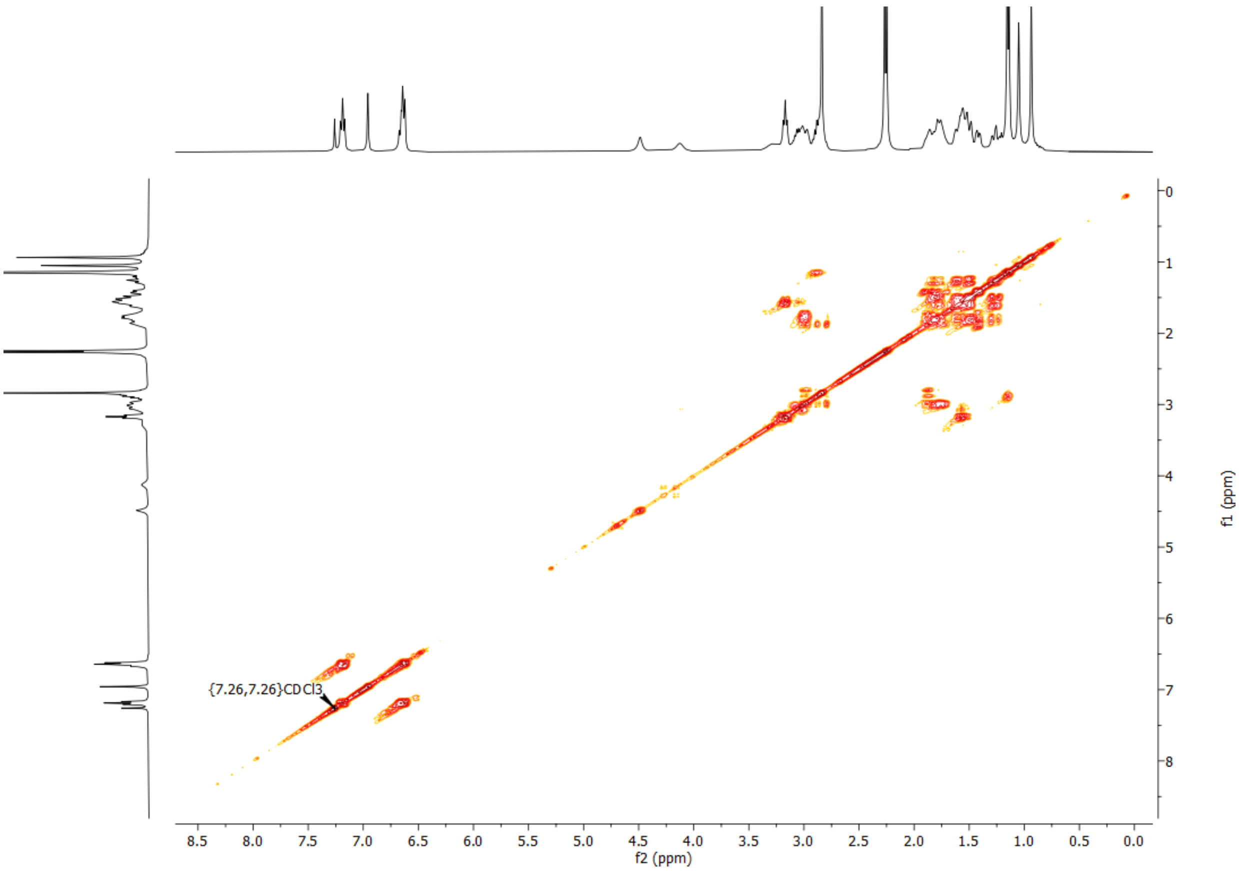

*13.4. HSQC spectrum for compound **16** recorded in $CDCl_3$*

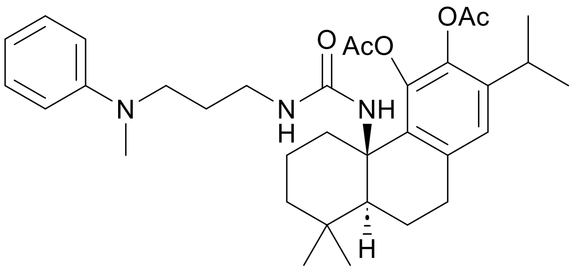

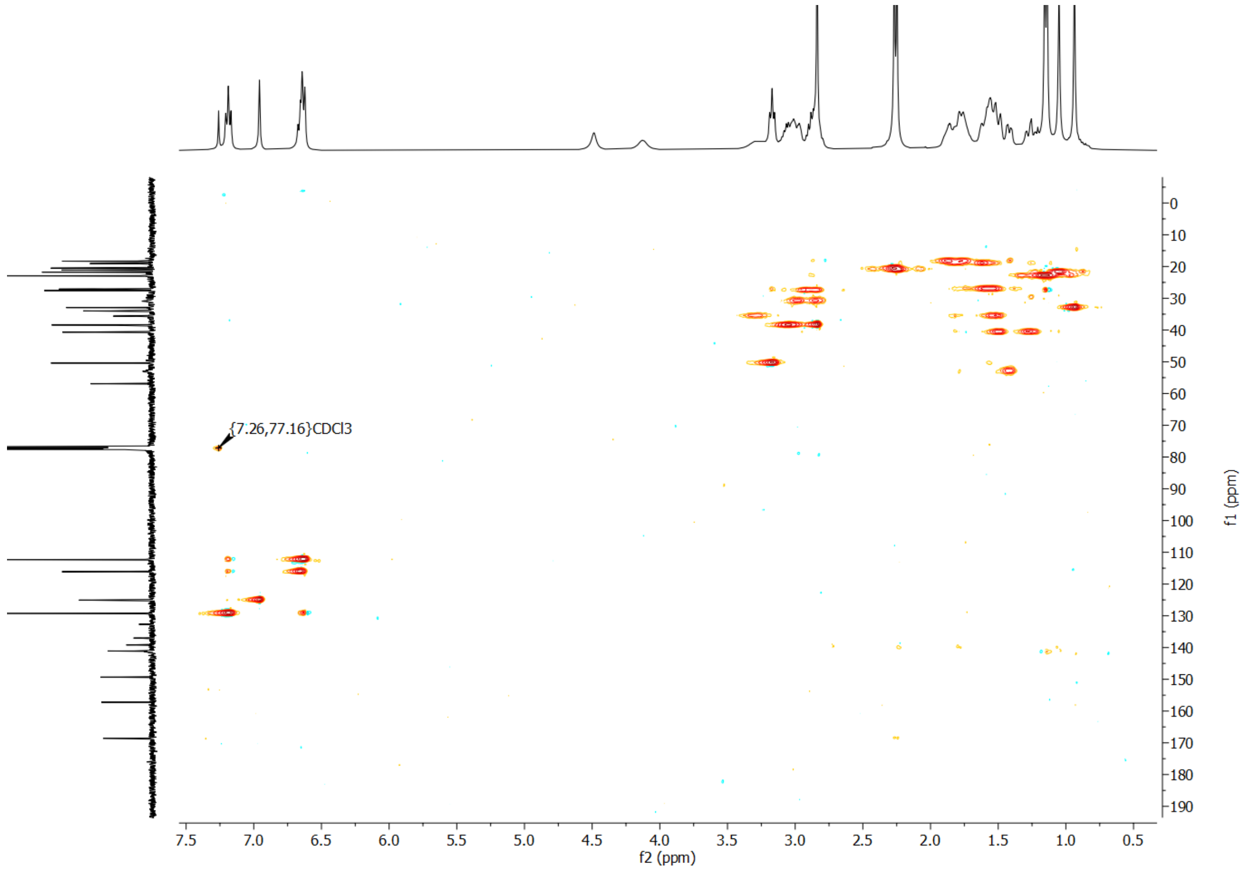

*13.5. HMBC spectrum for compound **16** recorded in $CDCl_3$*

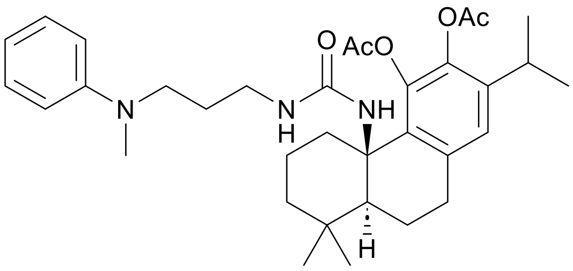

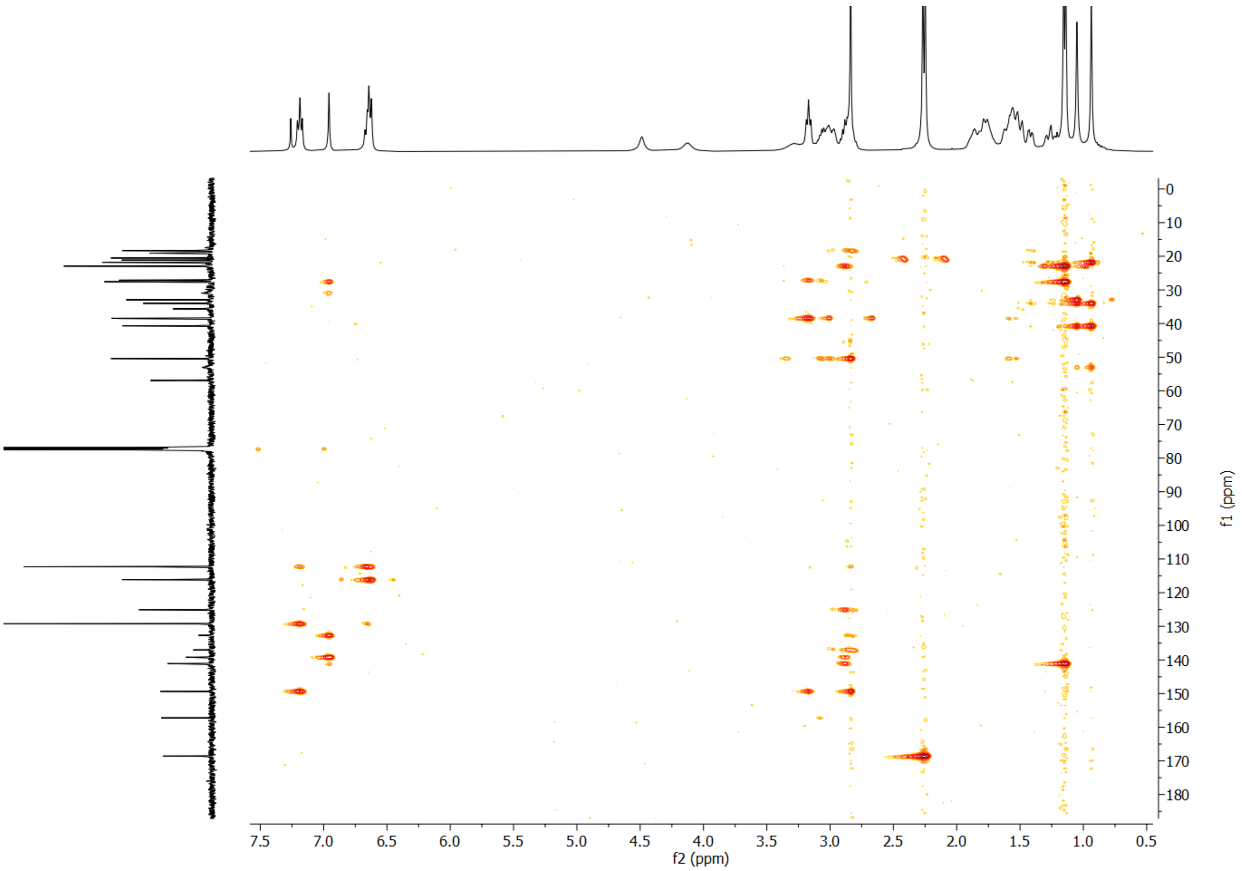

14. NMR data for compound 17

*14.1. ¹H spectrum for compound **17** recorded in $CDCl_3$*

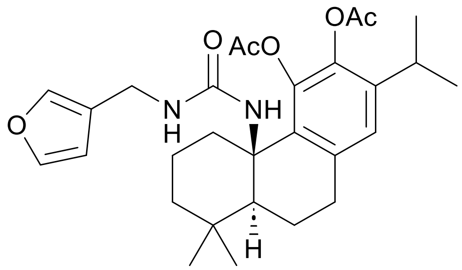

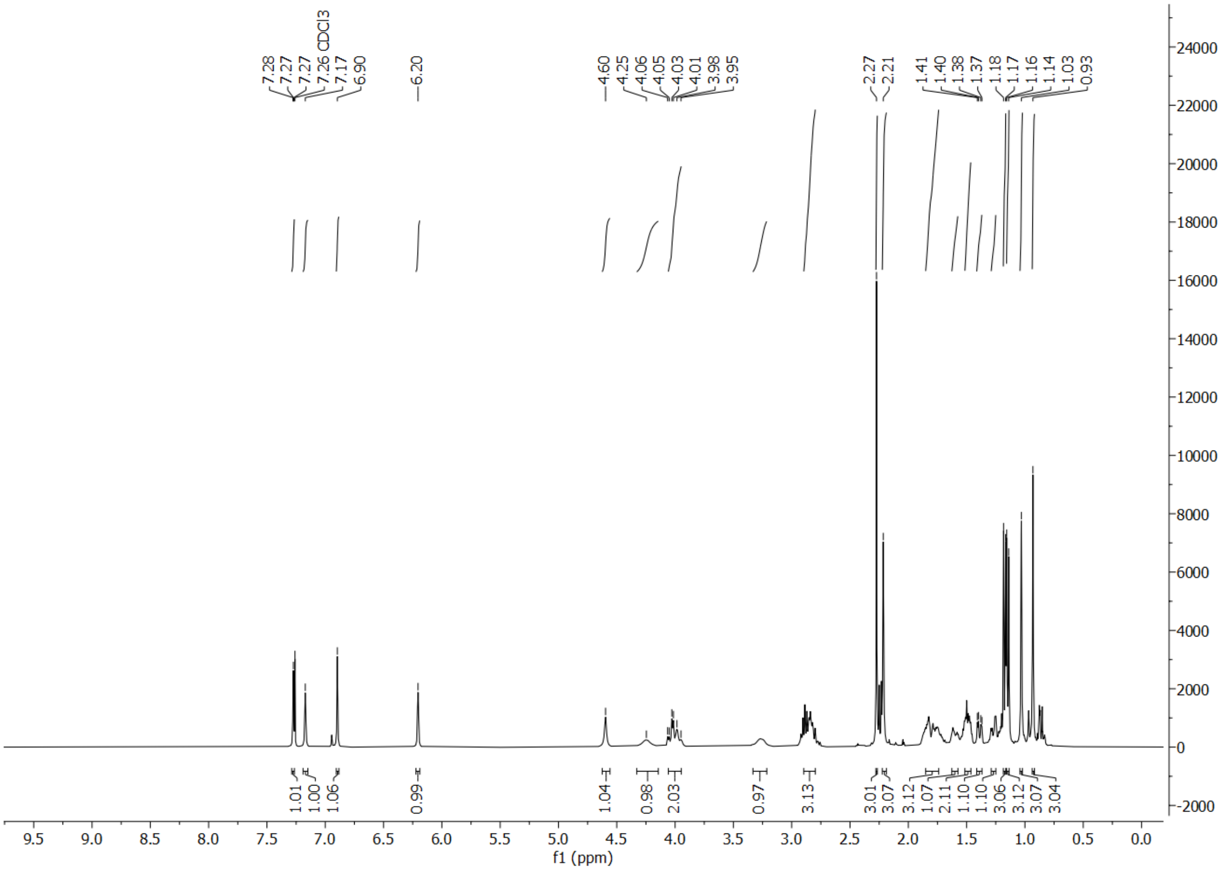

*14.2. ^{13}C spectrum for compound **17** recorded in $CDCl_3$*

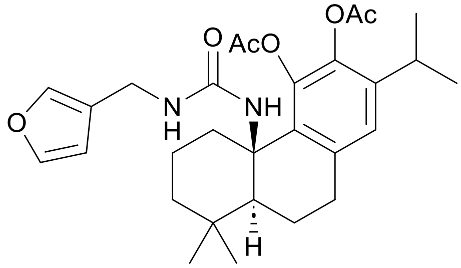

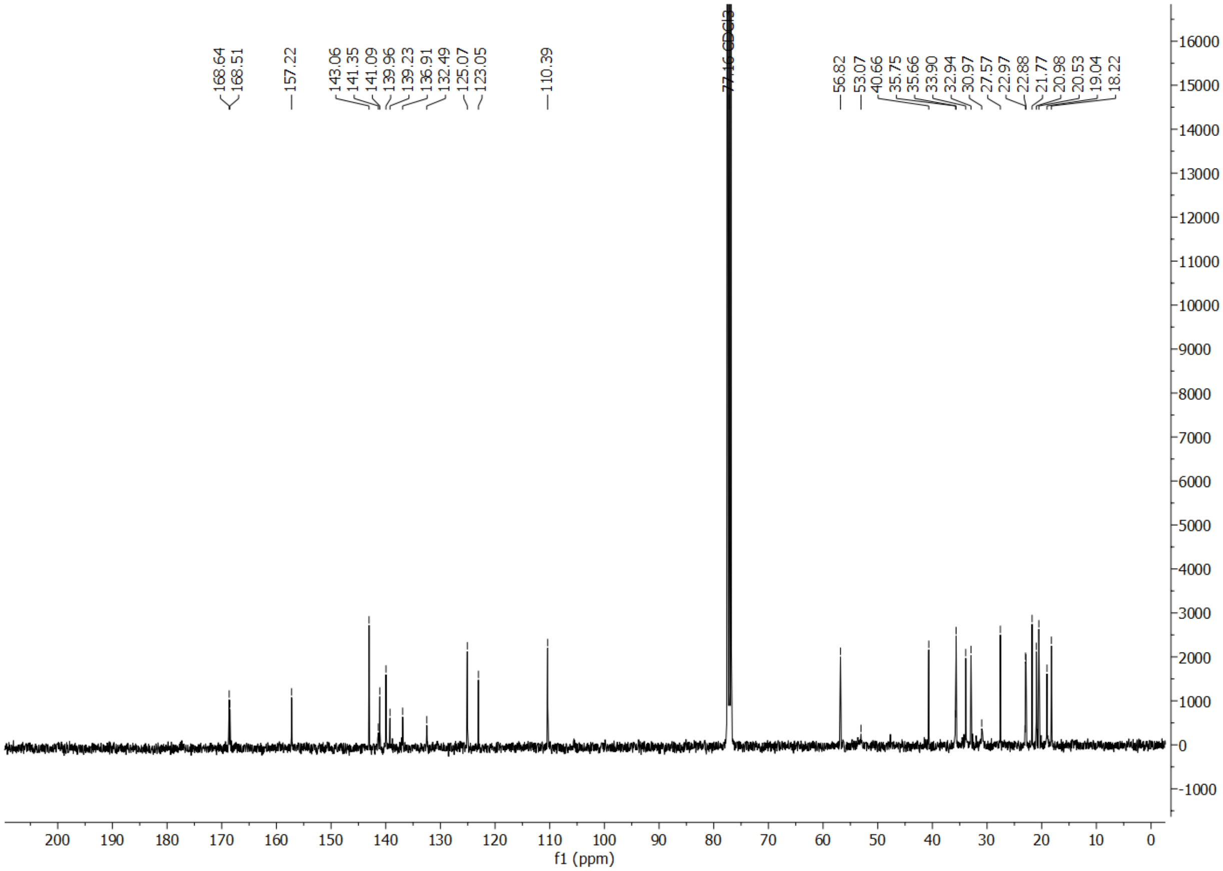

15. NMR data for compound 18

*15.1. ^{1}H spectrum for compound **18** recorded in $CDCl_3$*

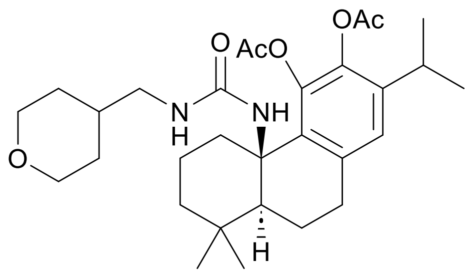

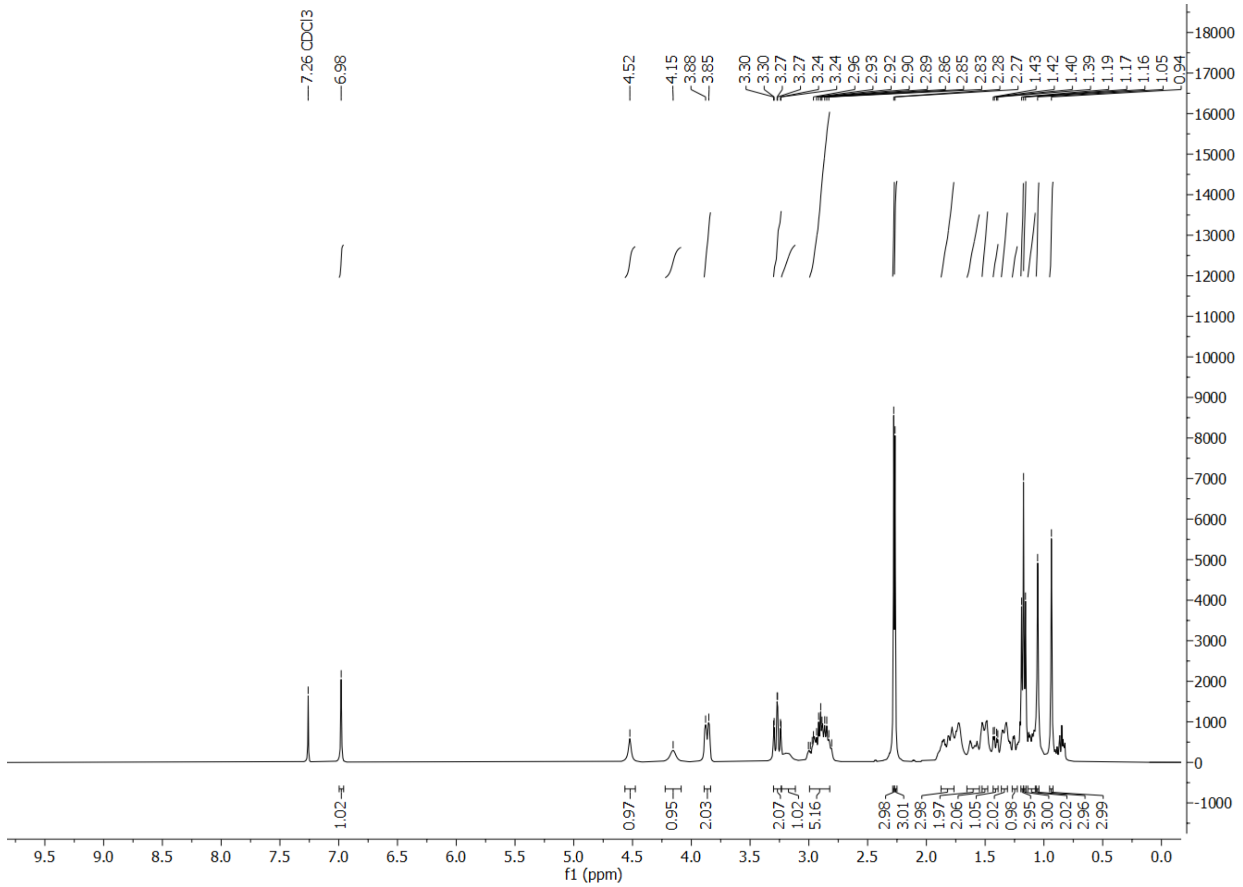

*15.2. ^{13}C spectrum for compound **18** recorded in $CDCl_3$*

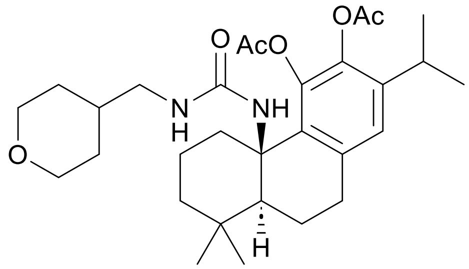

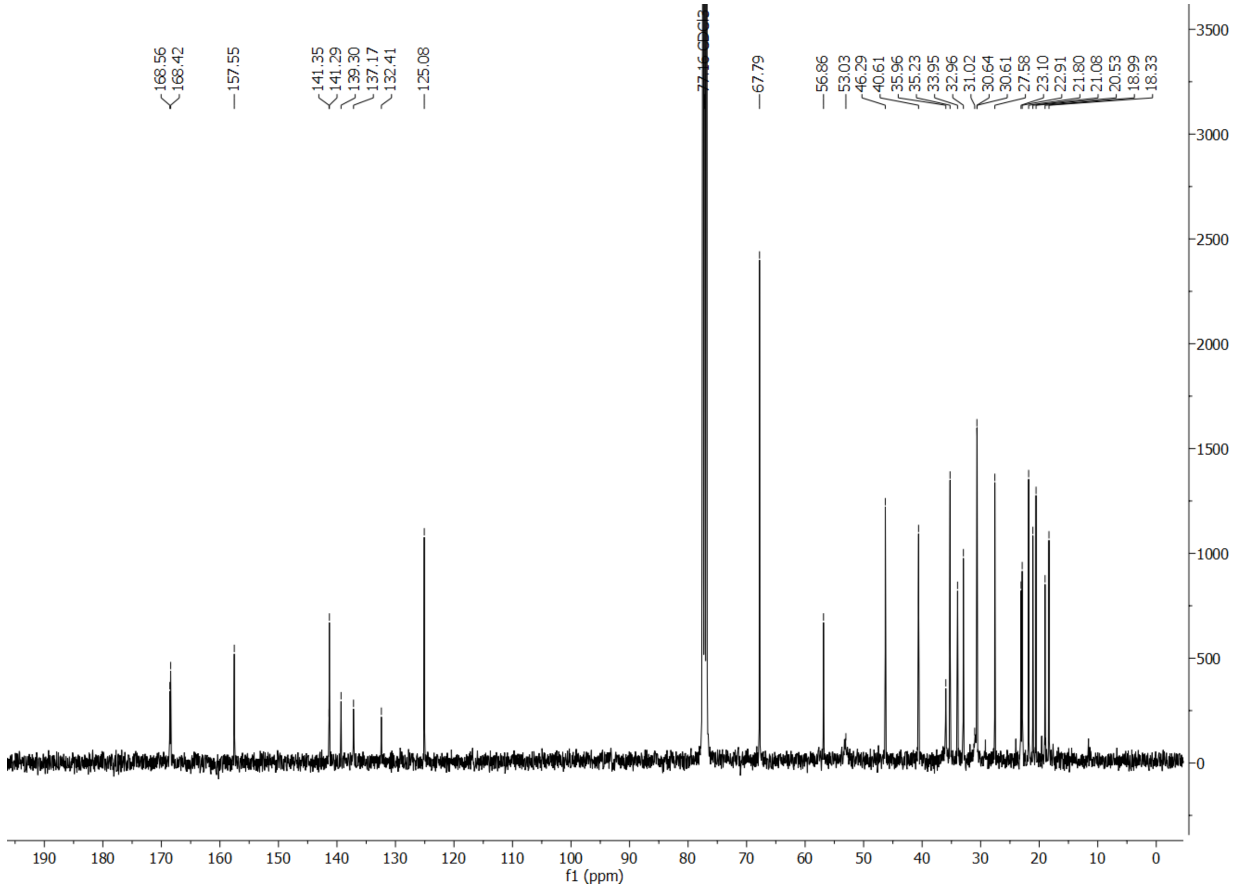

*15.3. HSQC spectrum for compound **18** recorded in $CDCl_3$*

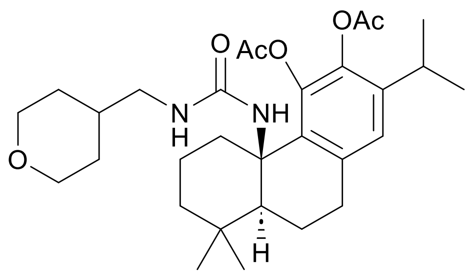

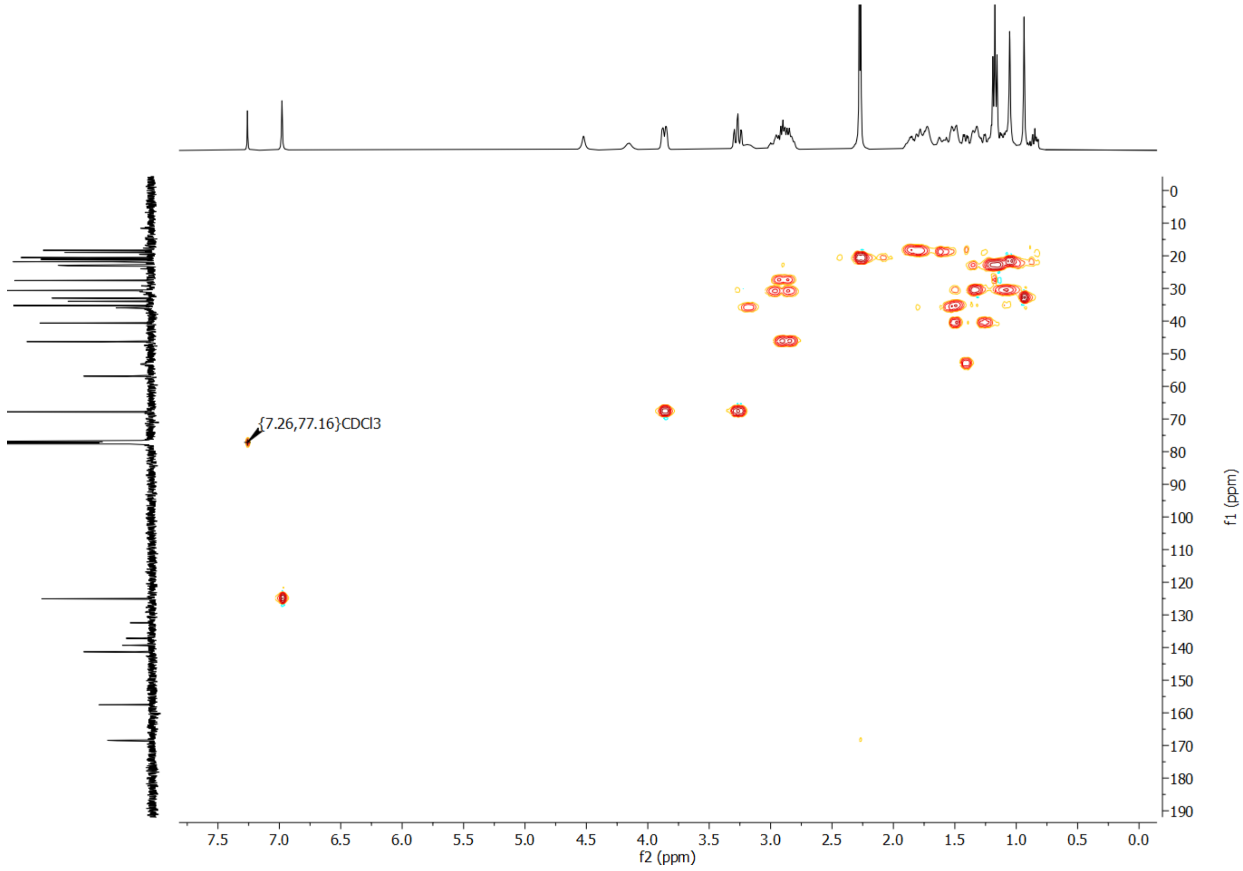

16. NMR data for compound 19

*16.1. ^{1}H spectrum for compound **19** recorded in $CDCl_3$*

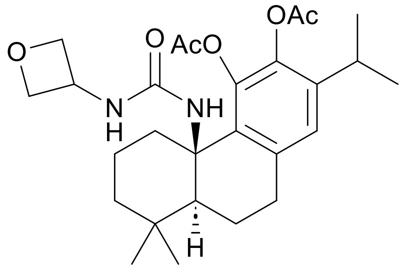

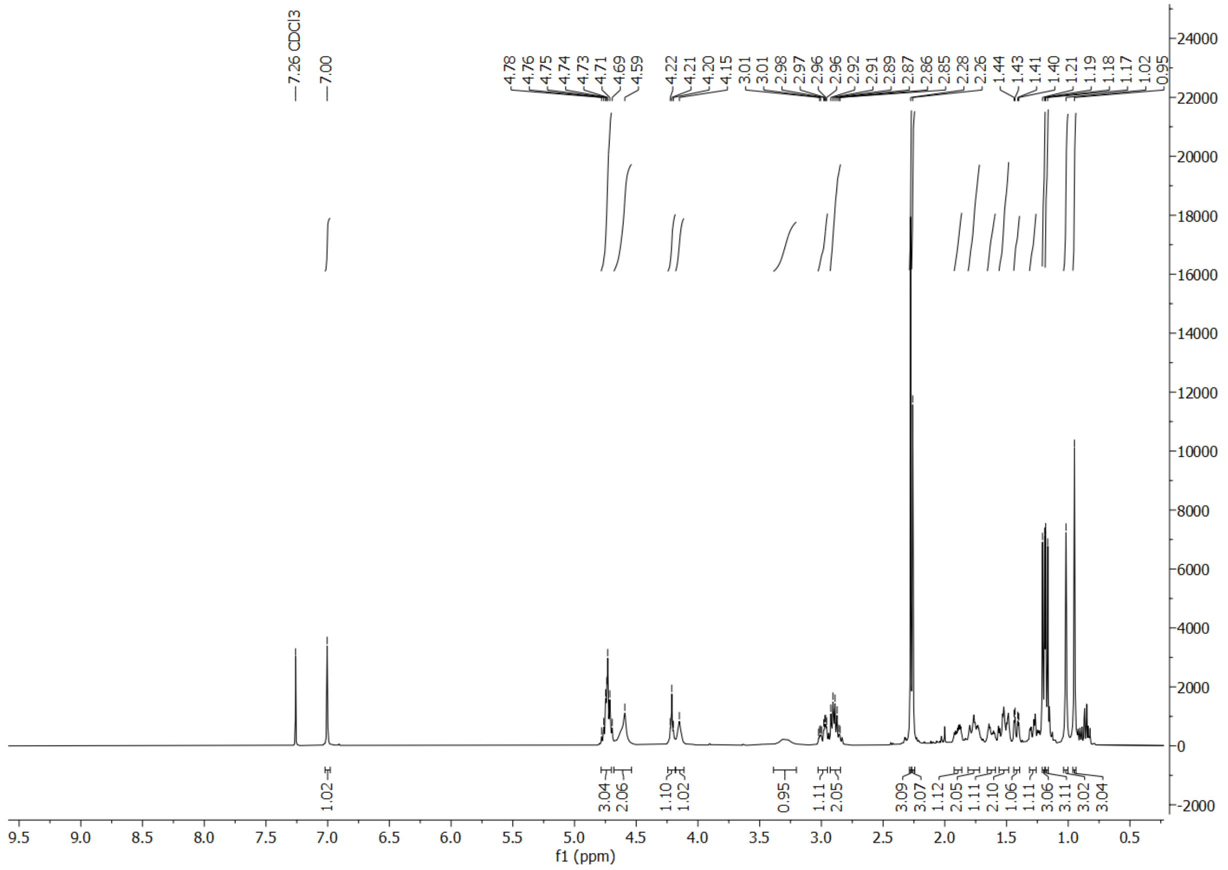

*16.2. ^{13}C spectrum for compound **19** recorded in $CDCl_3$*

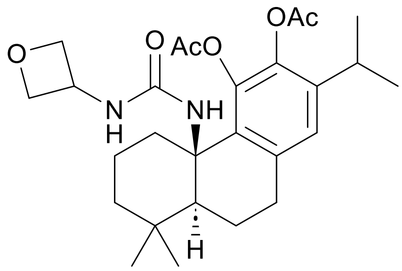

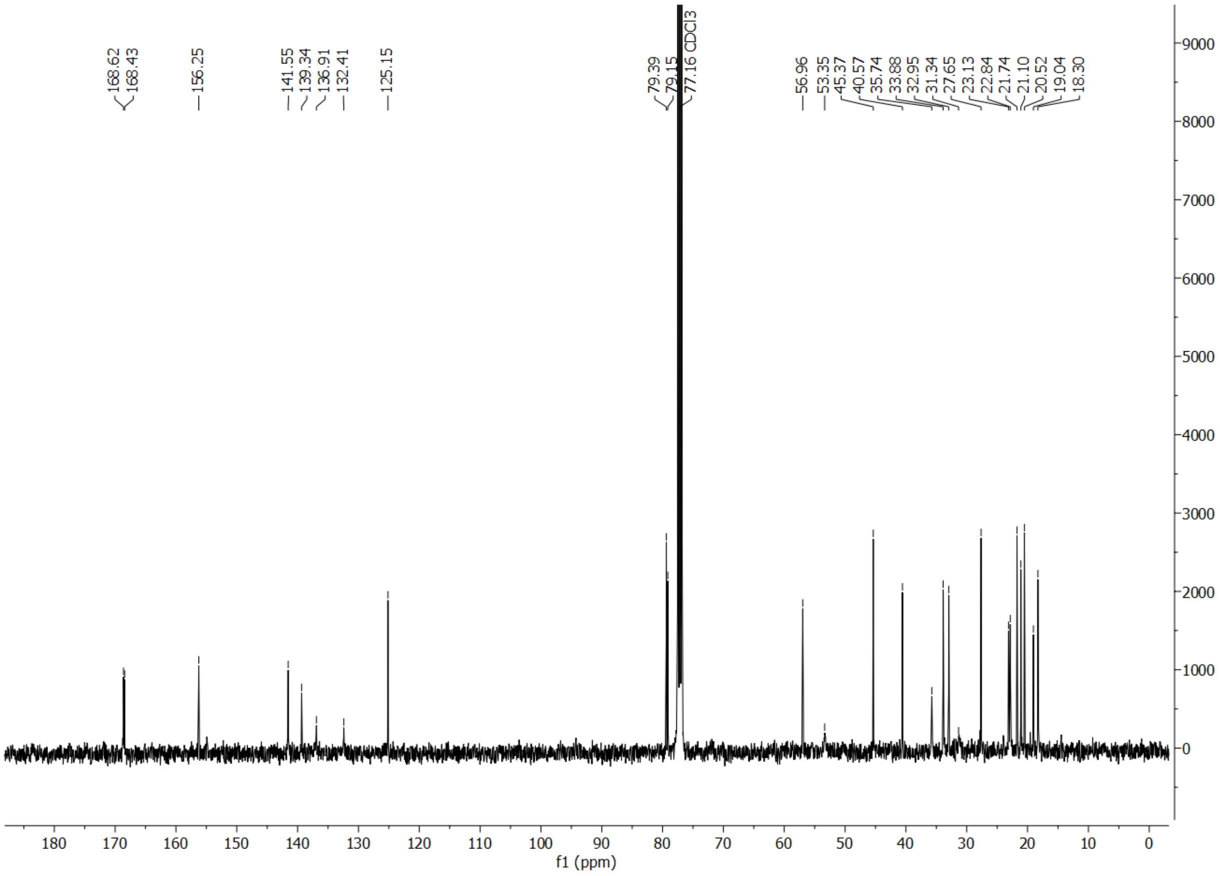

*16.3. HSQC spectrum for compound **19** recorded in $CDCl_3$*

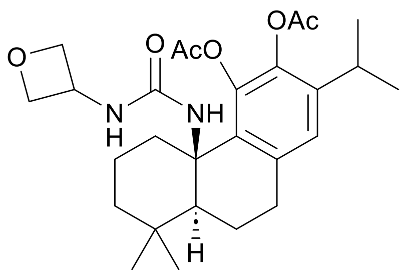

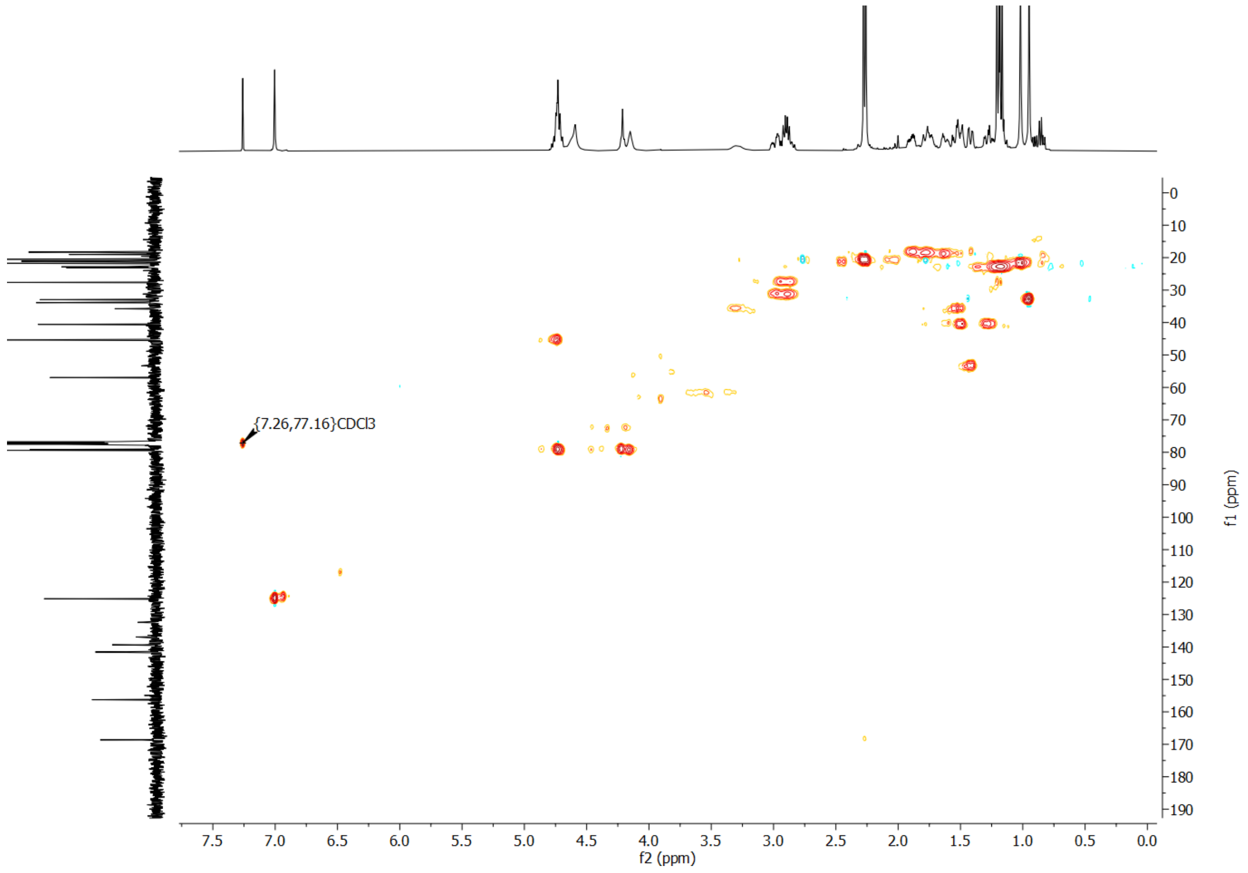

17. NMR data for compound 20

*17.1. ^{1}H spectrum for compound **20** recorded in $CDCl_3$*

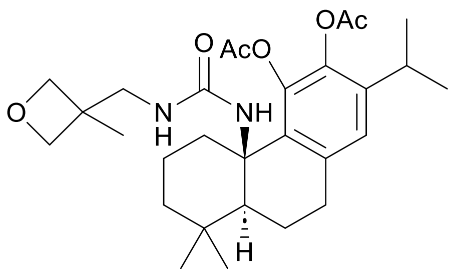

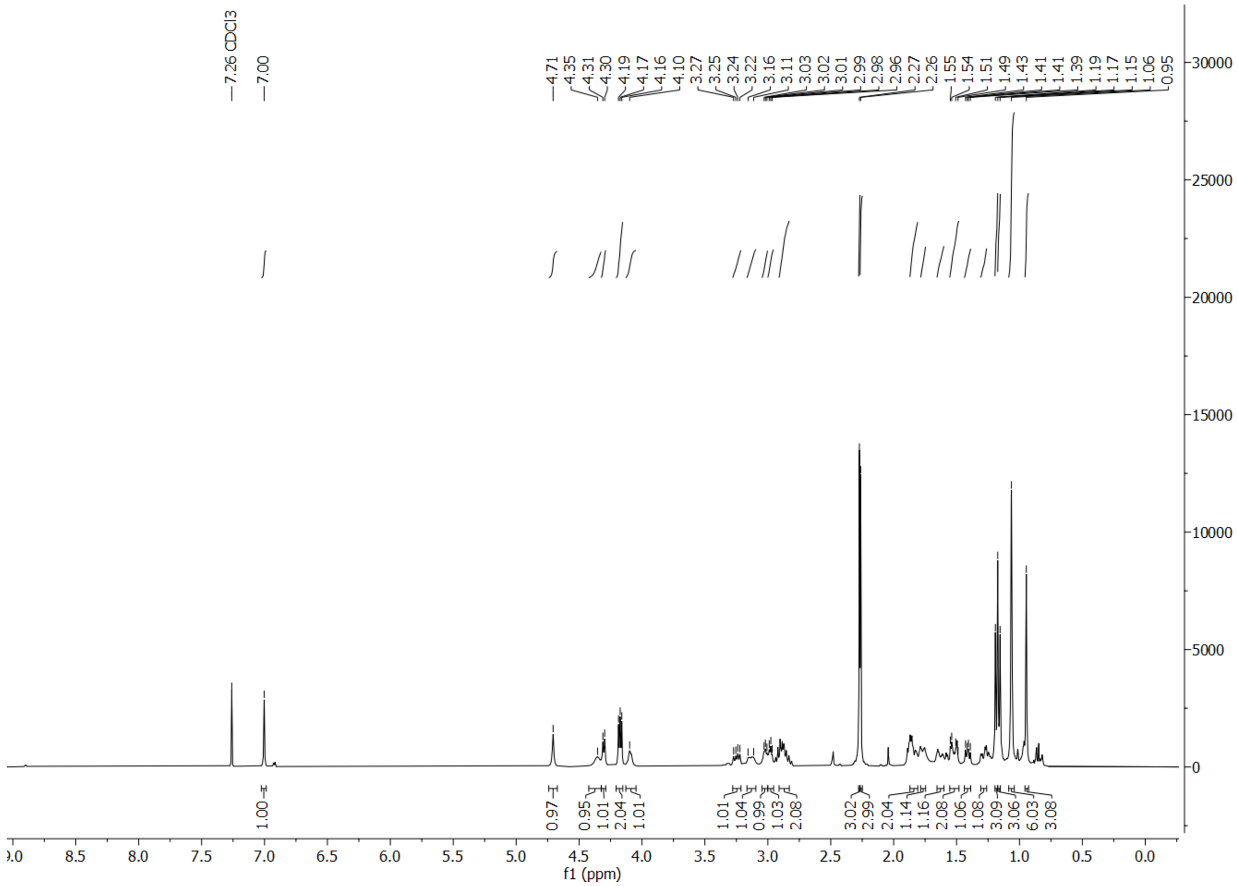

*17.2. ^{13}C spectrum for compound **20** recorded in $CDCl_3$*

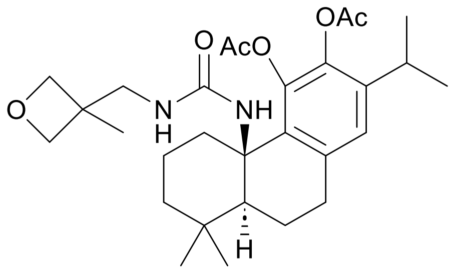

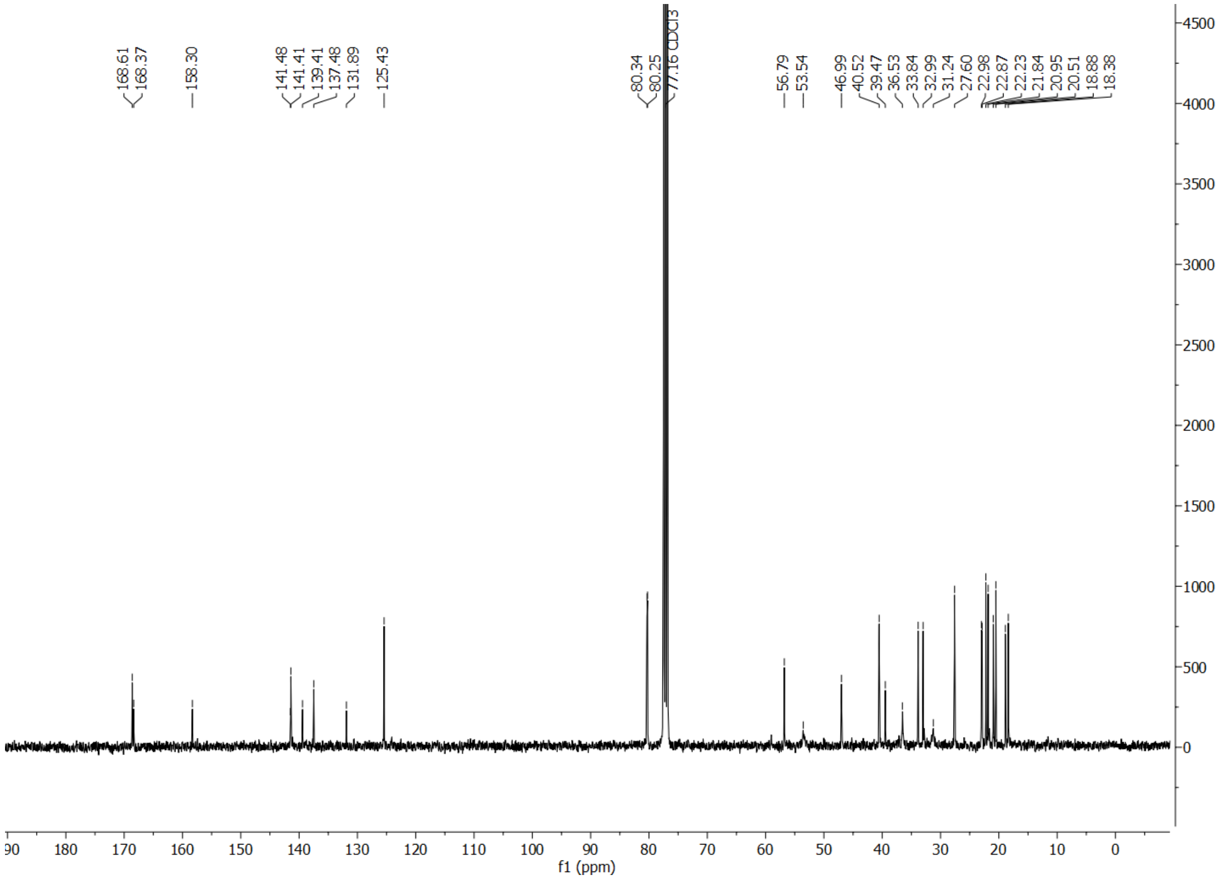

*17.3. COSY spectrum for compound **20** recorded in $CDCl_3$*

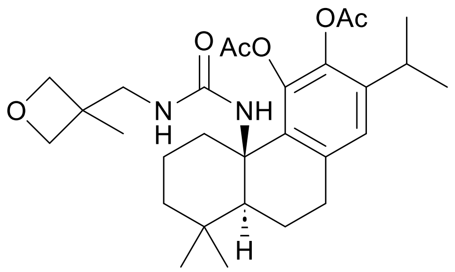

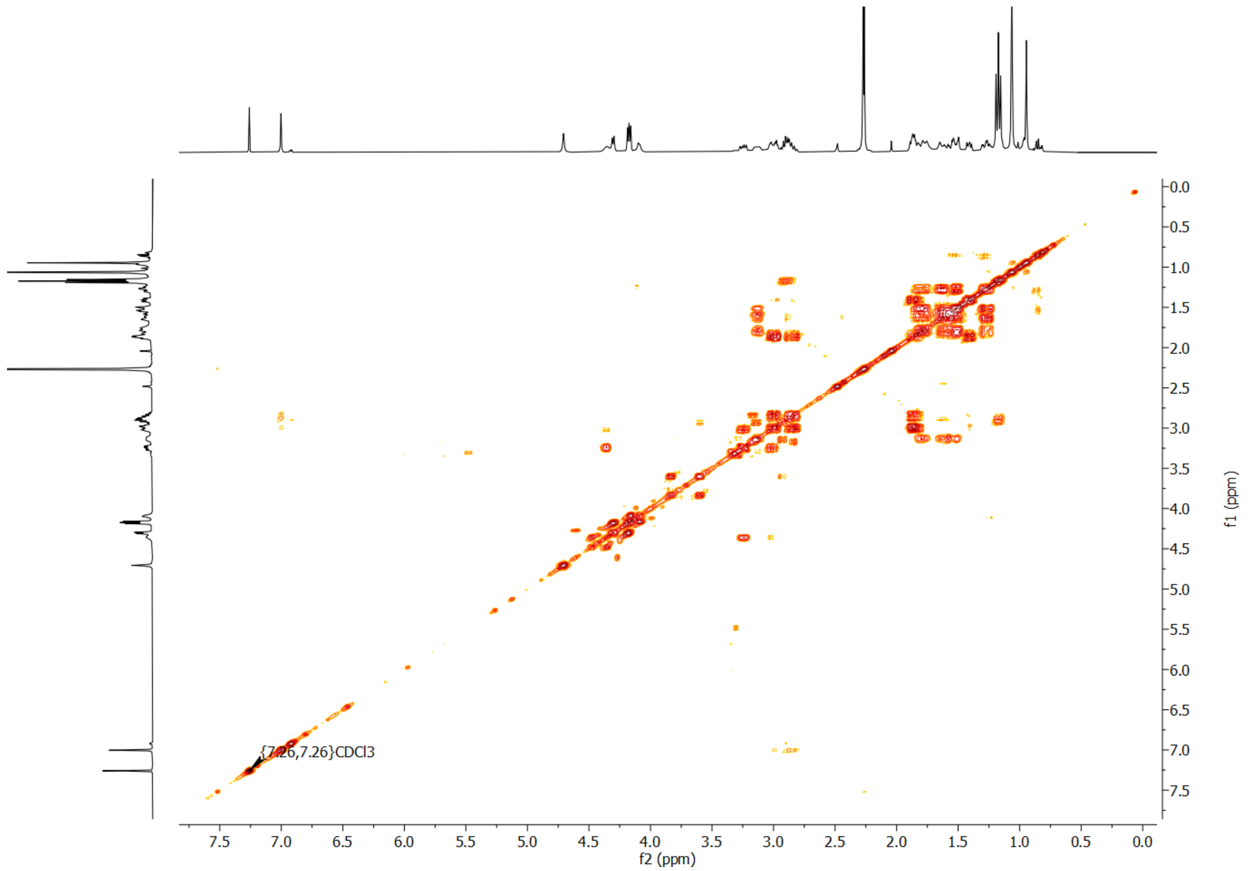

*17.4. HSQC spectrum for compound **20** recorded in $CDCl_3$*

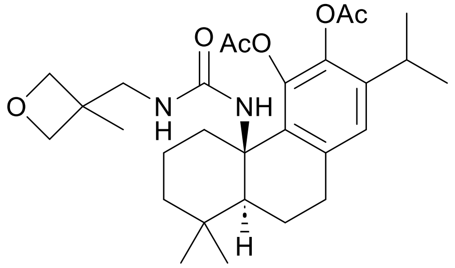

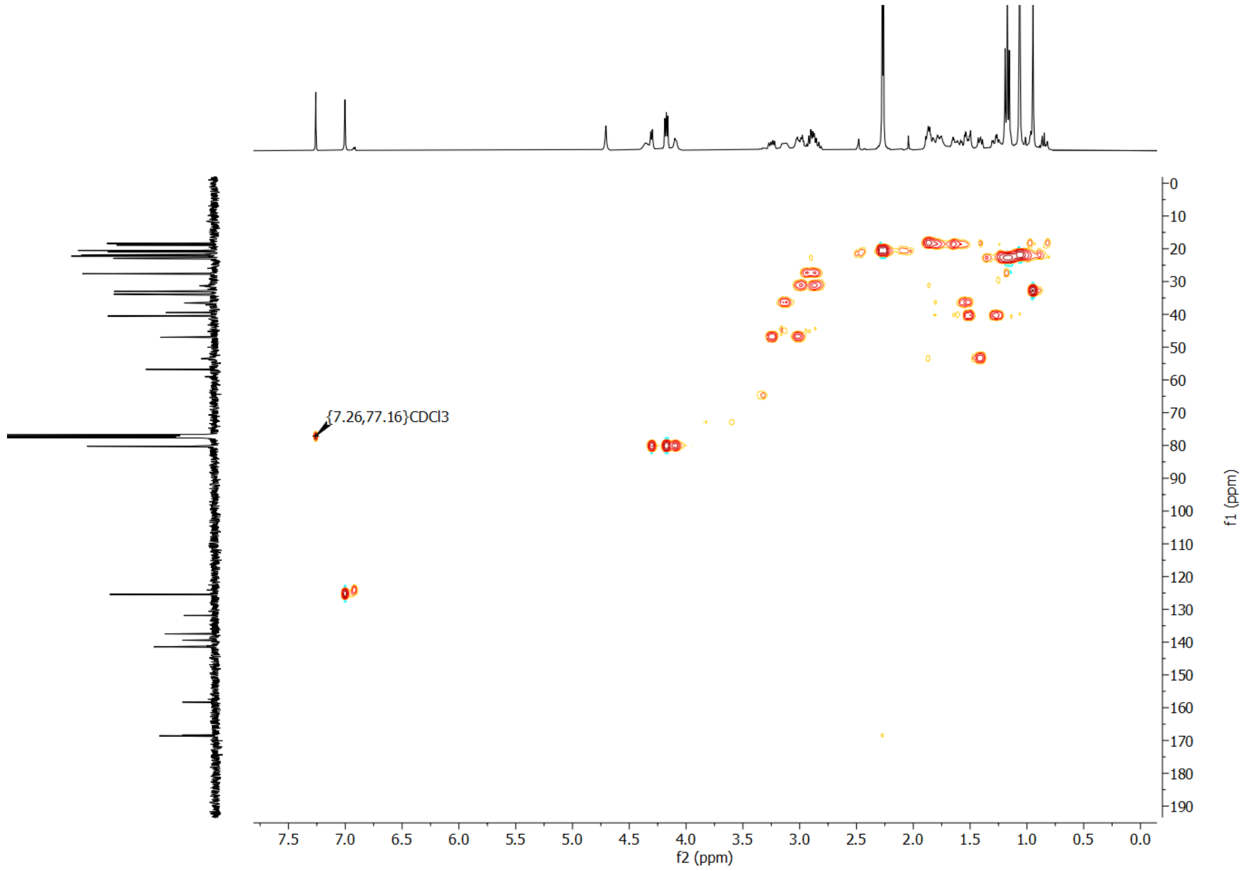

*17.5. HMBC spectrum for compound **20** recorded in CDCl₃*

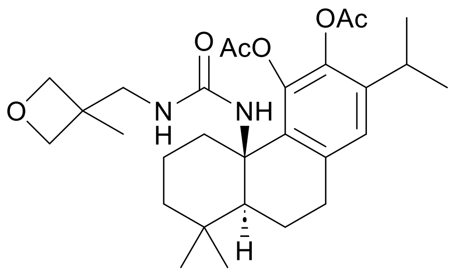

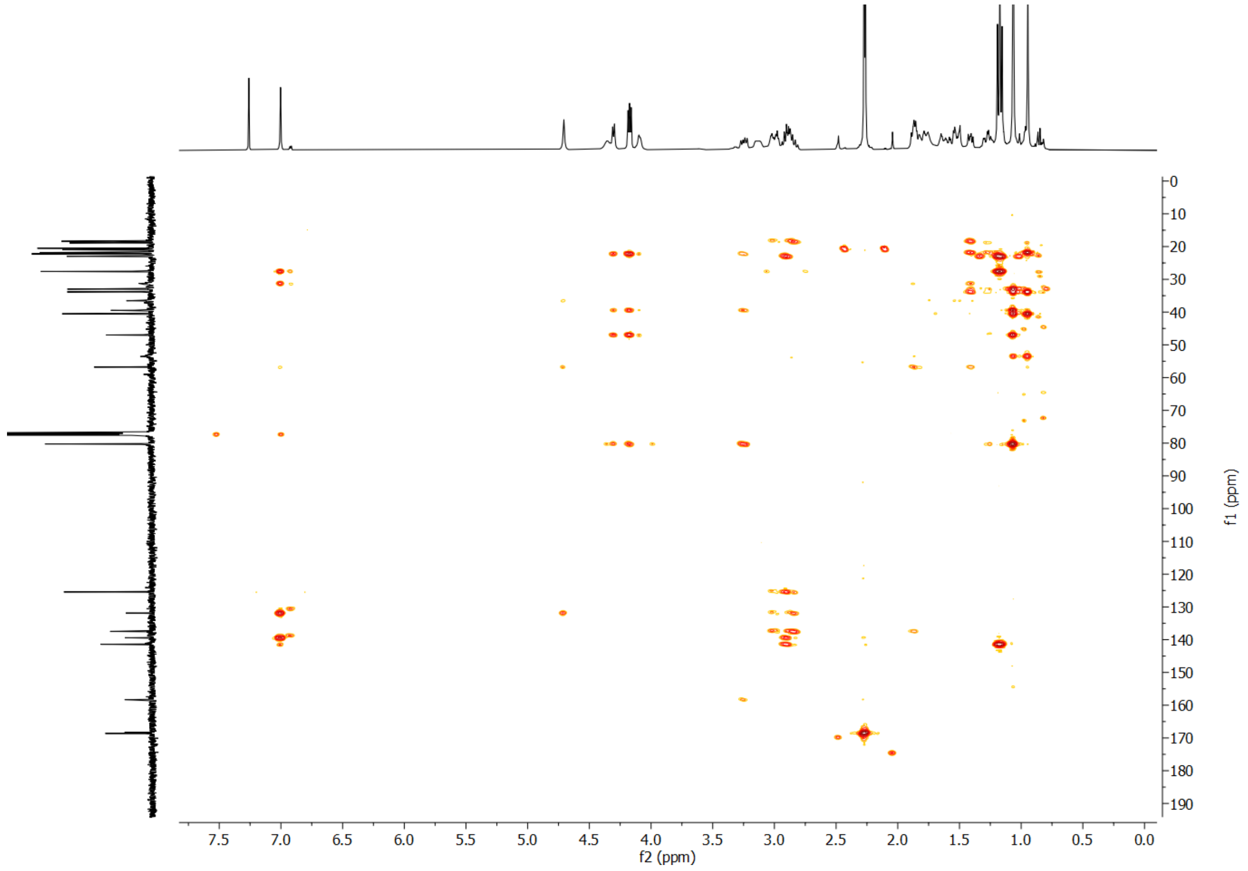

18. Uncropped and unprocessed western blot gels

Images of western blot gels of the **Figure 8**:

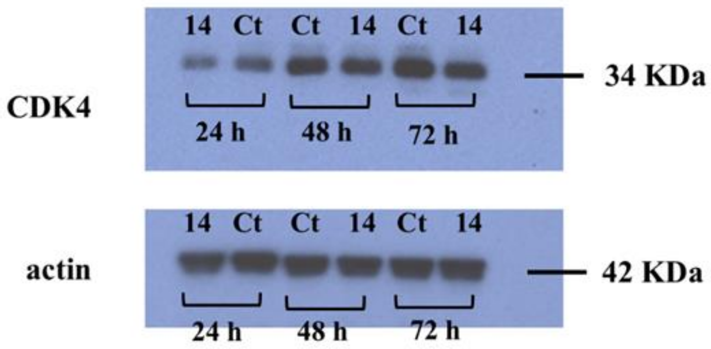

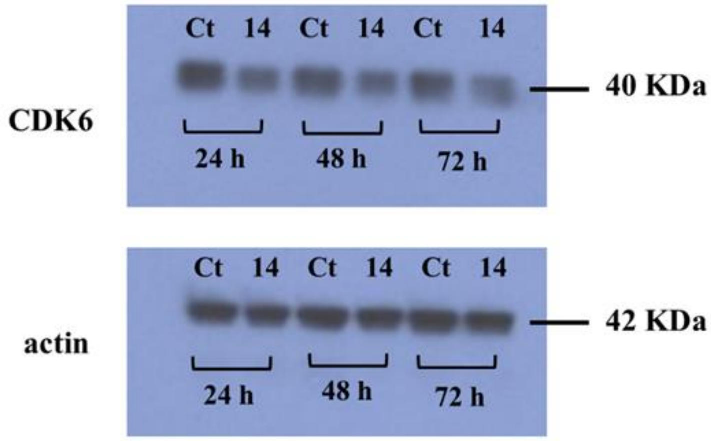

Images of western blot gels of the **Figure 12**:

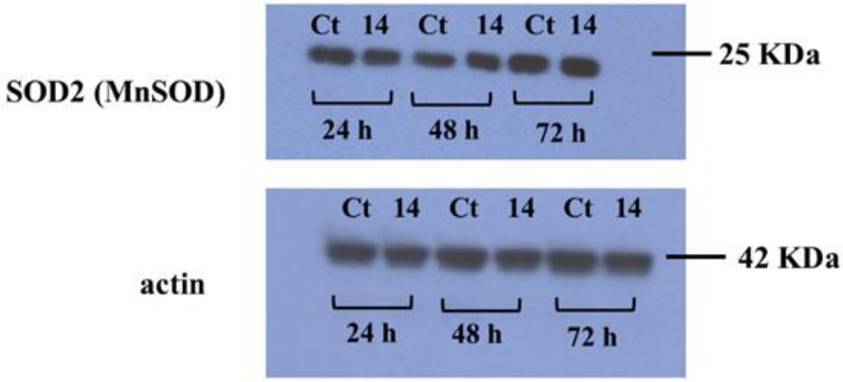

Images of the western blot gels of independent biological replicates used in the quantitative analysis are presented below:

- **CDK4**

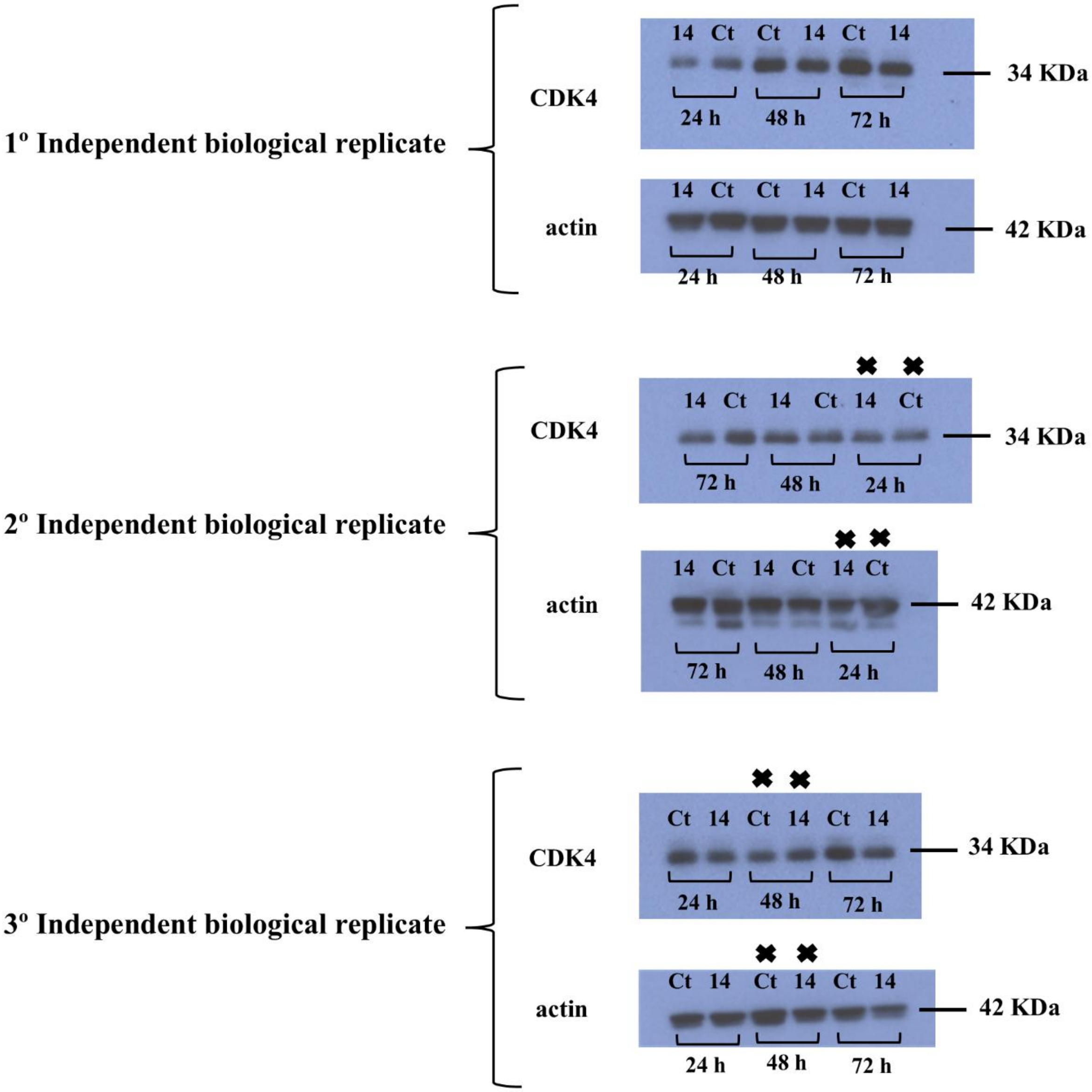

Note: The lanes marked with an "X" above the lane label on the western blot image were not included in the quantitative analysis.

- **CDK6**

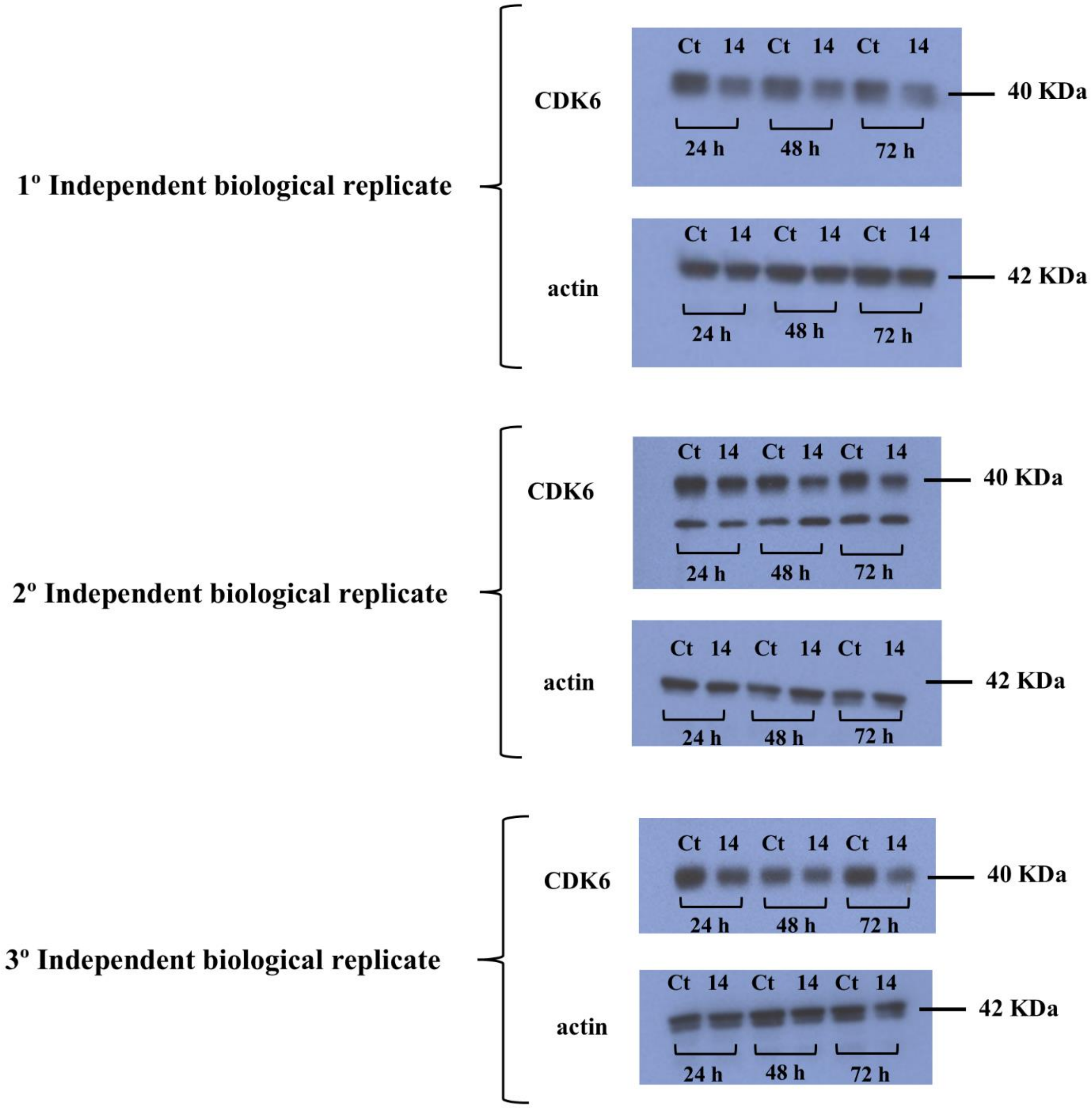
1º Independent biological replicate
CDK6
Ct 14 Ct 14 Ct 14
24 h 48 h 72 h
40 KDa
actin
Ct 14 Ct 14 Ct 14
24 h 48 h 72 h
42 KDa
2º Independent biological replicate
CDK6
Ct 14 Ct 14 Ct 14
24 h 48 h 72 h
40 KDa
actin
Ct 14 Ct 14 Ct 14
24 h 48 h 72 h
42 KDa
3º Independent biological replicate
CDK6
Ct 14 Ct 14 Ct 14
24 h 48 h 72 h
40 KDa
actin
Ct 14 Ct 14 Ct 14
24 h 48 h 72 h
42 KDa

- **SOD2 (MnSOD)**

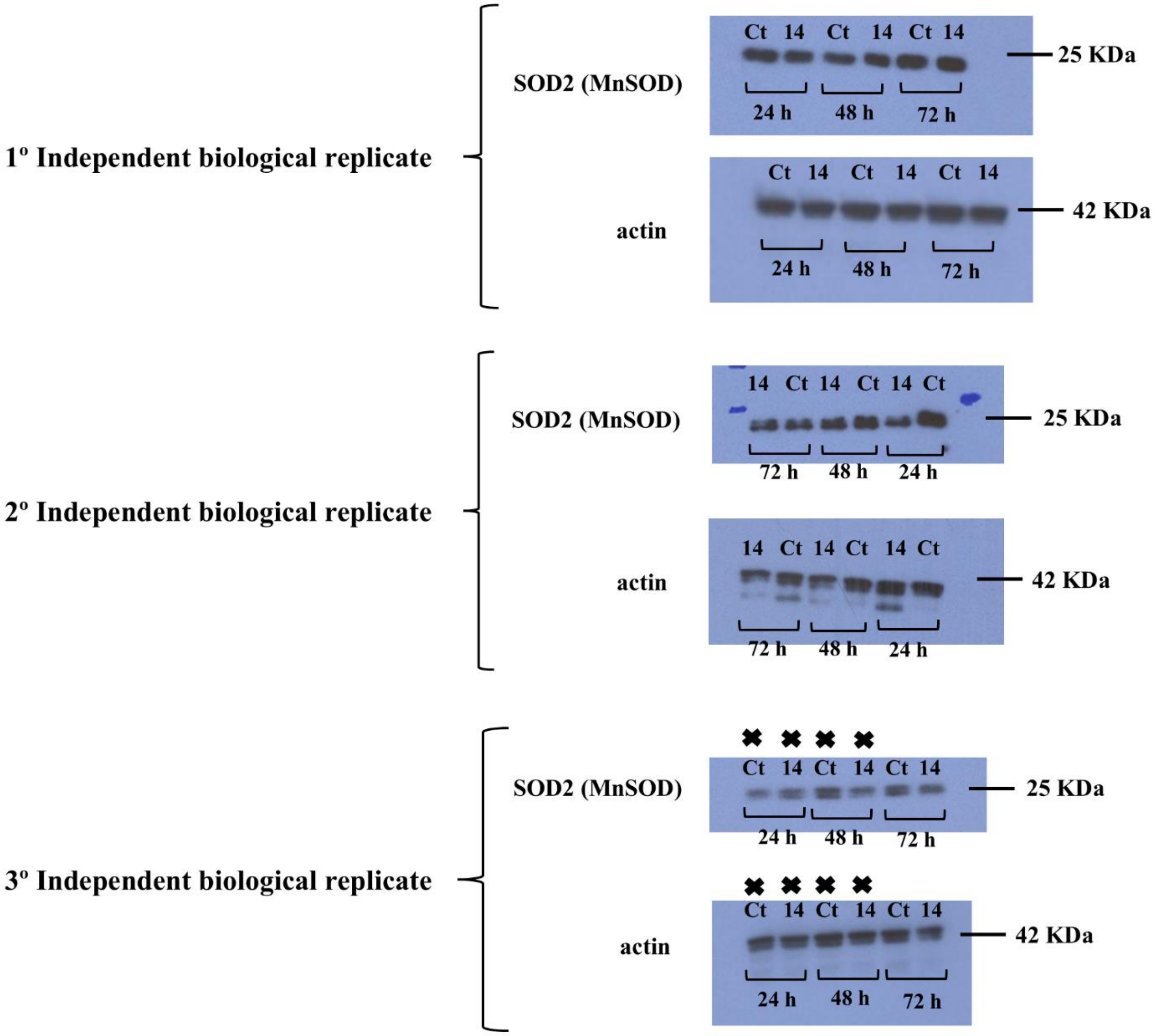

<u>Note:</u> The lanes marked with an "X" above the lane label on the western blot image were not included in the quantitative analysis.
